# Comparison of ALitretinoin with PUVA as the first-line treatment in patients with severe chronic HAnd eczema (ALPHA): study protocol for a randomised controlled trial

**DOI:** 10.1136/bmjopen-2021-060029

**Published:** 2022-02-23

**Authors:** Isabelle L Smith, Rachael Gilberts, Sarah Brown, Catherine Fernandez, Jane Nixon, Catherine Reynolds, Catherine Smith, John T Lear, Lesley Sunderland, Cathy Green, Mark Goodfield, Fiona Cowdell, Philip Hampton, Amy Barker, Armando Vargas-Palacios, Sandy Tubeuf, Miriam Wittmann

**Affiliations:** 1 Clinical Trials Research Unit, Leeds Institute for Clinical Trials Research, University of Leeds, Leeds, UK; 2 St John's Institute of Dermatology, Guy’s & St Thomas’s NHS Foundation Trust, London, UK; 3 Department of Dermatology, Salford Royal NHS Foundation Trust, Manchester, UK; 4 MAHSC, University of Manchester, Manchester, UK; 5 Street Lane Medical Practice, Leeds, UK; 6 Department of Dermatology, Ninewells Hospital, Dundee, UK; 7 Department of Dermatology, Chapel Allerton Hospital, Leeds Teaching Hospitals NHS Trust, Leeds, UK; 8 Faculty of Health Education and Life Sciences, Birmingham City University, Birmingham, UK; 9 Department of Dermatology, Newcastle Upon Tyne Hospitals NHS Trust, Newcastle Upon Tyne, UK; 10 Faculty of Biological Sciences, University of Leeds, Leeds, UK; 11 Academic Unit of Health Economics, Leeds Institute of Health Sciences, University of Leeds, Leeds, UK; 12 Institute of Health and Society, Faculté de Santé Publique, Clos Chapelle-aux-Champs, Brussels, Belgium; 13 Institute of Economic and Social Research (IRES) – LIDAM, Collège L. H. Dupriez, Louvain-la-Neuve, Belgium; 14 Leeds Institute of Rheumatic and Musculoskeletal Medicine, University of Leeds, Leeds, UK; 15 NIHR Leeds Musculoskeletal Biomedical Research Unit, Chapel Allerton Hospital, Leeds, UK; 16 Department of Dermatology, Bradford Teaching Hospitals NHS Foundation Trust, Bradford, UK

**Keywords:** dermatology, eczema, photodermatology, occupational dermatology

## Abstract

**Introduction:**

Hand eczema (HE) is one of the most common skin disorders and an important cause for morbidity and occupational disability. The 1-year prevalence of HE is estimated to be up to 10% and it is estimated that 5%–7% of those develop severe chronic HE. However, current clinical evidence is not compelling enough to guide clinical practice. In a survey among 194 UK dermatologists the most frequent first choice approaches were psoralen combined with ultraviolet A (UVA) treatment (PUVA), oral steroids and alitretinoin (AL). When asked which strategy was most efficient for long-term outcome 20% of clinicians indicated they did not know; 43% of clinicians reported AL and 30% reported PUVA.

**Methods and analysis:**

ALPHA is a multicentre, open, prospective, two-arm parallel group, randomised controlled trial comparing PUVA and AL with a planned sample size re-estimation. Between 500 and 780 participants will be randomised on a 1:1 basis. The physician’s global assessment (PGA) will direct treatment after randomisation, non-responders will be treated according to usual clinical practice; providing valuable pilot data on second line therapeutic approaches to inform future trials.

Assessments will be conducted up to 52 weeks post randomisation. The primary outcome measure is the Hand Eczema Severity Index at 12 weeks. Secondary outcome measures include modified Total Lesion Symptom Score, PGA, time to relapse, patient reported outcome measures and DNA extraction and assessment of genetic variants. A substudy on molecular inflammatory mediators will provide information on subgroup specific treatment responses. Photographs will be taken and HE severity assessed by a central review panel.

**Ethics and dissemination:**

Ethics approval was obtained from Leeds West Research Ethics Committee (14/YH/1259).Trial results will be disseminated at relevant clinical conferences and societies, published in peer-reviewed journals and through relevant patient groups.

**Trial registration number:**

ISRCTN80206075.

**Strengths and limitations of this study**

- The trial will directly compare treatment approaches used in clinical practice as first line treatment for severe hand eczema (HE), providing valuable data on their effectiveness which are not available at present.
- ALPHA takes disease subtypes and a range of factors into account known to influence response to treatment and will close an important knowledge gap on how to best treat patients with different morphologies, disease duration, allergy background and skin barrier composition with regard to filaggrin.
- This is the first interventional trial on severe HE which will compare different and previously used outcome measures for the assessment of severity meaning it will become possible to compare previous/other trials which have used different outcome measures.
- Clinical assessment of HE severity was completed by a blinded assessor, as delivery of psoralen combined with ultraviolet A and alitretinoin could not be blinded. The blinded assessor may change over time and within follow-up of the same patient; this will be reported during the final analysis.

## Introduction

### Background and rationale

Hand eczema (HE) is one of the most common skin disorders and an important cause of morbidity and occupational disability. It is estimated that up to 10% of the general population report HE at least once in a single year^1^ and 5%–7% of all patients with HE are estimated to develop severe chronic HE (CHE).^2^

The impact on daily life of sufferers is considerable.^3 4^ HE is characterised by severe itching and can be very painful and many studies have shown significant impairment of quality of life (QoL).^5 6^ HE is a persistent disease with a relapsing course and variable disease duration; in some cases at least 15 years of continuous HE have been reported.^6^

Uncertainty about the most effective treatment for HE is influenced by the fact that HE presents with several disease subgroups and underlying causes; and the natural course of HE often follows a recurrent pattern.^2 7^ The proposed classification in the UK^7^ and Europe^8^ defines atopic HE, allergic contact dermatitis and irritant dermatitis, either alone or in combination. However, discrimination of aetiologically distinct subtypes is a clinical challenge due to overlap/coexistence of different aetiologies.^7^

Moreover, it can be difficult to distinguish HE from some types of psoriasis or other skin conditions even on a histopathology level^9 10^ if patients present without involvement of other body sites and if the condition has already been treated with topical corticosteroids. Clinicians are aware of ‘mixed’ phenotypes named eczema-in-psoriatico or psoriasiform eczema^11^; which can be verified by skin biopsies, however it is not current practice to biopsy lesions in the UK. Samples obtained from the epidermis via tape stripping^12^ or washing,^13^ for example, contain markers such as IL-36, TARC, CCL20, TSLP, and IL-18 which have potential to identify eczema subtypes subtypes on a molecular level and thereby to improve subtype diagnosis.

Loss-of-function mutations in filaggrin, a protein important for the skin barrier, have been shown to be associated with atopic eczema.^14–18^ Experts have proposed^19^ that HE classification should consider filaggrin genotype for better subgrouping and potentially targeted treatment.

Alitrenoin (9-cis retinoic acid) is a naturally occurring vitamin A derivative (retinoid) and is the only licensed systemic agent for severe, CHE unresponsive to treatment with potent topical corticosteroids (National Institute for Health and Care Excellence, NICE TA177). However, there is a lack of controlled clinical trials that directly compare alitretinoin to other treatments as a first line therapy to demonstrate clinical effectiveness under daily practice conditions, which has been acknowledged by national and international expert groups.^20^

Our choice of comparator for alitretinoin was based on published clinical trials,^8^ and feedback from UK dermatologists, patients and the UK Dermatology Clinical Trials Network (UKDCTN). A survey conducted among 194 UK dermatologists informed the choice of immersion psoralen combined with ultraviolet A (PUVA) as the comparator treatment with both PUVA and alitretinion identified as popular first line therapies for severe hyperkeratotic and vesicular CHE.^21^

PUVA is used extensively across the National Health Service (NHS) and comprises a photosensitising agent in combination with UV-A. It is effective in both vesicular and hyperkeratotic HE.^22^ The photosensitising agent methoxsalen, 8-methoxypsoralen is used and the most common route of administration for this compound in the treatment of HE is topical (eg, gel, cream, immersion).^23 24^

The importance of topical PUVA was highlighted in a ‘consensus statement’ on the treatment of CHE^7^ as a widely used management option, although this is based more on clinical experience than on evidence.

Given the high socioeconomic impact of the disease and the pressing need for comparative studies on available first line treatments, ALPHA is a randomised controlled trial (RCT) comparing immersion PUVA and alitretinoin for the treatment of severe CHE.

## Methods and design

### Objectives

The overall aim of the trial is to determine the clinical and cost effectiveness of alitretinoin and immersion PUVA when used in conjunction with concomitant topical corticosteroids, emollients and patient education for the treatment of severe CHE which is unresponsive to treatment with potent topical corticosteroids alone.

The primary objective is to compare alitretinoin and immersion PUVA as first-line therapy in terms of disease activity at 12 weeks post planned start of treatment.

The secondary objectives are to compare alitretinoin to PUVA over 52 weeks postplanned start of treatment in terms of:

1. Disease activity over time.
2. Time to relapse.
3. QoL and patient benefit over time.
4. Within trial and long-term cost-effectiveness.
5. Safety.
6. Educational need for patients.

Exploratory objectives are to:

1. Compare scoring systems Hand Eczema Severity Index (HECSI), modified Total Lesion Symptom Score (mTLSS), Dermatology Life Quality Index (DLQI) and physician’s global assessment (PGA) used to monitor treatment response.
2. Explore whether response to first line treatment is affected by: duration of disease, clinical phenotype, disease severity, presence of atopy, smoking history, body mass index (BMI), foot involvement and Filaggrin loss of function mutation and other potential emerging mutations affecting skin barrier or response to treatment.
3. Collect pilot data on clinical effectiveness of second line therapies.
4. Explore treatment responses in HE subgroups defined by molecular inflammatory mediators.
5. Compare alitretinoin and PUVA in terms of time in remission.
6. Compare alitretinoin and PUVA in terms of nail assessment.
7. Explore the use of the photography guide for patients from minority ethnic groups.

### Trial design

The ALPHA trial is a multicentre, prospective, open-label, two-arm parallel group, adaptive, RCT with one planned interim analysis.

A maximum of 780 consenting participants with severe CHE will be randomised on a 1:1 basis to receive either alitretinoin or PUVA in addition to concomitant topical corticosteroids, emollients and patient education. The trial is an adaptive design with a planned interim analysis to re-estimate the final sample size.

An internal pilot phase will inform on the feasibility of recruitment and delivery of the trial.

## Methods: participants, interventions and outcomes

### Trial setting and recruitment

Patients will be screened in UK secondary care dermatology outpatient, community hospital and general practice settings. Patients complete a self-screening questionnaire on the trial website and if eligible, will be invited to a formal eligibility assessment at one of the participating research sites which will take on the responsibility for seeking consent and undertaking trial research procedures. Formal eligibility assessment and recruitment will be undertaken in secondary care dermatology outpatient clinics.

### Eligibility criteria

Patients suffering with severe CHE and unresponsive to at least 4 weeks of treatment with potent topical corticosteroids will be assessed for eligibility in accordance with the criteria in table 1.

**Table 1:** Eligibility criteria

Inclusion criteria	Exclusion criteria
Aged ≥18 years at the time of signing the informed consent form. Suffering from uncontrolled, severe CHE defined as the presence of *both* of the following criteria: (A) PGA score of severe.^27^ (B) Resistance to treatment with potent topical corticosteroids for ≥4 weeks prior to the point of eligibility screening. Provided written informed consent. Expected to comply with treatment and protocol schedule.	**Skin related:** Patients who have a clinically suspected infection (fungal, bacterial or viral) as cause for dermatitis of the hands. Patients with known clinically relevant allergic contact dermatitis of the hands unless they had made a reasonable effort to avoid the contact allergen. Patients suffering from atopic eczema covering more than 10% of body surface (excluding hands). Patients who have skin conditions worsened by the sun that is, do not tolerate UV-light (eg, lupus erythematosus, porphyria). **Treatment related:** Patients who have received phototherapy/photochemotherapy in the last 3 months prior to randomisation Patients who have received systemic vitamin A derivatives or other systemic immunosuppressants, for example, methotrexate or biologics treatment for HE in the last 3 months prior to randomisation. Patients who have received ciclosporin A or systemic glucocorticoid steroid treatment for HE within 1 week prior to randomisation. Patients receiving topical calcineurin antagonist treatment within 1 week prior to randomisation. Patients receiving concomitant treatment with tetracyclines, or medication with potential for drug–drug interaction with alitretinoin (eg, CYP3A4 inhibitor ketoconazole) that cannot be suspended or switched to an acceptable alternative Patients receiving concomitant treatment with relevant photosensitisers, when this treatment cannot be suspended for the duration of the intervention or switched to an acceptable alternative Patients with a history of melanoma skin cancer, or patients with a history of non-melanoma skin cancer depending on history, location and ‘severity’ of the non-melanoma skin cancer based on experience from routine practice. Patients who have received prior treatment with arsenic agents or ionising radiation in the treatment area (eg, hands). **General:** If female: (1) Lactating (2) Of childbearing potential (CBP): With positive pregnancy test (absence of pregnancy will be confirmed with a negative pregnancy test before randomisation). (2) Unwilling to follow pregnancy prevention programme measures* while receiving treatment and after the last dose of protocol treatment as indicated in the relevant SmPC. Patients with hepatic insufficiency (alanine aminotransferase and/or aspartate aminotransferase >2.5 times the upper limit of normal), known severe renal insufficiency, uncontrolled hyperlipidaemia (for all of the following: triglycerides, cholesterol and/or LDL cholesterol) or uncontrolled hypothyroidism in the 12 weeks period prior to randomisation. Patients with known hypersensitivity to peanut, soya or vitamin A derivatives or with rare hereditary fructose intolerance as determined by patient history. Patients currently suffering from hypervitaminose A as directed by clinical symptoms or patient history. Patients previously participated in the ALPHA trial.

Concomitant treatments not permitted are provided in online supplemental material 1.

*Rigorous contraception for people of CBP, unless exempt according to standard of care practice, is required 1 month before treatment, during the treatment period and 1 month after cessation of treatment as per usual standard practice.

CBP, child bearing potential; CHE, chronic HE; HE, hand eczema; LDL, low-density lipoprotein; PGA, physician’s global assessment; UV, ultraviolet.

10.1136/bmjopen-2021-060029.supp1 Supplementary data

### Participant timeline

#### Consent

Blood samples are required to confirm eligibility and atopy status (presence/absence of specific IgE) prior to randomisation, and research sites have the option of using a one stage process to obtain informed consent for the full trial, or a two stage consent process with separate consent for the eligibility blood sample taken up to 12 weeks prior to randomisation.

Where eligibility is indicated, a full verbal explanation of the study and patient information leaflet will be provided by the attending clinical or research team. Patients will have as long as they need to consider participation and will be given the opportunity to discuss the trial with their family and other healthcare professionals before they are invited to take part.

For patients capable of providing consent but unable to sign or otherwise mark the consent from, witnessed consent will be provided by a carer, friend/family member or a local member of the clinical team independent of the research team. A copy of the consent form is provided in online supplemental material 2.

10.1136/bmjopen-2021-060029.supp2 Supplementary data

#### Registration

All participants who consent will be registered (by an authorised member of staff at the trial research site) into the trial before any trial related procedures are performed, using the 24-hour automated registration telephone or web based system hosted by the Leeds CTRU.

#### Baseline assessment and randomisation

The baseline visit must be booked up to 7 days before the first PUVA appointment, within 12 weeks of the eligibility blood sample and after at least 1-month duration of the pregnancy prevention programme (if applicable).

At the baseline visit, eligible patients will be invited to provide informed consent for the full trial, photography sub study and biomarker sub study (for selected centres).

Eligible patients who provide full informed consent and complete the baseline assessments will be randomised into the trial by an authorised member of the research team at the site using the 24-hour telephone or web based randomisation service hosted by the Leeds CTRU.

#### Allocation

Patients will be randomised in a 1:1 allocation ratio, to receive either alitretinoin or immersion PUVA using a computer-generated minimisation programme incorporating a random element to ensure treatment groups are well balanced for the following participant characteristics: centre, diease duration (<6 months/6–24 months/>24 months), clinical phenotype (predominately hyperkeratotic/predominately vesicular/fingertip dermatitis), presence of specific IgE, DLQI (<15, ≥15), ethnicity (white/fair/dark).

If the participant is randomised to receive alitretinoin, the phototherapy department will be informed immediately that the PUVA treatment appointment will no longer be needed for this participant.

This randomisation service will also identify participants for participation in the biomarker and photography sub studies.

### Interventions

In both trial arms education on HE in using emollients, avoiding irritants and relevant contact allergens will be delivered in a standardised way prior to randomisation. The information material for participants will be based on sources used in clinical practice (British Association of Dermatology (BAD), National Eczema Society, Eczema Society patient information leaflets).

Topical corticosteroids may be used as required, as a reflection of standard clinical practice, however, it is recommended that they should belong to the ‘potent’ group.

#### Alitretinoin

Participants randomised to alitretinoin will be provided with a prescription dispensed as per standard care practice. It is anticipated that the patient will take the first dose of alitretinoin within 7 days of randomisation.

Prescriptions of alitretinoin can be written to allow up to 5 weeks supply of treatment, except people of childbearing potential following a pregnancy prevention programme whereby alitretinoin prescriptions should be limited to a 4-week supply.

According to standard clinical practice and NICE guidelines (TA177), alitretinoin will be self-administered at a starting dose of 30 mg, taken once daily with a meal for 12 weeks. After 12 weeks, participants will be assessed for their treatment response and depending on the outcome may continue alitretinoin for up to 12 more weeks.

Dose adjustment down to 10 mg or temporary cessation may occur according to standard practice in participants who suffer from related side effects such as headaches.

#### Immersion PUVA

Meladinine (methoxsalen) will be used in combination with UVA (PUVA). Meladinine 0.75% solution for local application in immersion PUVA is diluted to 3 mg/L (prepared by mixing 0.8 mL of 0.75% Meladinine solution in 2 L tap water). The participants hands are soaked for 15 min, followed by a maximum 30 min delay before UVA exposure. The UV-A radiation dose is individually tailored to the participant depending on phenotype (as per BAD guidelines^25^) and the erythematous response of the skin following treatment.

Calibration certificates for the UV-A machines are collected from centres prior to opening to ensure the same UV dose is administered across different centres.

Immersion PUVA will be administered twice weekly for 12 weeks in out-patient phototherapy departments and will be administered and supervised by specialised nurses/dermatologists according to local policy.

After 12 weeks of immersion PUVA treatment, participants will be assessed for their response to treatment and may continue immersion PUVA for up to 12 more weeks.

### Treatment pathway: criteria of response

Responders, defined as a PGA score of clear/almost clear at 12 weeks post planned start of treatment, will discontinue randomised treatment.

Partial responders, defined as a PGA score of mild/moderate at 12 weeks post planned start of treatment, will continue with randomised treatment for up to a further 12 weeks. During this second 12 weeks treatment period patients will be monitored at 4 weekly intervals and randomised treatment can be stopped at any time if the participant responds, or symptoms worsen and in the opinion of the treating clinician there is no clinical benefit to continuation.

Non-responders, defined as a PGA score of severe at 12 weeks post planned start of treatment, will discontinue randomised treatment.

In line with standard clinical practice, cessation or alteration of regimens at any time will be at the discretion of attending clinicians or the participants themselves. All randomised treatment will be discontinued at the maximum 24 weeks treatment period and participants will continue to receive ‘standard clinical practice’ and follow-up monitoring until the end of the follow-up period.

### Data collection

Clinical data and patient reported data will be collected at baseline, every 4 weeks to week 24 and 8 weekly thereafter to week 52. At each assessment a review of medication diaries will be conducted to obtain data on HE topical corticosteroid usage and any HE treatment received (in conjunction with clinical records review). Reportable adverse reactions will be collected and will exclude the following, providing they are non-serious, because they are known and common reactions; headaches and dry skin on regions of the body other than hands for alitretinoin, and mild to moderate erythema or itching of skin in PUVA treated skin locations for the PUVA arm). For participants recruited from October 2019, the follow-up period ends at 24 weeks post planned start of treatment. A full schedule of assessments is provided in online supplemental material 3.

10.1136/bmjopen-2021-060029.supp3 Supplementary data

Data will be monitored for quality and completeness by the CTRU. Missing data will be chased until received, confirmed as not available or the trial is at analysis. Data received will be linked anonymised and entered onto a secure database at CTRU in accordance with the 2018 Data Protection Act. Photographs taken will be transferred immediately by secure email to CTRU and immediately deleted from the camera once confirmation of receipt is received by the research team. The CTRU and sponsor reserve the right to intermittently conduct source data verification exercises on a sample of participants, which will be carried out by staff from the CTRU or Sponsor.

Details of biological sample management is available in online supplemental material 4.

10.1136/bmjopen-2021-060029.supp4 Supplementary data

### Blinding

The trial is open-label as participants and investigators cannot be blinded to treatment allocation due to the nature of the PUVA intervention. However, the assessment of the HE severity scores will be undertaken by a clinical assessor (research/dermatology nurse or a clinician) who is blinded to the randomised treatment and where possible, will be the same person at each assessment of a participant. Participants will be reminded not to reveal which treatment they have received to the blinded assessors in order to preserve blinding.

Photographs will be taken for 20% randomly identified, consenting participants of white ethnicity. Photographs will be taken from all consenting participants of from minority ethnic groups at each centre. Photographs will be taken at baseline and 12 weeks post planned start of treatment. A blinded central review of the photographs will be conducted by a central review panel.

### Outcomes

#### Primary outcome measure

The primary outcome is measured as the natural logarithm of the HECSI^26^ at 12 weeks postplanned start of treatment. The HECSI is a validated scoring system used to clinical assess the severity and extent of HE.^26^

#### Secondary outcome measures

- The mTLSS.^27^
- The PGA.^27^
- Time to relapse defined as the time between achieving clear/almost clear overall on the PGA to scoring 75% of their baseline HECSI.
- The DLQI.^28^
- The Patient Benefit Index for HE (PBI-HE).^29 30^
- The Person-Centred Dermatology Self-Care Index (PeDeSi).^31 32^
- The 3 level EuroQol-5 Dimension (EQ5D-3L).^33^
- A Health Resource Utilisation and Private Costs questionnaire.
- Molecular inflammatory mediators (obtained from the tape stripping sample on participants selected for the biomarker substudy).
- Clinical observational descriptive assessments of the nails (singe-centre only).
- Photographs assessed by a central review panel in line with the photographic guide.^34^
- DNA extraction and assessment of genetic variants (including filaggrin loss-of-function genetic analysis), obtained from blood sample.

### Sample size

A minimum of 500 and maximum of 780 participants are required to detect a relative difference of 1.3 (clinical opinion) in HECSI score between treatment arms at 12 weeks post planned start of treatment (80% power; two-sided 5% significance level) assuming a coefficient of variation (CV) between 1.175 and 1.7 and allowing for 20% attrition. A sample size review will be carried out after 364 participants (precision of −0.132 and +0.168 assuming CV=1.2) have reached 12 weeks post planned start of treatment, to revise the CV and the final sample size.

A sample of 100 consenting participants will be selected to take part in a biomarker sub study. Participants will be selected based on clinical phenotype and random treatment allocation to ensure 25 participants are selected from each combination of clinical phenotype (excluding fingertip dermatitis) and treatment group.

### Statistical methods

A full statistical analysis plan predefining all analyses and patient populations will be in place prior to any comparative analyses according to guidelines.^35^ The analysis results will be reported according to Consolidated Standards of Reporting Trials.^36^ The amount and reason for missingness will be assessed by treatment group, and imputation of missing data may be considered.

### Patient populations

The primary analysis will be on an intention-to-treat basis where participants will be analysed according to randomised treatment group. A per-protocol population will also be defined, which will include all eligible randomised participants who comply with their randomised treatment allocation excluding major protocol violators.

### Interim analysis

After 364 participants have reached 12 weeks post planned start of treatment, the pooled estimate of the CV for the primary endpoint will be calculated and the sample size re-estimated. This will be conducted by an independent statistician. If the re-estimated sample size indicates that the study requires fewer than 500 participants, the final sample size will be 500 participants.

### Primary endpoint analysis

#### Primary analysis

A multivariable multilevel repeated measures linear regression model will be fitted to the primary endpoint, log_e_(HECSI), adjusting for minimisation factors duration of disease, clinical phenotype, DLQI, presence of specific IgE to inhalant or other relevant allergens and ethnicity, and the covariates: smoking history, BMI, foot involvement,^37 38^ filaggrin loss of function mutation, baseline log_e_(HECSI) and treatment group. Centre, participant and participant–time interaction will be fitted as random effects. The relative difference in the HECSI score at 12 weeks post planned start of treatment, corresponding 95% CIs and p values will be reported.

### Secondary endpoint analyses

Multivariable multilevel repeated measures linear regression models of log_e_(HECSI score), mTLSS, DLQI, PeDeSi and PBI-HE, and a multilevel repeated measures ordinal logistic regression model of the PGA over time will be fitted adjusting for the minimisation factors, covariates as for the primary endpoint analysis, corresponding baseline measurement and treatment group, as fixed effects. Centre, participant and participant-time interaction will be fitted as random effects. The parameter estimates, corresponding 95% CIs and p values will be reported; contrasts for the treatment effect at 24 and 52 weeks postplanned start of treatment will also be reported.

A Cox proportional hazards (PH) model (after confirming the PH assumption is valid) will be fitted to time to relapse adjusting for minimisation factors and covariates as for the primary endpoint analysis.

AEs and SAEs classified as related to treatment, HE or resulting from administration of any research procedures will be reported descriptively.

### Exploratory endpoints

The correlation between HECSI, mTLSS, DLQI and PGA will be calculated to assess convergent validity of scoring systems used to monitor response to treatment.

Subgroup analyses will be conducted to compare treatment effects within predefined subgroups (duration of disease, clinical phenotype, disease severity, presence of atopy, filaggrin loss of function mutation, smoking history, BMI and foot involvement) and biomarkers (such as IL-36, TARC, CCL20, TSLP and IL-18). Multivariable regression models will be fitted to the response, log_e_(HECSI), treatment group and an interaction term between treatment group and the subgroup/biomarker will be included in the models to explore if there are potential differential treatment effects.

Types of, dose and, response to second line therapies will be presented descriptively using HECSI and PGA.

Definitions of the end of remission such as the time point that participants are no longer clear/almost clear will be explored in conjunction with definitions of relapse. Frequency of corticosteroid use and nail assessment data will be reported descriptively.

Agreement between the blinded assessor and the central review of photographs will be assessed using cross tabulations and kappa statistics. These will be presented by time point and by ethnicity. Where discrepancies exist, the mTLSS will be explored for further information relating to unobservable symptoms such as pain and itch.

### Health economics analysis

A within trial and a long-term cost-effectiveness model-based analysis will be undertaken. Both will consider a UK NHS and Personal Social Services perspective and a societal perspective, and use quality-adjusted life-years (QALYs) as the outcome measure. Healthcare utilisation collected as part of the follow-up will be combined with appropriate unit cost information. These will be added to the treatment costs. Societal costs will be calculated by adding healthcare costs to the costs of lost production, based on days off work combined with wage rates and other reported private costs. Utility weights for the QALYs calculation will be obtained from the EQ-5D3L. The within trial analysis will evaluate cost effectiveness at 52 weeks, while the long term analysis will be 10 years and costs and outcomes will be discounted at 3.5% per annum as recommended by NICE.^37^ The effectiveness data from the trial will be synthesised with existing evidence where it exists. Parameter uncertainty in the within trial analysis will be assessed via a non-parametric bootstrap, while uncertainty in the long-term analysis will use probabilistic sensitivity analysis. The results of both analyses will be presented as expected incremental cost effectiveness ratios; cost-effectiveness acceptability curves and expected net benefit, using the NICE threshold of £20 000 per QALY.^37^ Secondary analyses will consider alternative time horizons and alternative utility values including the mapping of DLQI values to EQ-5D using algorithms already available.^38–40^

### Patient and public involvement

The trial grant application was supported by the Leeds Dermatology Patients Panel, patient members of the UKDCTN, and the Leeds Musculoskeletal Biomedical Research Unit patient and public advocacy group. Based on the feedback received, and in recognition to what is important to patients, we put a special emphasis on long term outcomes as well as on educational aspects in the trial design.

There are patient and public involvement (PPI) representatives on the Trial Management Group and Trial Steering Committees who have provided input into the patient information sheet and other trial documentation. Both PPI representatives also provide input into the design and conduct of the trial to ensure the patient perspective is fully integrated in key decisions about the trial, delivery and interpretation/dissemination of findings.

#### Ethics and dissemination

The trial was reviewed and approved by the Yorkshire and Humber Research Ethics Committee (REC reference: 14/YH/1259), the Health Research Authority (HRA) and Medicine and Healthcare products Regulatory Agency (MHRA).

Sites will be informed of any and all major changes to the trial protocol. Any significant amendments will be reported to the REC, HRA and MHRA.

Trial results will be disseminated at relevant conferences and published in peer-reviewed journals. Authorship will be decided according to ICMJE guidelines as to qualifying contributions.

### Methods: monitoring

#### Trial governance

An independent Data Monitoring and Ethics Committee (DMEC) consisting one statistician and two clinicians will meet at least annually to review the safety and ethics of the study and the results of the interim analysis. The independent Trial Steering Committee (TSC) consisting one statistician, two clinicians and one patient representative will meet 6 monthly and provide overall supervision of the trial, including trial progress, adherence to protocol, participant safety and consideration of new information. The TSC will also review the recommendations made by the DMEC following the sample size re-estimation and other reviews of data by treatment group.

#### Confidentiality

All information collected during the course of the trial will be kept strictly confidential. Information will be held securely on paper and electronically at the CTRU. The CTRU will comply with all aspects of the Data Protection Act 2018. Participants will remain free to withdraw at any time from the trial without giving reasons and without prejudicing his/her further treatment/care. If a participant withdraws consent from further trial treatment and/or further collection of data, their data will remain on file and will be included in the final trial analysis.

#### Trial sponsor

The trial contact on behalf of the Sponsor is Clare Skinner, Faculty Head of Research Support, University of Leeds, Leeds LS2 9JT.

## Discussion

This trial will deliver information on the most effective treatment in patients with different HE situations regarding—among others—morphology, disease duration, filaggrin mutations, atopy status and ethnicity.

Despite the fact that HE often presents as a severe disease which severely impacts on patients’ working and social life there have been limited advancements regarding new treatments or identification of most effective treatments for a given patient. While this trial does not involve comparison of novel treatment strategies, it considers most known clinical factors which influence response to therapy. A main difficulty in the treatment of HE is the fact that disease subtypes are not clearly defined and/or recognisable in clinical reality. In addition outcome measures for assessment of severity are diverse and used inconsistently across different departments, countries and clinical trials. While ALPHA will not deliver an advanced precision medicine algorithm it will provide information on which treatment is best suited for patients with severe CHE, explore clinical subgroups and will also allow comparison of outcome measures.

In summary information collected within this trial will allow to close a significant knowledge gap regarding HE treatment approaches and will feed into treatment guidelines on this disease.

### Trial status

At the time of submission 441 participants have been randomised.

## Supplementary Material

Reviewer comments

Author's manuscript
