# Supplementary material for: Comparison of ALitretinoin with PUVA as the first-line treatment in patients with severe chronic HAnd eczema (ALPHA): study protocol for a randomised controlled trial
Source: BMJ Open. 2022 Feb 23;12(2):e060029. doi: 10.1136/bmjopen-2021-060029 (PMC8867308; doi:10.1136/bmjopen-2021-060029)
Supplement: Supplementary data [file bmjopen-2021-060029supp001.pdf]

SUPPLEMENTARY MATERIAL 2 - CONCOMITANT TREATMENTS

Concomitant treatments which are NOT permitted during the interventional phase of the trial are detailed below;

For all participants:

- Topical calcineurin antagonists
- New systemic corticosteroids for reasons other than hand eczema

For participants randomised to PUVA:

- Medication that may act as significant photosensitisers (e.g. tetracycline antibiotics) according to phototherapy guidelines.

For participants randomised to Alitretinoin:

- Systemic tetracycline antibiotics
- Other vitamin A derivatives.
- Other drugs with potential for drug-drug interaction (e.g. CYP3A4 inhibitor ketoconazole).

Smith IL, *et al. BMJ Open* 2022; 12:e060029. doi: 10.1136/bmjopen-2021-060029
