# Supplementary material for: Comparison of ALitretinoin with PUVA as the first-line treatment in patients with severe chronic HAnd eczema (ALPHA): study protocol for a randomised controlled trial
Source: BMJ Open. 2022 Feb 23;12(2):e060029. doi: 10.1136/bmjopen-2021-060029 (PMC8867308; doi:10.1136/bmjopen-2021-060029)
Supplement: Supplementary data [file bmjopen-2021-060029supp002.pdf]

Delete this line, then print first page of Information Sheet and Consent Form on Trust/Hospital headed paper

ALPHA
Comparing treatments for severe chronic hand eczema

Comparison of Alitretinoin with PUVA as the first line treatment in patients with severe chronic hand eczema

PARTICIPANT INFORMATION SHEET AND INFORMED CONSENT DOCUMENT

A large-print version of this sheet is available on request.

You have been invited to take part in a research study called **ALPHA**. Before you decide if you want to take part, we would like to explain why the research is being done, how we will use any information about you, and what the study will involve.

Please read this information carefully, and discuss it with others if you like. Ask us if anything is unclear, or if you would like more information.

Once you have read this information, your doctor will talk to you about the study again and you can ask any questions you like.

- Part 1 explains the purpose of this study and what will happen to you if you take part.
- Part 2 gives you more detailed information about the conduct of the study.

Please take time to decide whether or not you wish to take part.

How to contact us

If you have any questions about this study, please talk to your doctor at

<<Enter PI, nurse name >>

<< Contact details for site>>

Thank you for reading this information sheet.

Smith IL, *et al. BMJ Open* 2022; 12:e060029. doi: 10.1136/bmjopen-2021-060029

Part 1

What is the purpose of the study?

The purpose of the ALPHA research study is to find out the most effective treatment for chronic hand eczema. The two most common treatments include (1) exposure of hands to ultra violet (UV) light after they have been soaked in a solution called psoralen (a process referred to as PUVA) and (2) the tablet treatment Alitretinoin. Although both of these treatments are equally used by doctors in standard clinical practice, there is no clear evidence indicating which of these two treatments is most effective in treating which type of hand eczema. This study will directly compare these two treatments to examine both the short term and longer term effectiveness of each treatment in terms of both how good the hands heal with the treatment and how long the skin can remain clear once healed.

Why have I been chosen?

You have been approached because you are suffering from chronic hand eczema, which has not improved with strong steroid treatment for at least the last 4 weeks and has been defined as 'severe' by your doctor.

The study is taking place in several other hospitals around the UK and we are hoping that 780 participants like you who have chronic hand eczema will take part.

Do I have to take part?

No, your participation in the ALPHA trial is voluntary and you may withdraw your consent to take part at any time, without giving us a reason.

If you decide to take part you will be given this information document to keep. You will be asked to sign a consent form, but you are still free to withdraw at any time and without giving a reason. If you decide not to take part, your doctor will be happy to talk through alternative treatment options for your eczema. Your treatment and care will not be affected in any way.

What will happen to me if I take part?

The best way of finding out which is the most effective treatment is in a randomised study. 'Randomised' means that a treatment (PUVA or Alitretinoin) will be allocated to you randomly. Neither your doctor nor you will choose which treatment you receive. In this way, a fair comparison can be made.

First we need to make sure that it is safe for you to take part and that you are suitable for this study. To do this you will have some screening tests.

- **Screening visit:**

You will need to have a blood test taken (if you have not had one taken within the last 3 months) to determine whether you are suitable to take Alitretinoin or not. This is because Alitretinoin can, in some participants, increase levels of cholesterol and natural fats (lipids) within the blood, and should not be taken by participants who have significant liver problems. As we do not know at this stage which participant will receive Alitretinoin, we will collect a blood sample from all participants. This test is to make sure that it is safe for you to take Alitretinoin if you are randomised to this treatment in the study.

We will also collect a blood sample to screen for atopy which is the tendency to develop the classic allergic diseases (atopic dermatitis, allergic rhinitis (hay fever), and asthma). This is routine for all participants who have severe hand eczema. Therefore the blood sample will not be taken if the results are already available in your hospital notes.

Smith IL, *et al. BMJ Open* 2022; 12:e060029. doi: 10.1136/bmjopen-2021-060029

Your study doctor may ask you questions about your medical history and medications you are currently taking, and examine your hands to make sure you are eligible to participate.

Women of child bearing age will be asked at this visit to agree to use a reliable form of effective contraception (if you are not already doing so) and follow a strict pregnancy prevention program. This is standard care for all participants who are considered for Alitretinoin treatment. As you will not know at this stage what treatment you will be randomised to at the baseline visit, all participants will be expected to follow the strict pregnancy prevention program and use a reliable form of effective contraception for at least one month before the treatment has started. If you are then randomised to receive Alitretinoin, you will be required to continue to follow the strict pregnancy program throughout the trial and one month after the treatment has stopped.

If you are randomised to PUVA, it is not common clinical practice to follow the strict pregnancy program. However, the study doctor will advise that you should not become pregnant during the trial and recommend that you use a reliable form of contraception.

If the blood test shows that it is not suitable or safe for you to take part in this study, your doctor will discuss other treatment options with you. Any information we have collected about you will still be used but you will not continue on the study.

- **Baseline visit:**

If your screening test confirms that you are able to take part in the study, your hands will be examined by both the study doctor and an independent assessor (doctor or research nurse), who does not know your randomised treatment. You will be asked to provide another blood sample so that we can look at the genetic makeup of proteins (such as filaggrin) known to be important in eczema.

We will ask you to fill in some questionnaires about your hands and your health in general.

For women of child bearing age a pregnancy test will be done to make sure you are not pregnant.

You will then receive standard education in using moisturisers, emollients, avoiding irritants and relevant contact allergens and steroid cream use before being randomised to a treatment.

You will also be provided with a medication diary to complete, so that you can record when you have received your randomised treatment and how often you use steroid creams to control your hand eczema. In order to help you with this aspect of the study you can sign up to a text reminder service, if you wish, where you will receive a text once a week to remind you to complete your medication diary. This service is optional. It will be important that you bring your diary to each of your visits.

- **Treatment visits (interventional phase):**

If you are randomised to receive Alitretinoin, you will need to take the tablets once a day with your main meal as instructed by your study doctor for 12 weeks.

If you are randomised to receive PUVA you will attend twice weekly for PUVA treatment for 12 weeks. Please note that the bath solution that you soak your hands in for the trial PUVA treatment will be obtained from a different supplier but will be used at the same dose as the standard bath solution used in NHS practice.

Depending on how your hand eczema is responding to either treatment, you may be asked to continue treatment for up to a further 12 weeks.

During this 12 week period, you will be asked to attend clinic every 4 weeks where you will be asked to complete some questionnaires about your hands and your health, and your hands will be assessed.

- **Follow up visits**

You will be asked to attend once every 4 weeks for 24 weeks. At these visits, you will again be asked to complete some questionnaires about your hands and your health, and your hands will be assessed.

It is important that you do not tell the independent assessor which treatment you are receiving.

Optional photography

A selection of participants will be chosen at random and asked if they would like to consent to having photographs taken of their hands at the start of the study and after 12 weeks. These photographs will be reviewed to ensure that assessment of chronic hand eczema is consistent across all hospitals in the study. If you would prefer not to be photographed then you can opt out of photography, but still continue with other parts of the trial. The photographs will only be of your hands (not your face) so you can not be identified from the photographs.

How long does treatment go on?

You will receive your randomised treatment for a period of 12 weeks. At this point, if your doctor feels that your condition has cleared sufficiently, or is not responding sufficiently, then your randomised treatment will be stopped. However, if your condition has not cleared, but has shown signs of improvement then your doctor may advise that you continue your randomised treatment in the same way for up to a further 12 weeks. Your doctor will assess your condition every 4 weeks until week 24 to see if your hand eczema has got better. If this happens, you will stop receiving your randomised treatment. You will only receive your randomised treatment for a maximum of 24 weeks.

What if the treatment doesn't help?

If, after 12 or 24 weeks, your randomised treatment is not helping your condition, then your doctor will stop this treatment and treat you according to local standard practice. For example if you don't respond to Alitretinoin, then you may be switched to PUVA treatment or other standard treatments such as immunomodulators or other Vitamin A derivatives. This will be decided by your study doctor.

You will be able to use moisturisers, emollients, soap substitutes and/or bath oils on a daily basis throughout the study, and topical steroids when you feel that you need to use them to control your hand eczema.

How is my condition monitored?

Your doctor will continue to assess your skin throughout the study even if your eczema has cleared; this is so that the doctor will be able to treat your skin according to standard care practice if your eczema starts to get worse.

Unwanted effects of treatment

All the study treatments are used routinely in standard care; however there are some known side effects.

Alitretinoin:

Alitretinoin can, in some participants, increase lipid levels within the blood (30mg dose affects 1 in 3; 10mg dose affects 1:6), so your doctor will check for these with regular blood tests. Alitretinoin has been shown to cause headaches in some cases (30mg dose affects 1 in 5 participants), although these are usually eased or stopped by reducing the dose of Alitretinoin (10mg dose affects 1 in 10 participants).

Other potential side effects reported for Alitretinoin include dryness of the eyes and skin (between 1:10 and 1:100 participants), muscle and joint pain (between 1:10 and 1:100 participants) and mood swings

(only very few; the exact number is unknown). If you experience unusual depressive mood changes please discontinue the tablet and talk to your study doctor.

Alitretinoin can harm an unborn baby; therefore you must not take part in this study if you are pregnant. You must not become pregnant during the study period or for 1 month after your last study dose. If you are at risk of becoming pregnant you need to use a reliable form of effective contraception before you start treatment and during the study. If you become pregnant during the study, you must tell your study doctor at once so that your study treatment can be stopped immediately. You will be referred to an obstetrician/gynaecologist experienced in reproductive toxicity for further evaluation, counselling and advice on the potential risks to your unborn child and the options available to you. Your pregnancy will be followed closely by the study team up until final outcome.

Once you have completed the study or if you withdraw from the study and you become pregnant during the 1 month period after your last dose of study drug, you should still tell your study doctor as soon as possible.

PUVA:

There is a small risk that your skin may burn, with redness and soreness (less than 1 in 100) and very rarely blistering (less than 1 in 200, particularly in participants of fair complexion). Some people may develop a rash (polymorphic light eruption) or may experience pain and itching related to the PUVA therapy. Should that happen, your treatment will be discontinued until symptoms improve. However the risk of burning is minimised by adapting the UVA dose to your skin type and your skin response to treatment by the phototherapy staff. UV light therapy can increase the risk of skin cancer. This risk may gradually increase with long term UV application (e.g. over 200 treatments). However, a low number of exposures (48) will be provided as the randomised treatment in this trial.

PUVA treatment is not thought to cause any harm to an unborn baby. However, this information is based on limited available data. Therefore, it is recommended not to become pregnant during treatment and, if at risk of pregnancy, you should use a reliable contraception before the treatment has started, during treatment and for 3 months after the treatment has stopped, unless advised otherwise by the dermatologist or staff within the phototherapy department.

If you become pregnant during the study, you must tell your study doctor at once who will stop your study treatment immediately. Your pregnancy will be followed closely by your doctor and the study team until final outcome.

What are the possible disadvantages and risks of taking part?

Taking part in this research study involves time and commitment such as regular hospital visits for treatment and follow up visits. Although the number of treatment visits are no more than if you were receiving these treatments outside of a research study setting, the follow up visits are in addition. However, your travel expenses will be paid for the follow up visits.

The treatments that you may receive as part of the ALPHA trial are used as routine standard by the NHS and therefore there are no additional risks beyond those that you would be exposed to as part of your standard care.

What are the possible benefits of taking part?

It is hoped by taking part in this study you will respond to a treatment and have an increased quality of life. This is in line with what you would have experienced if treated according to normal NHS practice, as both treatments are used as standard NHS treatments. However, the ALPHA trial will help to understand which of these treatments, if any, is more effective in the short term, and what the long term benefits of each treatment may be.

What if I would like to take part but I have trouble with or am unable to write?

If you would like to take part but cannot or find it difficult to write, you can have a witness (e.g. a friend, a family member, or member of your healthcare team) to complete the written part of the consent for you and the questionnaires during the study. The witness will only act to help you carry out your wishes – you are free to change your mind at any time.

What if something goes wrong?

It is very unlikely that you will come to any harm as a result of taking part in this study, as both treatments are already used widely across the NHS. If you have a concern about any aspect of this study, you should ask to speak with your nurse or doctor. If you remain unhappy and wish to complain formally, you can do this through the NHS Complaints Procedure. Details about how to complain can be obtained from staff on the ward or by contacting your local Patient Advice and Liaison Service (PALS) http://www.pals.nhs.uk/ or Patient Advice & Support Service (PASS) (Scotland) or your local Trust complaints department as detailed below;

<< Insert contact details for local complaints department>>

What happens when the research study stops?

At the end of the study your doctor will discuss available ongoing treatment options with you, if required.

Will my taking part be kept confidential?

Yes. If you decide to participate in the ALPHA trial, the information collected about you will be handled strictly in accordance with the consent that you have given and also the 1998 Data Protection Act. Please refer to Part 2 for further details.

Contact Details

If you have any further questions about your illness or clinical studies, please discuss them with your doctor. If you would like further information about clinical research, the UK Clinical Research Collaboration (a partnership of organisations working together on clinical research in the UK) has published a booklet entitled 'Understanding Clinical Trials'. Contact UKCRC: Tel: 0207 670 5452; website www.ukcrc.org

This completes Part 1 of the Information Sheet. If the Information in Part 1 has interested you and you are considering participation, please continue to read the additional information in Part 2 before making any further decisions.

Part 2

What if relevant new information becomes available?

Sometimes during the course of a study, new information becomes available. If this happens your doctor will tell you about it and discuss with you whether you want to continue in the study. If you decide not to continue, your doctor will continue your care if this is necessary. If you decide to continue you may be asked to sign an updated consent form. Occasionally on receiving new information, your doctor may consider it to be in your best interest to withdraw you from further study treatment.

What will happen if I don't want to carry on with the study?

If you withdraw consent from further study treatment, information will still be collected about you and will be included in the final study analysis, unless you request otherwise. If you withdraw consent for further data collection your data collected to that point will remain on file and will be included in the final study

analysis. The ALPHA Study Team may be required to collect some limited information about any side effects you may have as a result of taking part in the trial. This will only be collected if required by the Regulatory Authorities. In line with Good Clinical Practice guidelines, at the end of the study, your data will be securely archived for a period of 15 years. Arrangements for confidential destruction will then be made.

Who has organised, reviewed and funded the research and who will be supervising it?

The ALPHA trial is being organised by the University of Leeds through the Clinical Trials Research Unit (CTRU) in collaboration with Leeds Institute of Rheumatic and Musculoskeletal Medicine, Chapel Allerton Hospital, Leeds. The study has been reviewed by the Leeds West Research Ethics Committee and the Research and Development Department situated at your hospital. The study is funded by the National Institute for Health Research (NIHR) Health Technology Assessment (HTA) programme

What if there is a problem?

Harm:

Every care will be taken in the course of this clinical trial. However, in the unlikely event that you are injured as a result of the managing organisation (University of Leeds), compensation may be available and you may have to pay your related legal costs. The hospital where you receive your treatment has a duty of care to you whether or not you agree to participate in the trial and the University of Leeds accepts no liability for negligence on the part of your hospital's employees. If you wish to complain about any aspect of the way you have been treated please contact your research doctor in the first instance.

Any claims will be subject to UK law and must be brought in the UK.

If you have private medical insurance, you should tell your insurer that you are taking part in research. They will let you know if it affects your policy.

Will my taking part in this study be kept confidential?

If you decide to participate in ALPHA, the information collected about you will be handled in accordance with the consent that you have given, the 1998 Data Protection Act and General Data Protection Regulation (GDPR, please see additional information at the end of this information sheet). The information needed for study purposes will be collected on paper forms and sent (usually using standard Royal Mail post but in some cases by fax or email) from the hospital to the Clinical Trials Research Unit (CTRU). You will be allocated a study number, which will be used along with your date of birth and initials to identify you on each paper form. The consent form will also record your NHS number; Your full name will be included on your consent form and a copy of this and your mobile telephone contact details (with your permission) will be sent to the CTRU by fax, post or encrypted email. This is to enable the CTRU to send text reminders to your mobile phone about regularly completing your medication diary. Every effort will be made to ensure that any further information about you that leaves the hospital will have your name and address removed so that you cannot be recognised from it; this information will usually be removed by a member of the study team at your hospital, but may also be removed by the CTRU upon receipt.

Your data will be entered onto a secure database held at the CTRU in accordance with the 1998 Data Protection Act and GDPR.

Your healthcare records may be looked at by authorised individuals from the research team, the University of Leeds (the study Sponsor, i.e. the organisation which takes responsibility for the initiation, management and/or financing of a clinical trial) or the regulatory authorities to check that the study is being carried out correctly.

Smith IL, *et al. BMJ Open* 2022; 12:e060029. doi: 10.1136/bmjopen-2021-060029

The information collected about you may be shared with other research teams to answer new research questions in the future. Wherever possible, information will be anonymised (i.e. your full name will not be disclosed).

Your data may be passed to other organisations (possibly in other countries where the data protection standards and laws are different to the UK) to monitor the safety of the treatment(s) that you are receiving; this data will have your name removed.

For selected participants, photographs of the hands will be sent via a secure data transfer system administered by the CTRU. Wherever possible, this data will be anonymised and your name removed.

Involvement of the General Practitioner/Family Doctor (GP):

Your GP, and the other doctors involved in your healthcare, will be kept informed of your participation in this study.

Will any genetic tests be done?

Yes. If you agree to take part in ALPHA, a blood sample will be taken at your baseline visit. This sample will be used to test whether you have particular genetic markers which influence your type of hand eczema or your response to therapy. In particular, it has been shown that genetic variations in a protein called filaggrin makes carriers more likely to suffer from chronic hand eczema. Your blood sample (labelled with only your study ID number, date of birth and initials) will be sent to researchers at the Leeds Institute of Rheumatic and Musculoskeletal Medicine, Chapel Allerton Hospital, Leeds and examined as part of the ALPHA trial in accordance with this consent. Part of the genetic material obtained from the blood sample will be sent to a University hospital laboratory in Kiel, Germany, which specialises in eczema genetics, for analysis of genetic markers linked to hand eczema. Neither the laboratory in Leeds or Kiel will know your identity, so you will not receive the results of the analysis.

The remaining part of the genetic material from your blood sample and data may be stored, and may provide a resource for future studies in the field of chronic hand eczema. If any information from this study is used to develop new research, data protection regulations will be observed and strict confidentiality maintained; your data will have your personal details removed, but will be coded so it may be linked back to your details. You will not be identified in the results of future studies; however ethical approval will be obtained for any future studies involving your data or samples .

What will happen to the results of the research study?

When the study is completed the results will be published in a medical journal, but no individual participants will be identified. The study results will be available on the CTRU ALPHA website. If you would like to obtain a copy of the published results, please ask your doctor.

If you are interested in following the progress of the study, please follow us on twitter @LICTR_Alpha.

Thank you for taking time to read this.

Smith IL, *et al. BMJ Open* 2022; 12:e060029. doi: 10.1136/bmjopen-2021-060029

Delete this line, then print on Trust/Hospital headed paper

Participant ID:	Initials:
Date of Birth:	NHS/Hospital Number:
EudraCT Number:	Principal Investigator:

ALPHA

PARTICIPANT CONSENT FORM

Please initial each box

1. I confirm that I have read and understand the information sheet for the ALPHA study and have had the opportunity to ask questions.
2. I understand that my participation in this study is voluntary and that I am free to withdraw at any time without my medical care or legal rights being affected. I understand that even if I withdraw from the above study, the data and samples collected from me will be used in analysing the results of the study and in some cases further information about any unwanted effects of my treatment may need to be collected by the study team.
3. I understand that my healthcare records may be looked at by authorised individuals from the study team, regulatory bodies or Sponsor in order to check that the study is being carried out correctly. I give permission for these individuals to have access to my information.
4. I agree to allow any information or results arising from this study to be used for healthcare and/or further medical research upon the understanding that my identity will remain anonymous wherever possible.
5. I understand that the research nurse will keep secure records at the hospital which will allow me to be followed up in hospital and at home (including name, date of birth, NHS number, hospital number, address and telephone number).

Please initial each box

6. I understand that my blood sample will be sent to the Molecular Rheumatology Laboratory at St James's University Hospital in Leeds so they can obtain genetic material. I understand that a sample of my genetic material will be sent to a specialised University research laboratory in Kiel, Germany (Department of Dermatology, University Hospital Schleswig-Holstein) for genetic analysis. I agree to the genetic material sample being stored in both locations and used for additional research investigations that form part of this study. I understand that my initials, date of birth and trial identification number will be sent with my sample (but that my identity will remain anonymous wherever possible).

7. I agree to a copy of this Consent Form containing my name, date of birth and NHS number being sent to the CTRU.

8. I consent to the storage including electronic, of personal information for the purposes of this study. I understand that any information that could identify me will be kept confidential and that no personal information that could identify me will be included in the study report or other publication.

9. I agree that my GP, or any other doctor treating me, will be notified of my participation in this study.

The following points are OPTIONAL. Even if you agree to take part in this study, you do not have to agree to this section.

Please initial each box

	Yes	No
10. I give permission for surplus genetic material obtained from my blood sample may be used for further medical research upon the understanding that my identity will remain anonymous wherever possible. Only my initials, date of birth and trial identification number will be stored with my sample.	☐	☐
11. I give permission for the responses that I give on the PBI-HE questionnaire to be sent to Augustin AG, Germany, to support PBI-HE questionnaire research upon the understanding that my identity will remain anonymous wherever possible.	☐	☐
12. I agree to my mobile telephone contact details being sent to the CTRU for the purposes of sending me text messages as a reminder to complete my medication diary.<br>Enter mobile tel number: ………………………………………………	☐	☐
13. I agree to allow the research nurse to take photographs of my hands if selected at random.	☐	☐

Please initial the box

14. I agree to take part in the study**.** ☐

Smith IL, *et al. BMJ Open* 2022; 12:e060029. doi: 10.1136/bmjopen-2021-060029

Participant:
Signature…………………………………………………………………………………

Name (block capitals)……………………………………………………………………

Date…………………………………………………………………………………………

Investigator:
I have explained the study to the above named participant and he/she has indicated his/her willingness to participate.

Signature…………………………………………………………………………………

Name (block capitals)……………………………………………………………………

Date…………………………………………………………………………………………

(If used)Translator:
Signature…………………………………………………………………………………

Name (block capitals)……………………………………………………………………

Date…………………………………………………………………………………………

Witness:
I have completed this consent form on behalf of the person named above who has freely given their consent to participate.

Signature…………………………………………………………………………………

Name (block capitals)……………………………………………………………………

Date…………………………………………………………………………………………

(1 copy for participant; 1 for the CTRU; 1 held in participant notes,
original stored in Investigator Site File)

Smith IL, *et al. BMJ Open* 2022; 12:e060029. doi: 10.1136/bmjopen-2021-060029

This information is for people who are taking part in the study:

Comparison of **AL**itretinoin with **P**UVA as the first line treatment in patients with severe chronic **HA**nd eczema

We wanted to contact you to make sure you understand how we use the information we collect about you in the ALPHA study. This is because a new data protection law (called the General Data Protection Regulation, or GDPR for short) came into force in the UK on 25th May 2018. The GDPR means that you must have clear information about how information about you is collected and used.

We want to give you some more information about how the data we collect from you is used, in addition to the patient information sheet you have been given.

- University of Leeds is the Sponsor for the ALPHA study based in the United Kingdom. University of Leeds have asked the Clinical Trials Research Unit (CTRU) at the University of Leeds to run the ALPHA study on their behalf.
- CTRU will be using information from you and your medical records in order to undertake this study and will act as data controller for this study. This means that we are responsible for looking after your information and using it properly. University of Leeds will keep identifiable information about you for 15 years after the study has finished.
- Your rights to access, change or move your information are limited, as we need to manage your information in specific ways in order for the research to be reliable and accurate. If you withdraw from the study, we will keep the information about you that we have already obtained. To safeguard your rights, we will use the minimum personally-identifiable information possible.
- As a University we use personally-identifiable information to conduct research to improve health, care and services. As a publicly-funded organisation, we have to ensure that it is on the public interest when we use personally-identifiable information from people who have agreed to take part in research. This means that when you agree to take part in a research study, we will use your data in the ways needed to conduct and analyse the research study.
- Health and care research should serve the public interest, which means that we have to demonstrate that our research serves the interests of society as a whole. We do this by following the UK Policy Framework for Health and Social Care Research.
- If you wish to raise a complaint on how we have handled your personal data, you can contact our Data Protection Officer who will investigate the matter. If you are not satisfied with our response or believe we are processing your personal data in a way that is not lawful you can complain to the Information Commissioners Office (ICO).
- You can find out more about how we use your information by contacting the University of Leeds Data Protection Officer at
    - Email: DPO@leeds.ac.uk
    - Post: University of Leeds, 11.72 EC Stoner Building, Leeds LS2 9JT
    - Telephone: +44 (0)113 243 1751

- Your hospital will keep your contact details confidential and will not pass this information to CTRU (unless you have agreed to receive text reminders – see below). Your hospital will use this information as needed, to contact you about the research study, and make sure that relevant information about the study is recorded for your care, and to oversee the quality of the study. Certain individuals from the University of Leeds, CTRU and regulatory organisations may look at your medical and research records to check the accuracy of the research study. Your hospital will keep identifiable information about you from this study for 15 years after the study has finished.
- If you have agreed to receive text reminders CTRU will receive the telephone number you have provided and keep this information for 15 years after the study has finished. No other contact details will be shared.
- CTRU will receive your name and NHS number on the consent form you have signed. The only people at CTRU who will have access to information that identifies you will be people who audit the data collection process. The people who analyse the information will not be able to identify you and will not be able to find out your name. CTRU will keep identifiable information about you from this study for 15 years after the study has finished.
- When you agree to take part in a research study, the information about your health and care may be provided to researchers running other research studies in this organisation and in other organisations. These organisations may be universities, NHS organisations or companies involved in health and care research in this country or abroad. Your information will only be used by organisations and researchers to conduct research in accordance with the UK policy Framework for Health and Social Care Research.
- This information will not identify you and will not be combined with other information in a way that could identify you. The information will only be used for the purpose of health and care research, and cannot be used to contact you or to affect your care. It will not be used to make decisions about future services available to you such as insurance.

We would like to take this opportunity to thank you again for taking the time to read this information.

On behalf of the ALPHA study team

Dr Miriam Wittman
ALPHA Chief Investigator

Smith IL, *et al. BMJ Open* 2022; 12:e060029. doi: 10.1136/bmjopen-2021-060029
