# Supplementary material for: Comparison of ALitretinoin with PUVA as the first-line treatment in patients with severe chronic HAnd eczema (ALPHA): study protocol for a randomised controlled trial
Source: BMJ Open. 2022 Feb 23;12(2):e060029. doi: 10.1136/bmjopen-2021-060029 (PMC8867308; doi:10.1136/bmjopen-2021-060029)
Supplement: Supplementary data [file bmjopen-2021-060029supp003.pdf]

SUPPLEMENTARY MATERIAL 1

Table 2 ALPHA Trial Assessment Schedule

Weeks		0	4	8	12	16	20	24	28	32	36	44	52
Trial period	Screening	Baseline	Interventional			Interventional/ Follow-up			Follow-up				
Informed Consent (Full/Shortened/Optional photography)	X	X											
Registration	X												
Eligibility blood sample & IgE tests	X												
Start contraception prevention program (if applicable)	X	X											
Eligibility assessment		X											
Medical history, Clinical assessment, Gene Variant analysis blood sample		X											
PeDeSI by treating clinician/unblinded nurse		X			X								X
PGA by treating clinician		X	X	X	X	X	X	X	X	X	X	X	X
PGA, HECSI by blinded assessor		X	X	X	X	X	X	X	X	X	X	X	X
mTLSS by blinded assessor		X			X			X			X		X
Nail assessment by blinded assessor (Bradford recruited participants only)		X	X	X	X	X	X	X	X	X	X	X	X
DLQI		X	X	X	X	X	X	X	X	X	X	X	X
PBI-HE, EQ-5D-3L		X			X			X			X		X
Health resource utilisation questionnaire					X			X			X		X
Provide standard education for HE		X											
Randomisation		X											
Tape stripping sample collection (for randomly selected participants only)		X											
Photograph hands		X			X								
Randomised treatment compliance (medication diary review)			X	X	X	X	X	X					
Topical corticosteroid usage (medication diary review)			X	X	X	X	X	X	X	X	X	X	X
Details of treatment under ‘standard clinical practice’						X	X	X	X	X	X	X	X
Reportable Adverse reactions and Related SAEs/SARs/SUSARs			X	X	X	X	X	X	X	X	X	X	X
