# Supplementary material for: Comparison of ALitretinoin with PUVA as the first-line treatment in patients with severe chronic HAnd eczema (ALPHA): study protocol for a randomised controlled trial
Source: BMJ Open. 2022 Feb 23;12(2):e060029. doi: 10.1136/bmjopen-2021-060029 (PMC8867308; doi:10.1136/bmjopen-2021-060029)
Supplement: Supplementary data [file bmjopen-2021-060029supp004.pdf]

SUPPLEMENTARY MATERIAL 3 – HANDLING OF BIOLOGICAL SAMPLES

Consent

Informed, written consent for the blood sample/s must be obtained prior to registration and prior to the participant undergoing procedures that are specifically for the purposes of the trial and are not standard routine care at the participating sites.

Gene variant analysis blood sample

A blood sample is taken at baseline (although also possible at any later visit) in order to obtain DNA for subsequent analysis for the filaggrin mutation and other skin barrier molecule polymorphisms. These samples will be anonymised and sent to Professor Ann Morgan's Molecular Rheumatology laboratory (Leeds Institute Cardiovascular and Metabolic Medicine, The LIGHT Laboratories, Clarendon Way, Leeds) for DNA extraction.

For blood samples that have been damaged or classed as unusable during transit to the laboratory, the CTRU will contact research sites to request a replacement sample is obtained, if possible, from the participant at the next trial visit.

A proportion of extracted DNA will be sent to Professor Stephan Weidinger's laboratory (Department of Dermatology, University Hospital Schleswig-Holstein, Kiel, Germany) for assessment of skin barrier molecule polymorphisms (including filaggrin loss-of-function mutation). The remainder of the extracted DNA will be placed in long term storage (under suitable conditions) in the laboratory of Anne Morgan for potential future research. For example, there is evidence linking PUVA treatment and mutations in the vitamin D receptor and this sample set will provide an invaluable resource to delineate any causal link.

Biomarker sub study

A subset of 100 participants recruited from selected centres will be asked to provide written informed consent to this optional sub study to consent to the following procedures, which will be collected prior to the start of the randomised treatment:

- **Tape stripping technique** involves an adhesive to bind and remove the top epidermal layers. This approach has been used as a non-invasive technique for direct sampling of skin [17, 18]. Tape stripping is generally rapid, and patient-friendly. For detailed instructions please refer to the biomarker sub study work instruction. Tape strips will be submersed in buffer solution and stored at site at -80°C prior to sending to Dr Miriam Wittmann's laboratory (Section of Musculoskeletal Disease, Leeds Institute of Molecular Medicine, Wellcome Trust Brenner Building, St. James's University Hospital, Leeds).

- **Skin washing:** Where tape stripping is not possible due to acute inflammation, mediator content can be measured in skin washing fluids [47]. For detailed instructions please refer to the biomarker sub study work instruction. Buffer scrubs (washing fluid) will be stored at site at -80°C prior to sending to Dr Miriam Wittmann's laboratory (Section of Musculoskeletal Disease, Leeds Institute of Molecular Medicine, Wellcome Trust Brenner Building, St. James's University Hospital, Leeds, UK)
