# Supplementary material for: Comparison of ALitretinoin with PUVA as the first-line treatment in patients with severe chronic HAnd eczema (ALPHA): study protocol for a randomised controlled trial
Source: BMJ Open. 2022 Feb 23;12(2):e060029. doi: 10.1136/bmjopen-2021-060029 (PMC8867308; doi:10.1136/bmjopen-2021-060029)
Supplement: Author's manuscript [file bmjopen-2021-060029.draft_revisions.pdf]

BMJ Open is committed to open peer review. As part of this commitment we make the peer review history of every article we publish publicly available.

When an article is published we post the peer reviewers' comments and the authors' responses online. We also post the versions of the paper that were used during peer review. These are the versions that the peer review comments apply to.

The versions of the paper that follow are the versions that were submitted during the peer review process. They are not the versions of record or the final published versions. They should not be cited or distributed as the published version of this manuscript.

BMJ Open is an open access journal and the full, final, typeset and author-corrected version of record of the manuscript is available on our site with no access controls, subscription charges or pay-per-view fees (http://bmjopen.bmj.com).

If you have any questions on BMJ Open's open peer review process please email info.bmjopen@bmj.com

BMJ Open

Comparison of ALitretinoin with PUVA as the first line treatment in patients with severe chronic HAnd eczema (ALPHA): study protocol for a randomised controlled trial

Journal:	*BMJ Open*
Manuscript ID	bmjopen-2021-060029
Article Type:	Protocol
Date Submitted by the Author:	09-Dec-2021
Complete List of Authors:	Smith, Isabelle; University of Leeds Clinical Trials Research Unit,<br>Gilberts, Rachael; University of Leeds, Clinical Trials Research Unit<br>Brown, Sarah; University of Leeds Clinical Trials Research Unit<br>Fernandez, Catherine; University of Leeds Clinical Trials Research Unit<br>Nixon, Jane; University of Leeds Clinical Trials Research Unit<br>Reynolds, Catherine; University of Leeds Clinical Trials Research Unit<br>Smith, C; St John's Institute of Dermatology<br>Lear, John; Manchester Royal Infirmary, Department of Dermatology<br>Sunderland, Lesley; Street Lane Medical Practice<br>Green, Cathy; Ninewells Hospital, Dermatology Department<br>Goodfield, Mark; Leeds Teaching Hospitals NHS Trust, Department of Dermatology<br>Cowdell, Fiona; University of Hull, Faculty of Health and Social Care<br>Hampton, Philip ; Newcastle Upon Tyne Hospitals NHS Trust, Department of Dermatology<br>Barker, Amy; University of Leeds, Faculty of Biological Sciences<br>Vargas-Palacios, Armando; University of Leeds Leeds Institute of Health Sciences,<br>Tubeuf, Sandy; Universite catholique de Louvain<br>Wittmann, Miriam; University of Leeds Faculty of Medicine and Health, Rheumatic and Musculoskeletal Medicine
Keywords:	DERMATOLOGY, Eczema < DERMATOLOGY, Photodermatology < DERMATOLOGY, Occupational dermatology < DERMATOLOGY

SCHOLARONE™
Manuscripts

Comparison of ALitretinoin with PUVA as the first line treatment in patients with severe chronic HAnd eczema (ALPHA): study protocol for a randomised controlled trial

Isabelle L Smith*[1], Rachael Gilberts[1], Sarah Brown[1], Catherine Fernandez[1], Jane Nixon[1], Catherine Reynolds[1], Catherine Smith[2], John T Lear[3], Lesley Sunderland[4], Cathy Green[5], Mark Goodfield[6], Fiona Cowdell[7], Philip Hampton[8], Amy Barker[9], Armando Vargas-Palacios[10], Sandy Tubeuf[11,12], Miriam Wittmann[13,14,15,]

*corresponding author

Email Addresses

Isabelle L Smith: i.l.smith@leeds.ac.uk; Rachael Gilberts: R.M.Gilberts@leeds.ac.uk; Sarah Brown: medsbro@leed.ac.uk; Catherine Fernandez: c.fernandez@leeds.ac.uk; Jane Nixon: j.e.nixon@leeds.ac.uk ; Catherine Reynolds: c.reynolds@leeds.ac.uk; Catherine Smith: Catherine.smith@kcl.ac.uk; John Lear: john.lear@cmft.nhs.uk; Lesley Sunderland: LesleyS@nhs.net; Cathy Green: cathy.green@nhs.scot; Mark Goodfield: mark.goodfield@leedsth.nhs.uk; Fiona Cowdell: Fiona.Cowdell@bcu.ac.uk; Philip Hampton: philip.hampton@nhs.net; Amy Barker: a.m.barker@leeds.ac.uk; Armando Vargas-Palacios A.Vargas-Palacios@leeds.ac.uk; Sandy Tubeuf: sandy.tubeuf@uclouvain.be; Miriam Wittmann: m.wittmann@leeds.ac.uk

Affiliations

[1] Clinical Trials Research Unit, Leeds Institute of Clinical Trials Research, University of Leeds, Leeds, UK, LS2 9JT; [2] St John's Institute of Dermatology, Guy's & St Thomas's NHS Foundation Trust, London, UK; [3] Dermatology Centre, Salford Royal NHS Foundation Trust, Manchester, UK; [4] Street Lane Practice, Roundhay, Leeds, LS8 1AY; [5]Department of Dermatology, Ninewells Hospital, Dundee, Tayside, DD1 9SY; [6] Department of Dermatology, Chapel Allerton Hospital, Leeds Teaching Hospitals NHS Trust, Leeds, LS7 4SA; [7] Faculty of Health Education and Life Sciences, Birmingham City University, Birmingham, UK; [8]Department of Dermatology, Newcastle Hospitals, Newcastle, Tyne and Wear, UK; [9] Faculty of Biological Sciences, University of Leeds, Leeds, UK; [10] Academic Unit of Health Economics, Leeds Institute of Health Sciences, University of Leeds, Leeds, UK, LS2 9JT; [11] Institute of Health and Society, Faculté de Santé Publique, Clos Chapelle-aux-Champs, 30, Box B1.30.01 B-1200 Brussels; [12] Institute of Economic and Social Research (IRES) – LIDAM, Collège L. H. Dupriez, 3 Place Montesquieu, Box L2.06.01 B- 1348 Louvain-la-Neuve ; [13] Leeds Institute of Rheumatic and Musculoskeletal Medicine, University of Leeds, Leeds, Yorkshire, UK; [14] NIHR Leeds Musculoskeletal Biomedical Research Unit, Chapel Allerton Hospital, Leeds, Yorkshire, UK; [15] Department of Dermatology, Bradford Teaching Hospitals NHS Foundation Trust, Bradford, Yorkshire, UK

Word count (excluding title page, abstract, references, figures and tables): 4908

Protocol version 9.0, dated 20/09/2019

Keywords: Dermatology, Eczema, Photodermatology, Occupational Dermatology, Allergy

ABSTRACT

Introduction

Hand eczema (HE) is one of the most common skin disorders and an important cause for morbidity and occupational disability. The 1-year prevalence of HE is estimated to be up to 10% and it is estimated that 5-7% of all patients with HE develop severe chronic HE (CHE). However, current clinical evidence is not compelling enough to guide clinical practice. In a survey among 194 UK dermatologists the most frequent first choice approaches were PUVA, oral steroids and alitretinoin

For peer review only

(AL). When asked which strategy was most efficient for long-term outcome 20% of clinicians indicated they did not know; 43% of clinicians reported AL and 30% reported PUVA.

Methods and analysis

ALPHA is a multicentre, open, prospective, two arm parallel group, randomised controlled trial comparing PUVA and AL with a planned sample size re-estimation. Between 500 and 780 participants will be randomised on a 1:1 basis to AL or PUVA. The physician's global assessment (PGA) will direct treatment after randomisation, non-responders will be treated according to usual clinical practice; providing valuable pilot data on second line therapeutic approaches to inform future trials.

Assessments will be conducted up to 52 weeks post randomisation. The primary outcome measure is the Hand Eczema Severity Index (HECSI) at 12 weeks. Secondary outcome measures include modified Total Lesion Symptom Score (mTLSS), PGA, time to relapse, patient reported outcome measures and DNA extraction and assessment of genetic variants. A sub-study on molecular inflammatory mediators will provide information on subgroup specific treatment responses. Photographs will be taken and HE severity assessed by a central review panel.

Ethics and dissemination

Ethics approval was obtained from Leeds West Research Ethics Committee (14/YH/1259).Trial results will be disseminated at relevant clinical conferences and societies, published in peer-reviewed journals and through relevant patient groups.

Trial registration

ALPHA is registered with ISRCTN (ISRCTN80206075)

For peer review only

For peer review only - http://bmjopen.bmj.com/site/about/guidelines.xhtml

STRENGTHS AND LIMITATIONS OF THIS STUDY

Strengths

- The trial will directly compare treatment approaches used in clinical practice as first line treatment for severe hand eczema, providing valuable data on their effectiveness which are not available at present.
- ALPHA takes disease subtypes and a range of factors into account known to influence response to treatment and will close an important knowledge gap on how to best treat patients with different morphologies, disease duration, allergy background and skin barrier composition with regard to filaggrin.
- This is the first interventional trial on severe hand eczema which will compare different and previously used outcome measures for the assessment of severity meaning it will become possible to compare previous/other trials which have used different outcome measures.

Limitations

- Clinical assessment of HE severity was completed by a blinded assessor, as delivery of PUVA and Alitretinoin could not be blinded, and we will consider inter-observer variations in assessment in our analysis where the blinded assessor changed over time and within the follow up of the same patient (due to capacity issues).

For peer review only - http://bmjopen.bmj.com/site/about/guidelines.xhtml

For peer review only

INTRODUCTION

Background and rationale

Hand eczema (HE) is one of the most common skin disorders and an important cause of morbidity and occupational disability. It is estimated that up to 10% of the general population report HE at least once in a single year [1] and 5-7% of all patients with HE are estimated to develop severe chronic HE (CHE) [2].

The impact on daily life of sufferers is considerable [3, 4]. HE is characterised by severe itching and can be very painful and many studies have shown significant impairment of quality of life (QoL) [5, 6]. HE is a persistent disease with a relapsing course and variable disease duration; in some cases at least 15 years of continuous HE have been reported [6].

Uncertainty about the most effective treatment for HE is influenced by the fact that HE presents with several disease subgroups and underlying causes; and the natural course of HE often follows a recurrent pattern [2, 7]. The proposed classification in the UK [7] and Europe [8] defines atopic HE, allergic contact dermatitis and irritant dermatitis, either alone or in combination. However, discrimination of aetiologically distinct subtypes is a clinical challenge due to overlap/coexistence of different aetiologies [7].

Moreover, it can be difficult to distinguish HE from some types of psoriasis or other skin conditions even on a histopathology level [9, 10] if patients present without involvement of other body sites and if the condition has already been treated with topical corticosteroids. Clinicians are aware of “mixed” phenotypes named eczema-in-psoriatico or psoriasiform eczema [11]; which can be verified by skin biopsies, however it is not current practice to biopsy lesions in the UK. Samples obtained from the epidermis via tape stripping [12] or washing [13] for example, contain markers such as IL-36, TARC, CCL20, TSLP, and IL-18 which have potential to identify eczema subtypes subtypes on a molecular level and thereby to improve subtype diagnosis.

Loss-of-function mutations in filaggrin, a protein important for the skin barrier, have been shown to be associated with atopic eczema[14-18]. Experts have proposed [19] that HE classification should consider filaggrin genotype for better subgrouping and potentially targeted treatment.

Alitrenoin (9-cis retinoic acid) is a naturally occurring vitamin A derivative (retinoid) and is the only licensed systemic agent for severe, CHE unresponsive to treatment with potent topical corticosteroids (NICE TA177). However, there is a lack of controlled clinical trials that directly compare Alitretinoin to other treatments as a first line therapy to demonstrate clinical effectiveness under daily practice conditions, which has been acknowledged by national and international expert groups [20].

For peer review only - http://bmjopen.bmj.com/site/about/guidelines.xhtml

Our choice of comparator for Alitretinoin was based on published clinical trials [8], and feedback from UK dermatologists, patients and the UK Dermatology Clinical Trials Network (UKDCTN). A survey conducted among 194 UK dermatologists informed the choice of immersion psoralen combined with ultraviolet A (PUVA) as the comparator treatment with both PUVA and Alitretinion identified as popular first line therapies for severe hyperkeratotic and vesicular CHE [21].

PUVA is used extensively across the NHS and comprises a photosensitising agent in combination with UV-A. It is effective in both vesicular and hyperkeratotic hand eczema [22]. The photosensitising agent 8-MOP (methoxsalen, 8-methoxypsoralen) is used and the most common route of administration for this compound in the treatment of HE is topical (e.g. gel, cream, immersion) [23, 24].

The importance of topical PUVA was highlighted in a 'consensus statement' on the treatment of CHE [7] as a widely used management option, although this is based more on clinical experience than on evidence.

Given the high socioeconomic impact of the disease and the pressing need for comparative studies on available first line treatments, ALPHA is a randomised controlled trial (RCT) comparing immersion PUVA and Alitretinoin for the treatment of severe CHE.

METHODS AND DESIGN

Objectives

The overall aim of the trial is to determine the clinical and cost effectiveness of Alitretinoin and immersion PUVA when used in conjunction with concomitant topical corticosteroids, emollients and patient education for the treatment of severe CHE which is unresponsive to treatment with potent topical corticosteroids alone.

The primary objective is to compare Alitretinoin and immersion PUVA as first line therapy in terms of disease activity at 12 weeks post planned start of treatment.

The secondary objectives are to compare Alitretinoin to PUVA over 52 weeks post planned start of treatment in terms of:

1. Disease activity over time
2. Time to relapse
3. Quality of life (QoL) and patient benefit over time
4. Within trial and long term cost effectiveness
5. Safety
6. Educational need for patients.

For peer review only - http://bmjopen.bmj.com/site/about/guidelines.xhtml

Exploratory objectives are to:

1. Compare scoring systems HECSI, mTLSS, DLQI and PGA used to monitor treatment response
2. Explore whether response to first line treatment is affected by: duration of disease, clinical phenotype, disease severity, presence of atopy, smoking history. BMI, foot involvement and Filaggrin loss of function mutation and other potential emerging mutations affecting skin barrier or response to treatment.
3. Collect pilot data on clinical effectiveness of second line therapies.
4. Explore treatment responses in hand eczema (HE) subgroups defined by molecular inflammatory mediators .
5. Compare Alitretinoin and PUVA in terms of time in remission
6. Compare Alitretinoin and PUVA in terms of nail assessment
7. Explore the use of the photography guide for patients from minority ethnic groups.

Trial design

The ALPHA trial is a multicentre, prospective, open-label, two-arm parallel group, adaptive, RCT with one planned interim analysis.

A maximum of 780 consenting participants with severe CHE will be randomised on a 1:1 basis to receive either Alitretinoin or PUVA in addition to concomitant topical corticosteroids, emollients and patient education. The trial is an adaptive design with a planned interim analysis to re-estimate the final sample size.

An internal pilot phase will inform on the feasibility of recruitment and delivery of the trial.

METHODS: PARTICIPANTS, INTERVENTIONS AND OUTCOMES

Trial setting and recruitment

Patients will be screened in UK secondary care dermatology outpatient, community hospital and General Practice settings. Patients complete a self-screening questionnaire on the trial website and if eligible, will be invited to a formal eligibility assessment at one of the participating research sites which will take on the responsibility for seeking consent and undertaking trial research procedures. Formal eligibility assessment and recruitment will be undertaken in secondary care dermatology outpatient clinics.

Eligibility criteria

Patients suffering with severe CHE and unresponsive to at least 4 weeks of treatment with potent topical corticosteroids will be assessed for eligibility in accordance with the criteria in Table 1.

For peer review only - http://bmjopen.bmj.com/site/about/guidelines.xhtml

For peer review only

Table 1 Eligibility Criteria

Inclusion Criteria	Exclusion Criteria
1. Aged ≥18 years at the time of signing the Informed Consent Form<br>2. Suffering from uncontrolled, severe CHE defined as the presence of *both* of the following criteria:<br>a) PGA score of severe [25]<br>b) Resistance to treatment with potent topical corticosteroids for ≥ 4 weeks prior to the point of eligibility screening.<br>3. Provided written informed consent.<br>4. Expected to comply with treatment and protocol schedule.	***Skin related:***<br>1. Patients who have a clinically suspected infection (fungal, bacterial or viral) as cause for dermatitis of the hands.<br>2. Patients with known clinically relevant allergic contact dermatitis of the hands unless they had made a reasonable effort to avoid the contact allergen.<br>3. Patients suffering from atopic eczema covering more than 10% of body surface (excluding hands).<br>4. Patients who have skin conditions worsened by the sun i.e. do not tolerate UV-light (for example lupus erythematosus, porphyria).<br>***Treatment related:***<br>5. Patients who have received phototherapy/photochemotherapy in the last 3 months prior to randomisation<br>6. Patients who have received systemic vitamin A derivatives or other systemic immunosuppressants e.g. methotrexate or biologics treatment for HE in the last 3 months prior to randomisation<br>7. Patients who have received Ciclosporin A or systemic glucocorticoid steroid treatment for HE within 1 week prior to randomisation.<br>8. Patients receiving topical calcineurin antagonist treatment within 1 week prior to randomisation.<br>9. Patients receiving concomitant treatment with tetracyclines, or medication with potential for drug-drug interaction with Alitretinoin (e.g. CYP3A4 inhibitor ketoconazole) that cannot be suspended or switched to an acceptable alternative<br>10. Patients receiving concomitant treatment with relevant photosensitisers, when this treatment cannot be suspended for the duration of the intervention or switched to an acceptable alternative<br>11. Patients with a history of melanoma skin cancer, or patients with a history of non-melanoma skin cancer depending on history, location and "severity" of the non-melanoma skin cancer based on experience from routine practice.<br>12. Patients who have received prior treatment with arsenic agents or ionising radiation in the treatment area (e.g. hands).<br>***General:***<br>13. If female:<br>a) Lactating<br>b) Of child bearing potential (WCBP):

For peer review only - http://bmjopen.bmj.com/site/about/guidelines.xhtml

	i. With positive pregnancy test (absence of pregnancy will be confirmed with a negative pregnancy test before randomisation)<br>ii. Unwilling to follow pregnancy prevention program measures* whilst receiving treatment and after the last dose of protocol treatment as indicated in the relevant SmPC<br>14. Patients with hepatic insufficiency (alanine aminotransferase and/or aspartate aminotransferase > 2.5 times the upper limit of normal), known severe renal insufficiency, uncontrolled hyperlipidaemia (for all of the following: triglycerides, cholesterol and/or LDL cholesterol) or uncontrolled hypothyroidism in the 12 week period prior to randomisation.<br>15. Patients with known hypersensitivity to peanut, soya or vitamin A derivatives or with rare hereditary fructose intolerance as determined by patient history.<br>16. Patients currently suffering from hypervitaminose A as directed by clinical symptoms or patient history<br>17. Patients previously participated in the ALPHA trial
**Rigorous contraception for WCBP, unless exempt according to standard of care practice, is required 1 month before treatment, during the treatment period and 1 month after cessation of treatment as per usual standard practice.*<br>*Concomitant treatments not permitted are provided in supplementary material 1*	

Participant timeline

Consent

Blood samples are required to confirm eligibility and atopy status (presence/absence of specific IgE) prior to randomisation, and research sites have the option of using a one stage process to obtain informed consent for the full trial, or a two stage consent process with separate consent for the eligibility blood sample taken up to 12 weeks prior to randomisation.,

Where eligibility is indicated, a full verbal explanation of the study and patient information leaflet will be provided by the attending clinical or research team. Patients will have as long as they need to consider participation and will be given the opportunity to discuss the trial with their family and other healthcare professionals before they are invited to take part.

For patients capable of providing consent but unable to sign or otherwise mark the consent from, witnessed consent will be provided by a carer, friend/family member or a local member of the clinical team independent of the research team.

Registration

All participants who consent will be registered (by an authorised member of staff at the trial research site) into the trial before any trial related procedures are performed, using the 24-hour automated registration telephone or web based system hosted by the Leeds CTRU.

For peer review only - http://bmjopen.bmj.com/site/about/guidelines.xhtml

Baseline Assessment and Randomisation

The baseline visit must be booked up to 7 days before the first PUVA appointment, within 12 weeks of the eligibility blood sample and after at least 1 month duration of the pregnancy prevention program (if applicable).

At the baseline visit, eligible patients will be invited to provide informed consent for the full trial, photography sub study and biomarker sub study (for selected centres).

Eligible patients who provide full informed consent and complete the baseline assessments will be randomised into the trial by an authorised member of the research team at the site using the 24-hour telephone or web based randomisation service hosted by the Leeds CTRU.

Allocation

Patients will be randomised in a 1:1 allocation ratio, to receive either Alitretinoin or immersion PUVA using a computer-generated minimisation programme incorporating a random element to ensure treatment groups are well balanced for the following participant characteristics: centre, diease duration (< 6 months / 6-24 months / >24 months), clinical phenotype (predominately hyperkeratotic / predominately vesicular/ fingertip dermatitis), presence of specific IgE, DLQI (<15, ≥15), ethnicity (white/fair/dark).

If the participant is randomised to receive Alitretinoin, the phototherapy department will be informed immediately that the PUVA treatment appointment will no longer be needed for this participant.

This randomisation service will also identify participants for participation in the biomarker and photography sub studies.

Interventions

In both trial arms education on HE in using emollients, avoiding irritants and relevant contact allergens will be delivered in a standardised way prior to randomisation. The information material for participants will be based on sources used in clinical practice (British Association of Dermatology (BAD), National Eczema Society, Eczema Society patient information leaflets).

Topical corticosteroids may be used as required, as a reflection of standard clinical practice, however it is recommended that they should belong to the "potent" group.

Alitretinoin

Participants randomised to Alitretinoin will be provided with a prescription dispensed as per standard care practice. It is anticipated that the patient will take the first dose of Alitretinoin within 7 days of randomisation.

Prescriptions of Alitretinoin can be written to allow up to 5 weeks supply of treatment, except people of child bearing potential following a pregnancy prevention program whereby Alitretinoin prescriptions should be limited to a 4 week supply.

For peer review only - http://bmjopen.bmj.com/site/about/guidelines.xhtml

For peer review only

According to standard clinical practice and NICE guidelines (TA177), Alitretinoin will be self-administered at a starting dose of 30mg, taken once daily with a meal for 12 weeks. After 12 weeks, participants will be assessed for their treatment response and depending on the outcome may continue Alitretinoin for up to 12 more weeks.

Dose adjustment down to 10mg or temporary cessation may occur according to standard practice in participants who suffer from related side effects such as headaches.

Immersion PUVA

Meladinine® (methoxsalen) will be used in combination with ultraviolet A (PUVA). Meladinine® 0.75% solution for local application in immersion PUVA is diluted to 3mg/L (prepared by mixing 0.8ml of 0.75% Meladinine® solution in 2L tap water). The participants hands are soaked for 15 min followed by a maximum 30 minute delay before UVA exposure. The UV-A radiation dose is individually tailored to the participant depending on phenotype (as per BAD guidelines [26]) and the erythematous response of the skin following treatment.

Calibration certificates for the UV-A machines are collected from centres prior to opening to ensure the same UV dose is administered across different centres.

Immersion PUVA will be administered twice weekly for 12 weeks in out-patient phototherapy departments and will be administered and supervised by specialised nurses/dermatologists according to local policy.

After 12 weeks of immersion PUVA treatment, participants will be assessed for their response to treatment and may continue immersion PUVA for up to 12 more weeks.

Treatment pathway: criteria of response

Responders, defined as a PGA score of clear/almost clear at 12 weeks post planned start of treatment, will discontinue randomised treatment.

Partial responders, defined as a PGA score of mild/moderate at 12 weeks post planned start of treatment, will continue with randomised treatment for up to a further 12 weeks. During this second 12 week treatment period patients will be monitored at 4 weekly intervals and randomised treatment can be stopped at any time if the participant responds, or symptoms worsen and in the opinion of the treating clinician there is no clinical benefit to continuation.

Non-responders, defined as a PGA score of severe at 12 weeks post planned start of treatment, will discontinue randomised treatment.

In line with standard clinical practice, cessation or alteration of regimens at any time will be at the discretion of attending clinicians or the participants themselves. All randomised treatment will be discontinued at the maximum 24 weeks treatment period and participants will continue to receive ‘standard clinical practice’ and follow-up monitoring until the end of the follow-up period.

For peer review only - http://bmjopen.bmj.com/site/about/guidelines.xhtml

Data collection

Clinical data and patient reported data will be collected at baseline, every 4 weeks to week 24, and 8 weekly thereafter to week 52. At each assessment a review of medication diaries will be conducted to obtain data on HE topical corticosteroid usage and any HE treatment received (in conjunction with clinical records review). Reportable adverse reactions will be collected and will exclude the following, providing they are non-serious, because they are known and common reactions; headaches and dry skin on regions of the body other than hands for Alitretinoin, and mild to moderate erythema or itching of skin in PUVA treated skin locations for the PUVA arm). For participants recruited from October 2019, the follow-up period ends at 24 weeks post planned start of treatment. A full schedule of assessments is provided in Supplementary Material 2.

Data will be monitored for quality and completeness by the CTRU. Missing data will be chased until received, confirmed as not available or the trial is at analysis. Data received will be linked anonymised and entered onto a secure database at CTRU in accordance with the 2018 Data Protection Act. Photographs taken will be transferred immediately by secure email to CTRU and immediately deleted from the camera once confirmation of receipt is received by the research team. The CTRU and Sponsor reserve the right to intermittently conduct source data verification exercises on a sample of participants, which will be carried out by staff from the CTRU or Sponsor.

Details of biological sample management is available in supplementary material 3.

Blinding

The trial is open-label as participants and investigators cannot be blinded to treatment allocation due to the nature of the PUVA intervention. However, the assessment of the HE severity scores will be undertaken by a clinical assessor (research/dermatology nurse or a clinician) who is blinded to the randomised treatment and where possible, will be the same person at each assessment of a participant. Participants will be reminded not to reveal which treatment they have received to the blinded assessors in order to preserve blinding.

Photographs will be taken for 20% randomly identified, consenting participants of white ethnicity. Photographs will be taken from all consenting participants of from minority ethnic groups at each centre. Photographs will be taken at baseline and 12 weeks post planned start of treatment. A blinded central review of the photographs will be conducted by a central review panel.

Outcomes

Primary outcome measure

The primary outcome is measured as the natural logarithm of the Hand Eczema Severity Index (HECSI) [27] at 12 weeks post planned start of treatment. The HECSI is a validated scoring system used to clinical assess the severity and extent of HE [27].

For peer review only - http://bmjopen.bmj.com/site/about/guidelines.xhtml

Secondary outcome measures

- The modified Total Lesion Symptom Score (mTLSS) [25]
- The Physician's Global Assessment (PGA) [25]
- Time to relapse defined as the time between achieving clear/almost clear overall on the PGA to scoring 75% of their baseline HECSI
- The Dermatology Life Quality Index (DLQI) [28]
- The Patient Benefit Index for Hand Eczema (PBI-HE) [29, 30]
- The Person Centered Dermatology Self-Care Index (PeDeSi) [31, 32]
- The EQ-5D-3L [33]
- A Health Resource Utilisation and Private Costs questionnaire
- Molecular inflammatory mediators (obtained from the tape stripping sample on participants selected for the biomarker sub study)
- Clinical observational descriptive assessments of the nails (singe centre only)
- Photographs assessed by a central review panel in line with the photographic guide [34].
- DNA extraction and assessment of genetic variants (including filaggrin loss-of-function genetic analysis), obtained from blood sample

Sample size

A minimum of 500 and maximum of 780 participants are required to detect a relative difference of 1.3 (clinical opinion) in HECSI score between treatment arms at 12 weeks post planned start of treatment (80% power; 2-sided 5% significance level) assuming a coefficient of variation (CV) between 1.175 and 1.7 and allowing for 20% attrition. A sample size review will be carried out after 364 participants (precision of -0.132 and +0.168 assuming CV=1.2) have reached 12 weeks post planned start of treatment, to revise the CV and the final sample size.

A sample of 100 consenting participants will be selected to take part in a biomarker sub study. Participants will be selected based on clinical phenotype and random treatment allocation to ensure 25 participants are selected from each combination of clinical phenotype (excluding fingertip dermatitis) and treatment group.

Statistical methods

A full statistical analysis plan (SAP) predefining all analyses and patient populations will be in place prior to any comparative analyses according to guidelines [35]. The analysis results will be reported according to CONSORT [36]. The amount and reason for missingness will be assessed by treatment group, and imputation of missing data may be considered.

Patient Populations

The primary analysis will be on an intention-to-treat (ITT) basis where participants will be analysed according to randomised treatment group. A per-protocol (PP) population will also be defined, which will include all eligible randomised participants who comply with their randomised treatment allocation excluding major protocol violators.

For peer review only - http://bmjopen.bmj.com/site/about/guidelines.xhtml

Interim analysis

After 364 participants have reached 12 weeks post planned start of treatment, the pooled estimate of the CV for the primary endpoint will be calculated and the sample size re-estimated. This will be conducted by an independent statistician. If the re-estimated sample size indicates that the study requires fewer than 500 participants, the final sample size will be 500 participants.

Primary endpoint analysis

Primary analysis

A multivariable multilevel repeated measures linear regression model will be fitted to the primary endpoint, $\log_e$(HECSI), adjusting for minimisation factors duration of disease, clinical phenotype, DLQI, presence of specific IgE to inhalant or other relevant allergens and ethnicity, and the covariates: smoking history, BMI, foot involvement [37, 38], filaggrin loss of function mutation, baseline $\log_e$(HECSI), and treatment group. Centre, participant and participant-time interaction will be fitted as random effects. The relative difference in the HECSI score at 12 weeks post planned start of treatment, corresponding 95% confidence intervals and p-values will be reported.

Secondary endpoint analyses

Multivariable multilevel repeated measures linear regression models of log (HECSI score), mTLSS, DLQI, PeDeSi and PBI-HE, and a multilevel repeated measures ordinal logistic regression model of the PGA over time will be fitted adjusting for the minimisation factors, covariates as for the primary endpoint analysis, corresponding baseline measurement, and treatment group, as fixed effects. Centre, participant and participant-time interaction will be fitted as random effects. The parameter estimates, corresponding 95% confidence intervals and p-values will be reported; contrasts for the treatment effect at 24 and 52 weeks post planned start of treatment will also be reported.
A Cox Proportional Hazards (PH) model (after confirming the PH assumption is valid) will be fitted to time to relapse adjusting for minimisation factors and covariates as for the primary endpoint analysis.

AEs and SAEs classified as related to treatment, hand eczema or resulting from administration of any research procedures will be reported descriptively.

Exploratory endpoints

The correlation between HECSI, mTLSS, DLQI and PGA will be calculated to assess convergent validity of scoring systems used to monitor response to treatment.

Subgroup analyses will be conducted to compare treatment effects within pre-defined sub-groups (duration of disease, clinical phenotype, disease severity, presence of atopy, filaggrin loss of function mutation, smoking history, BMI and foot involvement) and biomarkers (such as IL-36, TARC, CCL20, TSLP, and IL-18). Multivariable regression models will be fitted to the response, $\log_e$(HECSI), treatment group and an interaction term between treatment group and the subgroup/biomarker will be

For peer review only - http://bmjopen.bmj.com/site/about/guidelines.xhtml

included in the models to explore if there are potential differential treatment effects.

Types of, dose and, response to second line therapies will be presented descriptively using HECSI and PGA.

Definitions of the end of remission such as the time point that participants are no longer clear/almost clear will be explored in conjunction with definitions of relapse. Frequency of corticosteroid use and nail assessment data will be reported descriptively.

Agreement between the blinded assessor and the central review of photographs will be assessed using cross tabulations and kappa statistics. These will be presented by time point and by ethnicity. Where discrepancies exist, the mTLSS will be explored for further information relating to unobservable symptoms such as pain and itch.

Health economics analysis

A within trial and a long term cost-effectiveness model-based analysis will be undertaken. Both will consider a UK NHS and Personal Social Services perspective and a societal perspective, and use Quality Adjusted Life Year (QALYs) as the outcome measure. Healthcare utilisation collected as part of the follow-up will be combined with appropriate unit cost information. These will be added to the treatment costs. Societal costs will be calculated by adding healthcare costs to the costs of lost production, based on days off work combined with wage rates and other reported private costs. Utility weights for the QALYs calculation will be obtained from the EQ-5D3L. The within trial analysis will evaluate cost effectiveness at 52 weeks, while the long term analysis will be 10 years and costs and outcomes will be discounted at 3.5% per annum as recommended by NICE [37]. The effectiveness data from the trial will be synthesised with existing evidence where it exists. Parameter uncertainty in the within trial analysis will be assessed via a non-parametric bootstrap, while uncertainty in the long-term analysis will use probabilistic sensitivity analysis. The results of both analyses will be presented as expected incremental cost effectiveness ratios; cost effectiveness acceptability curves and expected net benefit, using the NICE threshold of £20,000 per QALY [37]. Secondary analyses will consider alternative time horizons and alternative utility values including the mapping of DLQI values to EQ-5D using algorithms already available [38, 39, 40].

Patient and Public Involvement

The trial grant application was supported by the Leeds Dermatology Patients Panel (LDPP), patient members of the UKDCTN, and the Leeds Musculoskeletal Biomedical Research Unit (LMBRU) patient and public advocacy group. Based on the feedback received, and in recognition to what is important to patients, we put a special emphasis on long term outcomes as well as on educational aspects in the trial design.

There are PPI representatives on the Trial Management Group and Trial Steering Committees who have provided input into the patient information sheet and other trial documentation. Both PPI

For peer review only - http://bmjopen.bmj.com/site/about/guidelines.xhtml

representatives also provide input into the design and conduct of the trial to ensure the patient perspective is fully integrated in key decisions about the trial, delivery and interpretation/dissemination of findings.

Ethics and dissemination

The trial was reviewed and approved by the Yorkshire and Humber Research Ethics Committee (REC reference: 14/YH/1259), the Health Research Authority (HRA) and Medicine and Healthcare products Regulatory Agency (MHRA).

Sites will be informed of any and all major changes to the trial protocol. Any significant amendments will be reported to the REC, HRA and MHRA.

Trial results will be disseminated at relevant conferences and published in peer-reviewed journals. Authorship will be decided according to ICMJE guidelines as to qualifying contributions.

Methods: Monitoring

Trial governance

An independent Data Monitoring and Ethics Committee (DMEC) consisting 1 statistician and 2 clinicians will meet at least annually to review the safety and ethics of the study and the results of the interim analysis. The independent Trial Steering Committee (TSC) consisting 1 statistician, 2 clinicians and 1 patient representative will meet 6 monthly and provide overall supervision of the trial, including trial progress, adherence to protocol, participant safety and consideration of new information. The TSC will also review the recommendations made by the DMEC following the sample size re-estimation and other reviews of data by treatment group.

Confidentiality

All information collected during the course of the trial will be kept strictly confidential. Information will be held securely on paper and electronically at the CTRU. The CTRU will comply with all aspects of the Data Protection Act 2018. Participants will remain free to withdraw at any time from the trial without giving reasons and without prejudicing his/her further treatment/care. If a participant withdraws consent from further trial treatment and/or further collection of data, their data will remain on file and will be included in the final trial analysis.

Trial Sponsor

The trial contact on behalf of the Sponsor is Clare Skinner, Faculty Head of Research Support, University of Leeds, Leeds LS2 9JT.

DISCUSSION

This trial will deliver information on the most effective treatment in patients with different hand eczema situations regarding – among others - morphology, disease duration, filaggrin mutations, atopy status and ethnicity.

For peer review only - http://bmjopen.bmj.com/site/about/guidelines.xhtml

Despite the fact that HE often presents as a severe disease which severely impacts on patients' working and social life there has been limited advancements regarding new treatments or identification of most effective treatments for a given patient. While this trial does not involve comparison of novel treatment strategies, it considers most known clinical factors which influence response to therapy. A main difficulty in the treatment of HE is the fact that disease subtypes are not clearly defined and/or recognisable in clinical reality. In addition outcome measures for assessment of severity are diverse and used inconsistently across different departments, countries and clinical trials. While ALPHA will not deliver an advanced precision medicine algorithm it will provide information on which treatment is best suited for patients with severe CHE, explore clinical subgroups and will also allow comparison of outcome measures.

In summary information collected within this trial will allow to close a significant knowledge gap regarding hand eczema treatment approaches and will certainly feed into treatment guidelines on this disease.

Trial Status

At the time of submission 441 participants have been randomised.

Data availability

The datasets during and/or analysed during the current study will be available from the corresponding author on reasonable request.

AUTHORS' CONTRIBUTIONS

ALPHA study design: IS, SB, CF, JN, CR, CS, JL, LS, CG, MG, FC, PH, AB, MW, ST. Writing of manuscript: IS, RG, CF, SB, JN, MW, AVP. All authors reviewed draft versions and approve of the final manuscript.

FUNDING STATEMENT

This study was funded by the National Institute for Health Research (NIHR) Health Technology Assessment (HTA) Programme (Project: 12/186/01). The views and opinions expressed therein are those of the authors and do not necessarily reflect those of the HTA, NIHR, NHS or the Department of Health.

COMPETING INTERESTS

None: MW, CG, SB, IS, AVP, LS, AB, PH, FC, CF, JN, CR, AB, CS, JL, MG,

ST is a Health economist expert on the NIHR PGfAR, the H2020 mental health in the workplace (2019), the H2020 Innovation in health technology appraisal (2018) and the Belgian FNRS. commission of doctoral FRIA scholarship

ACKNOWLEDGEMENTS

Steve Ersser was involved in the design of the study and trial protocol.

For peer review only - http://bmjopen.bmj.com/site/about/guidelines.xhtml

LICENCE STATEMENT

I, the Submitting Author has the right to grant and does grant on behalf of all authors of the Work (as defined in the below author licence), an exclusive licence and/or a non-exclusive licence for contributions from authors who are: i) UK Crown employees; ii) where BMJ has agreed a CC-BY licence shall apply, and/or iii) in accordance with the terms applicable for US Federal Government officers or employees acting as part of their official duties; on a worldwide, perpetual, irrevocable, royalty-free basis to BMJ Publishing Group Ltd ("BMJ") its licensees and where the relevant Journal is co-owned by BMJ to the co-owners of the Journal, to publish the Work in BMJ Open and any other BMJ products and to exploit all rights, as set out in our licence.

The Submitting Author accepts and understands that any supply made under these terms is made by BMJ to the Submitting Author unless you are acting as an employee on behalf of your employer or a postgraduate student of an affiliated institution which is paying any applicable article publishing charge ("APC") for Open Access articles. Where the Submitting Author wishes to make the Work available on an Open Access basis (and intends to pay the relevant APC), the terms of reuse of such Open Access shall be governed by a Creative Commons licence – details of these licences and which Creative Commons licence will apply to this Work are set out in our licence referred to above.

For peer review only

REFERENCES

1. Meding, B. and B. Jarvholm, *Hand eczema in Swedish adults - Changes in prevalence between 1983 and 1996.* Journal of Investigative Dermatology, 2002. **118**(4): p. 719-723.
2. Diepgen, T.L., et al., *Management of chronic hand eczema.* Contact Dermatitis, 2007. **57**(4): p. 203-210.
3. Cvetkovski, R.S., et al., *Relation between diagnoses on severity, sick leave and loss of job among patients with occupational hand eczema.* British Journal of Dermatology, 2005. **152**(1): p. 93-98.
4. Fowler, J.E., et al., *Impact of chronic hand dermatitis on quality of life, work productivity, activity impairment, and medical costs.* Journal of the American Academy of Dermatology, 2006. **54**(3): p. 448-457.
5. Diepgen, T.L., et al., *Cost of illness from occupational hand eczema in Germany.* Contact Dermatitis, 2013. **69**(2): p. 99-106.
6. Meding, B., K. Wrangsjo, and B. Jarvholm, *Fifteen-year follow-up of hand eczema: persistence and consequences.* British Journal of Dermatology, 2005. **152**(5): p. 975-980.
7. English, J., et al., *Consensus statement on the management of chronic hand eczema.* Clinical and Experimental Dermatology, 2009. **34**(7): p. 761-769.
8. Diepgen, T.L., et al., *Hand eczema classification: a cross-sectional, multicentre study of the aetiology and morphology of hand eczema.* British Journal of Dermatology, 2009. **160**(2): p. 353-358.
9. Politiek, K., et al., *Hyperkeratotic hand eczema: Eczema or not?* 2020. **83**(3): p. 196-205.
10. Park, J.Y., et al., *The histopathological differentiation between palmar psoriasis and hand eczema: a retrospective review of 96 cases.* 2017. **77**(1): p. 130-135.
11. Kolesnik, M., et al., *Eczema in Psoriatico: An Important Differential Diagnosis Between Chronic Allergic Contact Dermatitis and Psoriasis in Palmoplantar Localization.* 2018. **98**(1).
12. Benson, N.R., et al., *An analysis of select pathogenic messages in lesional and non-lesional psoriatic skin using non-invasive tape harvesting.* Journal of Investigative Dermatology, 2006. **126**(10): p. 2234-2241.
13. Wittersheim, M., et al., *Differential expression and in vivo secretion of the antimicrobial peptides psoriasin (S100A7), RNase 7, human beta-defensin-2 and-3 in healthy human skin.* Experimental Dermatology, 2013. **22**(5): p. 364-366.
14. Molin, S., et al., *Filaggrin mutations may confer susceptibility to chronic hand eczema characterized by combined allergic and irritant contact dermatitis.* British Journal of Dermatology, 2009. **161**(4): p. 801-807.
15. Thyssen, J.P., *The association between filaggrin mutations, hand eczema and contact dermatitis: a clear picture is emerging.* British Journal of Dermatology, 2012. **167**(6): p. 1197-1198.
16. Thyssen, J.P., *Atopic dermatitis, filaggrin mutations and irritant contact dermatitis.* British Journal of Dermatology, 2013. **168**(2): p. 233-234.

For peer review only

17. Thyssen, J.P., et al., *Filaggrin null mutations increase the risk and persistence of hand eczema in subjects with atopic dermatitis: results from a general population study.* British Journal of Dermatology, 2010. **163**(1): p. 115-120.
18. Thyssen, J.P., et al., *Filaggrin mutations are strongly associated with contact sensitization in individuals with dermatitis.* Contact Dermatitis, 2013. **68**(5): p. 273-276.
19. Giwercman, C., et al., *Classification of atopic hand eczema and the filaggrin mutations.* Contact Dermatitis, 2008. **59**(5): p. 257-260.
20. Paulden, M., et al., *Alitretinoin for the treatment of severe chronic hand eczema.* Health Technology Assessment, 2010. **14**: p. 39-46.
21. Smith, I.L., et al., *Treatment of severe, chronic hand eczema: results from a UK-wide survey.* Clin Exp Dermatol, 2016.
22. Schempp, C.M., et al., *Treatment of chronic palmoplantar eczema with local bath-PUVA therapy.* Journal of the American Academy of Dermatology, 1997. **36**(5): p. 733-737.
23. Coenraads, P.J., *Hand Eczema.* New England Journal of Medicine, 2012. **367**(19): p. 1829-1837.
24. Diepgen, T.L., et al., *Guideline on the Management of Hand Eczema ICD-10 Code: L20. L23. L24. L25. L30.* JDDG: Journal der Deutschen Dermatologischen Gesellschaft, 2009. **7**(s3).
25. Ruzicka, T., et al., *Efficacy and safety of oral alitretinoin (9-cis retinoic acid) in patients with severe chronic hand eczema refractory to topical corticosteroids: results of a randomized, double-blind, placebo-controlled, multicentre trial.* British Journal of Dermatology, 2008. **158**(4): p. 808-817.
26. Ling, T.C., et al., *British Association of Dermatologists and British Photodermatology Group guidelines for the safe and effective use of psoralen-ultraviolet A therapy 2015.* British Journal of Dermatology, 2016. **174**(1): p. 24-55.
27. Held, E., et al., *The hand eczema severity index (HECSI): a scoring system for clinical assessment of hand eczema. A study of inter- and intraobserver reliability.* British Journal of Dermatology, 2005. **152**(2): p. 302-307.
28. Basra, M.K.A., et al., *The Dermatology Life Quality Index 1994-2007: a comprehensive review of validation data and clinical results.* British Journal of Dermatology, 2008. **159**(5): p. 997-1035.
29. Augustin, M., et al., *The patient benefit index: a novel approach in patient-defined outcomes measurement for skin diseases.* Archives of Dermatological Research, 2009. **301**(8): p. 561-571.
30. Blome, C., et al., *Measurement of patient-relevant benefits in the treatment of chronic hand eczema - a novel approach.* Contact Dermatitis, 2009. **61**(1): p. 39-45.
31. Cowdell, F., Ersser, S., Gradwell, C., Langford, A., *The development, validity, reliability and field testing of the Person- Centred Dermatology Self-Care Index.* Archives of Dermatological Research, 2012. **148**: p. 1251-1256.

For peer review only - http://bmjopen.bmj.com/site/about/guidelines.xhtml

32. Cowdell, F., et al., *The Person-Centered Dermatology Self-Care Index A Tool to Measure Education and Support Needs of Patients With Long-term Skin Conditions.* Archives of Dermatology, 2012. **148**(11): p. 1251-1255.

33. EuroQol, G., *EuroQol--a new facility for the measurement of health-related quality of life.* Health Policy, 1990. **16**(3): p. 199-208.

34. Coenraads, P.J., et al., *Construction and validation of a photographic guide for assessing severity of chronic hand dermatitis.* British Journal of Dermatology, 2005. **152**(2): p. 296-301.

35. Gamble, C., et al., *Guidelines for the content of statistical analysis plans in clinical trials.* 2017. **318**(23): p. 2337-2343.

36. Moher, D., et al., *The CONSORT statement: revised recommendations for improving the quality of reports of parallel-group randomized trials.* 2001. **285**(15): p. 1987-1991.

37. NICE. (2013). *Guide to the methods of technology appraisal 2013*. Retrieved November 20, 2018 from https://www.nice.org.uk/process/pmg9/resources/guide-to-the-methods-of-technology-appraisal-2013-pdf-2007975843781

38. Vilsbøll, A.W., Kragh, N., Hahn-Pedersen, J. *et al.* Mapping Dermatology Life Quality Index (DLQI) scores to EQ-5D utility scores using data of patients with atopic dermatitis from the National Health and Wellness Study. *Qual Life Res* **29,** 2529–2539 (2020)

39. Niall J. Davison., et al., *Generating EQ-5D-3L Utility Scores from the Dermatology Life Quality Index: A Mapping Study in Patients with Psoriasis*. Value in Health 2018 **21**(8) 2018, p1010-1018

40. Ali FM, Kay R, Finlay AY, et al. Mapping of the DLQI scores to EQ-5D utility values using ordinal logistic regression. *Qual Life Res*. 2017;26(11):3025-3034. doi:10.1007/s11136-017-1607-4

For peer review only

SUPPLEMENTARY MATERIAL 2 - CONCOMITANT TREATMENTS

Concomitant treatments which are NOT permitted during the interventional phase of the trial are detailed below;

For all participants:

- Topical calcineurin antagonists
- New systemic corticosteroids for reasons other than hand eczema

For participants randomised to PUVA:

- Medication that may act as significant photosensitisers (e.g. tetracycline antibiotics) according to phototherapy guidelines.

For participants randomised to Alitretinoin:

- Systemic tetracycline antibiotics
- Other vitamin A derivatives.
- Other drugs with potential for drug-drug interaction (e.g. CYP3A4 inhibitor ketoconazole).

For peer review only

SUPPLEMENTARY MATERIAL 1

Table 2 ALPHA Trial Assessment Schedule

Weeks		0	4	8	12	16	20	24	28	32	36	44	52
Trial period	Screening	Baseline	Interventional			Interventional/ Follow-up			Follow-up				
Informed Consent (Full/Shortened/Optional photography)	X	X											
Registration	X												
Eligibility blood sample & IgE tests	X												
Start contraception prevention program (if applicable)	X	X											
Eligibility assessment		X											
Medical history, Clinical assessment, Gene Variant analysis blood sample		X											
PeDeSI by treating clinician/unblinded nurse		X			X								X
PGA by treating clinician		X	X	X	X	X	X	X	X	X	X	X	X
PGA, HECSI by blinded assessor		X	X	X	X	X	X	X	X	X	X	X	X
mTLSS by blinded assessor		X			X			X			X		X
Nail assessment by blinded assessor (Bradford recruited participants only)		X	X	X	X	X	X	X	X	X	X	X	X
DLQI		X	X	X	X	X	X	X	X	X	X	X	X
PBI-HE, EQ-5D-3L		X			X			X			X		X
Health resource utilisation questionnaire					X			X			X		X
Provide standard education for HE		X											
Randomisation		X											
Tape stripping sample collection (for randomly selected participants only)		X											
Photograph hands		X			X								
Randomised treatment compliance (medication diary review)			X	X	X	X	X	X					
Topical corticosteroid usage (medication diary review)			X	X	X	X	X	X	X	X	X	X	X
Details of treatment under 'standard clinical practice'						X	X	X	X	X	X	X	X
Reportable Adverse reactions and Related SAEs/SARs/SUSARs			X	X	X	X	X	X	X	X	X	X	X

For peer review only

SUPPLEMENTARY MATERIAL 3 – HANDLING OF BIOLOGICAL SAMPLES

Consent

Informed, written consent for the blood sample/s must be obtained prior to registration and prior to the participant undergoing procedures that are specifically for the purposes of the trial and are not standard routine care at the participating sites.

Gene variant analysis blood sample

A blood sample is taken at baseline (although also possible at any later visit) in order to obtain DNA for subsequent analysis for the filaggrin mutation and other skin barrier molecule polymorphisms. These samples will be anonymised and sent to Professor Ann Morgan's Molecular Rheumatology laboratory (Leeds Institute Cardiovascular and Metabolic Medicine, The LIGHT Laboratories, Clarendon Way, Leeds) for DNA extraction.

For blood samples that have been damaged or classed as unusable during transit to the laboratory, the CTRU will contact research sites to request a replacement sample is obtained, if possible, from the participant at the next trial visit.

A proportion of extracted DNA will be sent to Professor Stephan Weidinger's laboratory (Department of Dermatology, University Hospital Schleswig-Holstein, Kiel, Germany) for assessment of skin barrier molecule polymorphisms (including filaggrin loss-of-function mutation). The remainder of the extracted DNA will be placed in long term storage (under suitable conditions) in the laboratory of Anne Morgan for potential future research. For example, there is evidence linking PUVA treatment and mutations in the vitamin D receptor and this sample set will provide an invaluable resource to delineate any causal link.

Biomarker sub study

A subset of 100 participants recruited from selected centres will be asked to provide written informed consent to this optional sub study to consent to the following procedures, which will be collected prior to the start of the randomised treatment:

- **Tape stripping technique** involves an adhesive to bind and remove the top epidermal layers. This approach has been used as a non-invasive technique for direct sampling of skin [17, 18]. Tape stripping is generally rapid, and patient-friendly. For detailed instructions please refer to the biomarker sub study work instruction. Tape strips will be submersed in buffer solution and stored at site at -80ºC prior to sending to Dr Miriam Wittmann's laboratory (Section of Musculoskeletal Disease, Leeds Institute of Molecular Medicine, Wellcome Trust Brenner Building, St. James's University Hospital, Leeds).

- **Skin washing:** Where tape stripping is not possible due to acute inflammation, mediator content can be measured in skin washing fluids [47]. For detailed instructions please refer to the biomarker sub study work instruction. Buffer scrubs (washing fluid) will be stored at site at -80ºC prior to sending to Dr Miriam Wittmann's laboratory (Section of Musculoskeletal Disease, Leeds Institute of Molecular Medicine, Wellcome Trust Brenner Building, St. James's University Hospital, Leeds, UK)

For peer review only

SPIRIT
STANDARD PROTOCOL ITEMS: RECOMMENDATIONS FOR INTERVENTIONAL TRIALS

SPIRIT 2013 Checklist: Recommended items to address in a clinical trial protocol and related documents*

Section/item	ItemNo	Description	Page
**Administrative information**			
Title	1	Descriptive title identifying the study design, population, interventions, and, if applicable, trial acronym	1, 2
Trial registration	2a	Trial identifier and registry name. If not yet registered, name of intended registry	2
	2b	All items from the World Health Organization Trial Registration Data Set	N/A
Protocol version	3	Date and version identifier	1
Funding	4	Sources and types of financial, material, and other support	16
Roles and responsibilities	5a	Names, affiliations, and roles of protocol contributors	1
	5b	Name and contact information for the trial sponsor	15
	5c	Role of study sponsor and funders, if any, in study design; collection, management, analysis, and interpretation of data; writing of the report; and the decision to submit the report for publication, including whether they will have ultimate authority over any of these activities	16
	5d	Composition, roles, and responsibilities of the coordinating centre, steering committee, endpoint adjudication committee, data management team, and other individuals or groups overseeing the trial, if applicable (see Item 21a for data monitoring committee)	

For peer review only

Introduction

Background and rationale	6a	Description of research question and justification for undertaking the trial, including summary of relevant studies (published and unpublished) examining benefits and harms for each intervention	4, 5
	6b	Explanation for choice of comparators	4, 5
Objectives	7	Specific objectives or hypotheses	5, 6
Trial design	8	Description of trial design including type of trial (eg, parallel group, crossover, factorial, single group), allocation ratio, and framework (eg, superiority, equivalence, noninferiority, exploratory)	6

Methods: Participants, interventions, and outcomes

Study setting	9	Description of study settings (eg, community clinic, academic hospital) and list of countries where data will be collected. Reference to where list of study sites can be obtained	6
Eligibility criteria	10	Inclusion and exclusion criteria for participants. If applicable, eligibility criteria for study centres and individuals who will perform the interventions (eg, surgeons, psychotherapists)	6, 7
Interventions	11a	Interventions for each group with sufficient detail to allow replication, including how and when they will be administered	9, 10
	11b	Criteria for discontinuing or modifying allocated interventions for a given trial participant (eg, drug dose change in response to harms, participant request, or improving/worsening disease)	10
	11c	Strategies to improve adherence to intervention protocols, and any procedures for monitoring adherence (eg, drug tablet return, laboratory tests)	11
	11d	Relevant concomitant care and interventions that are permitted or prohibited during the trial	7, 8 Supplementary material 1

For peer review only

Outcomes	12	Primary, secondary, and other outcomes, including the specific measurement variable (eg, systolic blood pressure), analysis metric (eg, change from baseline, final value, time to event), method of aggregation (eg, median, proportion), and time point for each outcome. Explanation of the clinical relevance of chosen efficacy and harm outcomes is strongly recommended	11, 12
Participant timeline	13	Time schedule of enrolment, interventions (including any run-ins and washouts), assessments, and visits for participants. A schematic diagram is highly recommended (see Figure)	8, 9 Supplementary material 2
Sample size	14	Estimated number of participants needed to achieve study objectives and how it was determined, including clinical and statistical assumptions supporting any sample size calculations	12
Recruitment	15	Strategies for achieving adequate participant enrolment to reach target sample size	6

Methods: Assignment of interventions (for controlled trials)

Allocation:

Sequence generation	16a	Method of generating the allocation sequence (eg, computer-generated random numbers), and list of any factors for stratification. To reduce predictability of a random sequence, details of any planned restriction (eg, blocking) should be provided in a separate document that is unavailable to those who enrol participants or assign interventions	9
Allocation concealment mechanism	16b	Mechanism of implementing the allocation sequence (eg, central telephone; sequentially numbered, opaque, sealed envelopes), describing any steps to conceal the sequence until interventions are assigned	9
Implementation	16c	Who will generate the allocation sequence, who will enrol participants, and who will assign participants to interventions	9

Blinding (masking)	17a	Who will be blinded after assignment to interventions (eg, trial participants, care providers, outcome assessors, data analysts), and how	11
	17b	If blinded, circumstances under which unblinding is permissible, and procedure for revealing a participant's allocated intervention during the trial	N/A
**Methods: Data collection, management, and analysis**			
Data collection methods	18a	Plans for assessment and collection of outcome, baseline, and other trial data, including any related processes to promote data quality (eg, duplicate measurements, training of assessors) and a description of study instruments (eg, questionnaires, laboratory tests) along with their reliability and validity, if known. Reference to where data collection forms can be found, if not in the protocol	11
	18b	Plans to promote participant retention and complete follow-up, including list of any outcome data to be collected for participants who discontinue or deviate from intervention protocols	11
Data management	19	Plans for data entry, coding, security, and storage, including any related processes to promote data quality (eg, double data entry; range checks for data values). Reference to where details of data management procedures can be found, if not in the protocol	11
Statistical methods	20a	Statistical methods for analysing primary and secondary outcomes. Reference to where other details of the statistical analysis plan can be found, if not in the protocol	12, 13, 14
	20b	Methods for any additional analyses (eg, subgroup and adjusted analyses)	13
	20c	Definition of analysis population relating to protocol non-adherence (eg, as randomised analysis), and any statistical methods to handle missing data (eg, multiple imputation)	12

Methods: Monitoring

Data monitoring	21a	Composition of data monitoring committee (DMC); summary of its role and reporting structure; statement of whether it is independent from the sponsor and competing interests; and reference to where further details about its charter can be found, if not in the protocol. Alternatively, an explanation of why a DMC is not needed	15
	21b	Description of any interim analyses and stopping guidelines, including who will have access to these interim results and make the final decision to terminate the trial	13
Harms	22	Plans for collecting, assessing, reporting, and managing solicited and spontaneously reported adverse events and other unintended effects of trial interventions or trial conduct	13
Auditing	23	Frequency and procedures for auditing trial conduct, if any, and whether the process will be independent from investigators and the sponsor	N/A

Ethics and dissemination

Research ethics approval	24	Plans for seeking research ethics committee/institutional review board (REC/IRB) approval	14
Protocol amendments	25	Plans for communicating important protocol modifications (eg, changes to eligibility criteria, outcomes, analyses) to relevant parties (eg, investigators, REC/IRBs, trial participants, trial registries, journals, regulators)	14
Consent or assent	26a	Who will obtain informed consent or assent from potential trial participants or authorised surrogates, and how (see Item 32)	8
	26b	Additional consent provisions for collection and use of participant data and biological specimens in ancillary studies, if applicable	9

For peer review only - http://bmjopen.bmj.com/site/about/guidelines.xhtml

Confidentiality	27	How personal information about potential and enrolled participants will be collected, shared, and maintained in order to protect confidentiality before, during, and after the trial	15
Declaration of interests	28	Financial and other competing interests for principal investigators for the overall trial and each study site	16
Access to data	29	Statement of who will have access to the final trial dataset, and disclosure of contractual agreements that limit such access for investigators	16
Ancillary and post-trial care	30	Provisions, if any, for ancillary and post-trial care, and for compensation to those who suffer harm from trial participation	N/A
Dissemination policy	31a	Plans for investigators and sponsor to communicate trial results to participants, healthcare professionals, the public, and other relevant groups (eg, via publication, reporting in results databases, or other data sharing arrangements), including any publication restrictions	2
	31b	Authorship eligibility guidelines and any intended use of professional writers	15
	31c	Plans, if any, for granting public access to the full protocol, participant-level dataset, and statistical code	16
**Appendices**			
Informed consent materials	32	Model consent form and other related documentation given to participants and authorised surrogates	N/A
Biological specimens	33	Plans for collection, laboratory evaluation, and storage of biological specimens for genetic or molecular analysis in the current trial and for future use in ancillary studies, if applicable	Supplementary material 3

*It is strongly recommended that this checklist be read in conjunction with the SPIRIT 2013 Explanation & Elaboration for important clarification on the items. Amendments to the protocol should be tracked and dated. The SPIRIT checklist is copyrighted by the SPIRIT Group under the Creative Commons "Attribution-NonCommercial-NoDerivs 3.0 Unported" license.

For peer review only - http://bmjopen.bmj.com/site/about/guidelines.xhtml

BMJ Open

Comparison of ALitretinoin with PUVA as the first line treatment in patients with severe chronic HAnd eczema (ALPHA): study protocol for a randomised controlled trial

Journal:	*BMJ Open*
Manuscript ID	bmjopen-2021-060029.R1
Article Type:	Protocol
Date Submitted by the Author:	16-Dec-2021
Complete List of Authors:	Smith, Isabelle; University of Leeds Clinical Trials Research Unit,<br>Gilberts, Rachael; University of Leeds, Clinical Trials Research Unit<br>Brown, Sarah; University of Leeds Clinical Trials Research Unit<br>Fernandez, Catherine; University of Leeds Clinical Trials Research Unit<br>Nixon, Jane; University of Leeds Clinical Trials Research Unit<br>Reynolds, Catherine; University of Leeds Clinical Trials Research Unit<br>Smith, C; St John's Institute of Dermatology<br>Lear, John; Salford Royal NHS Foundation Trust, Department of Dermatology<br>Sunderland, Lesley; Street Lane Medical Practice<br>Green, Cathy; Ninewells Hospital, Dermatology Department<br>Goodfield, Mark; Leeds Teaching Hospitals NHS Trust, Department of Dermatology<br>Cowdell, Fiona; University of Hull, Faculty of Health and Social Care<br>Hampton, Philip ; Newcastle Upon Tyne Hospitals NHS Trust, Department of Dermatology<br>Barker, Amy; University of Leeds, Faculty of Biological Sciences<br>Vargas-Palacios, Armando; University of Leeds Leeds Institute of Health Sciences,<br>Tubeuf, Sandy; Universite catholique de Louvain<br>Wittmann, Miriam; University of Leeds Faculty of Medicine and Health, Rheumatic and Musculoskeletal Medicine
**Primary Subject Heading**:	Dermatology
Secondary Subject Heading:	Immunology (including allergy), Occupational and environmental medicine
Keywords:	DERMATOLOGY, Eczema < DERMATOLOGY, Photodermatology < DERMATOLOGY, Occupational dermatology < DERMATOLOGY

SCHOLARONE™
Manuscripts

Comparison of ALitretinoin with PUVA as the first line treatment in patients with severe chronic HAnd eczema (ALPHA): study protocol for a randomised controlled trial

Isabelle L Smith*[1], Rachael Gilberts[1], Sarah Brown[1], Catherine Fernandez[1], Jane Nixon[1], Catherine Reynolds[1], Catherine Smith[2], John T Lear[3], Lesley Sunderland[4], Cathy Green[5], Mark Goodfield[6], Fiona Cowdell[7], Philip Hampton[8], Amy Barker[9], Armando Vargas-Palacios[10], Sandy Tubeuf[11,12], Miriam Wittmann[13,14,15,]

*corresponding author

Email Addresses

Isabelle L Smith: i.l.smith@leeds.ac.uk; Rachael Gilberts: R.M.Gilberts@leeds.ac.uk; Sarah Brown: medsbro@leed.ac.uk; Catherine Fernandez: c.fernandez@leeds.ac.uk; Jane Nixon: j.e.nixon@leeds.ac.uk ; Catherine Reynolds: c.reynolds@leeds.ac.uk; Catherine Smith: Catherine.smith@kcl.ac.uk; John Lear: john.lear@cmft.nhs.uk; Lesley Sunderland: LesleyS@nhs.net; Cathy Green: cathy.green@nhs.scot; Mark Goodfield: mark.goodfield@leedsth.nhs.uk; Fiona Cowdell: Fiona.Cowdell@bcu.ac.uk; Philip Hampton: philip.hampton@nhs.net; Amy Barker: a.m.barker@leeds.ac.uk; Armando Vargas-Palacios A.Vargas-Palacios@leeds.ac.uk; Sandy Tubeuf: sandy.tubeuf@uclouvain.be; Miriam Wittmann: m.wittmann@leeds.ac.uk

Affiliations

[1] Clinical Trials Research Unit, Leeds Institute of Clinical Trials Research, University of Leeds, Leeds, UK, LS2 9JT; [2] St John's Institute of Dermatology, Guy's & St Thomas's NHS Foundation Trust, London, UK; [3] Dermatology Centre, Salford Royal NHS Foundation Trust, Manchester, UK; [4] Street Lane Practice, Roundhay, Leeds, LS8 1AY; [5]Department of Dermatology, Ninewells Hospital, Dundee, Tayside, DD1 9SY; [6] Department of Dermatology, Chapel Allerton Hospital, Leeds Teaching Hospitals NHS Trust, Leeds, LS7 4SA; [7] Faculty of Health Education and Life Sciences, Birmingham City University, Birmingham, UK; [8]Department of Dermatology, Newcastle Hospitals, Newcastle, Tyne and Wear, UK; [9] Faculty of Biological Sciences, University of Leeds, Leeds, UK; [10] Academic Unit of Health Economics, Leeds Institute of Health Sciences, University of Leeds, Leeds, UK, LS2 9JT; [11] Institute of Health and Society, Faculté de Santé Publique, Clos Chapelle-aux-Champs, 30, Box B1.30.01 B-1200 Brussels; [12] Institute of Economic and Social Research (IRES) – LIDAM, Collège L. H. Dupriez, 3 Place Montesquieu, Box L2.06.01 B- 1348 Louvain-la-Neuve ; [13] Leeds Institute of Rheumatic and Musculoskeletal Medicine, University of Leeds, Leeds, Yorkshire, UK; [14] NIHR Leeds Musculoskeletal Biomedical Research Unit, Chapel Allerton Hospital, Leeds, Yorkshire, UK; [15] Department of Dermatology, Bradford Teaching Hospitals NHS Foundation Trust, Bradford, Yorkshire, UK

Word count (excluding title page, abstract, references, figures and tables): 4920

Protocol version 9.0, dated 20/09/2019

Keywords: Dermatology, Eczema, Photodermatology, Occupational Dermatology, Allergy

ABSTRACT

Introduction

Hand eczema (HE) is one of the most common skin disorders and an important cause for morbidity and occupational disability. The 1-year prevalence of HE is estimated to be up to 10% and it is estimated that 5-7% of those develop severe chronic HE (CHE). However, current clinical evidence is not compelling enough to guide clinical practice. In a survey among 194 UK dermatologists the most frequent first choice approaches were psoralen combined with ultraviolet A (UVA) treatment (PUVA),

For peer review only

oral steroids and alitretinoin (AL). When asked which strategy was most efficient for long-term outcome 20% of clinicians indicated they did not know; 43% of clinicians reported AL and 30% reported PUVA.

Methods and analysis

ALPHA is a multicentre, open, prospective, two arm parallel group, randomised controlled trial comparing PUVA and AL with a planned sample size re-estimation. Between 500 and 780 participants will be randomised on a 1:1 basis. The physician's global assessment (PGA) will direct treatment after randomisation, non-responders will be treated according to usual clinical practice; providing valuable pilot data on second line therapeutic approaches to inform future trials.

Assessments will be conducted up to 52 weeks post randomisation. The primary outcome measure is the Hand Eczema Severity Index (HECSI) at 12 weeks. Secondary outcome measures include modified Total Lesion Symptom Score (mTLSS), PGA, time to relapse, patient reported outcome measures and DNA extraction and assessment of genetic variants. A sub-study on molecular inflammatory mediators will provide information on subgroup specific treatment responses. Photographs will be taken and HE severity assessed by a central review panel.

Ethics and dissemination

Ethics approval was obtained from Leeds West Research Ethics Committee (14/YH/1259).Trial results will be disseminated at relevant clinical conferences and societies, published in peer-reviewed journals and through relevant patient groups.

Trial registration

ALPHA is registered with ISRCTN (ISRCTN80206075)

For peer review only

For peer review only - http://bmjopen.bmj.com/site/about/guidelines.xhtml

STRENGTHS AND LIMITATIONS OF THIS STUDY

Strengths

- The trial will directly compare treatment approaches used in clinical practice as first line treatment for severe hand eczema, providing valuable data on their effectiveness which are not available at present.
- ALPHA takes disease subtypes and a range of factors into account known to influence response to treatment and will close an important knowledge gap on how to best treat patients with different morphologies, disease duration, allergy background and skin barrier composition with regard to filaggrin.
- This is the first interventional trial on severe hand eczema which will compare different and previously used outcome measures for the assessment of severity meaning it will become possible to compare previous/other trials which have used different outcome measures.

Limitations

- Clinical assessment of HE severity was completed by a blinded assessor, as delivery of PUVA and Alitretinoin could not be blinded, and we will consider inter-observer variations in assessment in our analysis where the blinded assessor changed over time and within the follow up of the same patient (due to capacity issues).

For peer review only

For peer review only - http://bmjopen.bmj.com/site/about/guidelines.xhtml

INTRODUCTION

Background and rationale

Hand eczema (HE) is one of the most common skin disorders and an important cause of morbidity and occupational disability. It is estimated that up to 10% of the general population report HE at least once in a single year [1] and 5-7% of all patients with HE are estimated to develop severe chronic HE (CHE) [2].

The impact on daily life of sufferers is considerable [3, 4]. HE is characterised by severe itching and can be very painful and many studies have shown significant impairment of quality of life (QoL) [5, 6]. HE is a persistent disease with a relapsing course and variable disease duration; in some cases at least 15 years of continuous HE have been reported [6].

Uncertainty about the most effective treatment for HE is influenced by the fact that HE presents with several disease subgroups and underlying causes; and the natural course of HE often follows a recurrent pattern [2, 7]. The proposed classification in the UK [7] and Europe [8] defines atopic HE, allergic contact dermatitis and irritant dermatitis, either alone or in combination. However, discrimination of aetiologically distinct subtypes is a clinical challenge due to overlap/coexistence of different aetiologies [7].

Moreover, it can be difficult to distinguish HE from some types of psoriasis or other skin conditions even on a histopathology level [9, 10] if patients present without involvement of other body sites and if the condition has already been treated with topical corticosteroids. Clinicians are aware of "mixed" phenotypes named eczema-in-psoriatico or psoriasiform eczema [11]; which can be verified by skin biopsies, however it is not current practice to biopsy lesions in the UK. Samples obtained from the epidermis via tape stripping [12] or washing [13] for example, contain markers such as IL-36, TARC, CCL20, TSLP, and IL-18 which have potential to identify eczema subtypes subtypes on a molecular level and thereby to improve subtype diagnosis.

Loss-of-function mutations in filaggrin, a protein important for the skin barrier, have been shown to be associated with atopic eczema[14-18]. Experts have proposed [19] that HE classification should consider filaggrin genotype for better subgrouping and potentially targeted treatment.

Alitrenoin (9-cis retinoic acid) is a naturally occurring vitamin A derivative (retinoid) and is the only licensed systemic agent for severe, CHE unresponsive to treatment with potent topical corticosteroids (NICE TA177). However, there is a lack of controlled clinical trials that directly compare Alitretinoin to other treatments as a first line therapy to demonstrate clinical effectiveness under daily practice conditions, which has been acknowledged by national and international expert groups [20].

For peer review only - http://bmjopen.bmj.com/site/about/guidelines.xhtml

Our choice of comparator for Alitretinoin was based on published clinical trials [8], and feedback from UK dermatologists, patients and the UK Dermatology Clinical Trials Network (UKDCTN). A survey conducted among 194 UK dermatologists informed the choice of immersion psoralen combined with ultraviolet A (PUVA) as the comparator treatment with both PUVA and Alitretinion identified as popular first line therapies for severe hyperkeratotic and vesicular CHE [21].

PUVA is used extensively across the NHS and comprises a photosensitising agent in combination with UV-A. It is effective in both vesicular and hyperkeratotic hand eczema [22]. The photosensitising agent 8-MOP (methoxsalen, 8-methoxypsoralen) is used and the most common route of administration for this compound in the treatment of HE is topical (e.g. gel, cream, immersion) [23, 24].

The importance of topical PUVA was highlighted in a 'consensus statement' on the treatment of CHE [7] as a widely used management option, although this is based more on clinical experience than on evidence.

Given the high socioeconomic impact of the disease and the pressing need for comparative studies on available first line treatments, ALPHA is a randomised controlled trial (RCT) comparing immersion PUVA and Alitretinoin for the treatment of severe CHE.

METHODS AND DESIGN

Objectives

The overall aim of the trial is to determine the clinical and cost effectiveness of Alitretinoin and immersion PUVA when used in conjunction with concomitant topical corticosteroids, emollients and patient education for the treatment of severe CHE which is unresponsive to treatment with potent topical corticosteroids alone.

The primary objective is to compare Alitretinoin and immersion PUVA as first line therapy in terms of disease activity at 12 weeks post planned start of treatment.

The secondary objectives are to compare Alitretinoin to PUVA over 52 weeks post planned start of treatment in terms of:

1. Disease activity over time
2. Time to relapse
3. Quality of life (QoL) and patient benefit over time
4. Within trial and long term cost effectiveness
5. Safety
6. Educational need for patients.

For peer review only - http://bmjopen.bmj.com/site/about/guidelines.xhtml

Exploratory objectives are to:

1. Compare scoring systems HECSI, mTLSS, DLQI and PGA used to monitor treatment response
2. Explore whether response to first line treatment is affected by: duration of disease, clinical phenotype, disease severity, presence of atopy, smoking history. BMI, foot involvement and Filaggrin loss of function mutation and other potential emerging mutations affecting skin barrier or response to treatment.
3. Collect pilot data on clinical effectiveness of second line therapies.
4. Explore treatment responses in hand eczema (HE) subgroups defined by molecular inflammatory mediators .
5. Compare Alitretinoin and PUVA in terms of time in remission
6. Compare Alitretinoin and PUVA in terms of nail assessment
7. Explore the use of the photography guide for patients from minority ethnic groups.

Trial design

The ALPHA trial is a multicentre, prospective, open-label, two-arm parallel group, adaptive, RCT with one planned interim analysis.

A maximum of 780 consenting participants with severe CHE will be randomised on a 1:1 basis to receive either Alitretinoin or PUVA in addition to concomitant topical corticosteroids, emollients and patient education. The trial is an adaptive design with a planned interim analysis to re-estimate the final sample size.

An internal pilot phase will inform on the feasibility of recruitment and delivery of the trial.

METHODS: PARTICIPANTS, INTERVENTIONS AND OUTCOMES

Trial setting and recruitment

Patients will be screened in UK secondary care dermatology outpatient, community hospital and General Practice settings. Patients complete a self-screening questionnaire on the trial website and if eligible, will be invited to a formal eligibility assessment at one of the participating research sites which will take on the responsibility for seeking consent and undertaking trial research procedures. Formal eligibility assessment and recruitment will be undertaken in secondary care dermatology outpatient clinics.

Eligibility criteria

Patients suffering with severe CHE and unresponsive to at least 4 weeks of treatment with potent topical corticosteroids will be assessed for eligibility in accordance with the criteria in Table 1.

For peer review only - http://bmjopen.bmj.com/site/about/guidelines.xhtml

Table 1 Eligibility Criteria

**Inclusion Criteria**	**Exclusion Criteria**
1. Aged ≥18 years at the time of signing the Informed Consent Form<br>2. Suffering from uncontrolled, severe CHE defined as the presence of *both* of the following criteria:<br>a) PGA score of severe [25]<br>b) Resistance to treatment with potent topical corticosteroids for ≥ 4 weeks prior to the point of eligibility screening.<br>3. Provided written informed consent.<br>4. Expected to comply with treatment and protocol schedule.	***Skin related:***<br>1. Patients who have a clinically suspected infection (fungal, bacterial or viral) as cause for dermatitis of the hands.<br>2. Patients with known clinically relevant allergic contact dermatitis of the hands unless they had made a reasonable effort to avoid the contact allergen.<br>3. Patients suffering from atopic eczema covering more than 10% of body surface (excluding hands).<br>4. Patients who have skin conditions worsened by the sun i.e. do not tolerate UV-light (for example lupus erythematosus, porphyria).<br>***Treatment related:***<br>5. Patients who have received phototherapy/photochemotherapy in the last 3 months prior to randomisation<br>6. Patients who have received systemic vitamin A derivatives or other systemic immunosuppressants e.g. methotrexate or biologics treatment for HE in the last 3 months prior to randomisation<br>7. Patients who have received Ciclosporin A or systemic glucocorticoid steroid treatment for HE within 1 week prior to randomisation.<br>8. Patients receiving topical calcineurin antagonist treatment within 1 week prior to randomisation.<br>9. Patients receiving concomitant treatment with tetracyclines, or medication with potential for drug-drug interaction with Alitretinoin (e.g. CYP3A4 inhibitor ketoconazole) that cannot be suspended or switched to an acceptable alternative<br>10. Patients receiving concomitant treatment with relevant photosensitisers, when this treatment cannot be suspended for the duration of the intervention or switched to an acceptable alternative<br>11. Patients with a history of melanoma skin cancer, or patients with a history of non-melanoma skin cancer depending on history, location and “severity” of the non-melanoma skin cancer based on experience from routine practice.<br>12. Patients who have received prior treatment with arsenic agents or ionising radiation in the treatment area (e.g. hands).<br>***General:***<br>13. If female:<br>a) Lactating<br>b) Of child bearing potential (WCBP):

For peer review only - http://bmjopen.bmj.com/site/about/guidelines.xhtml

	i. With positive pregnancy test (absence of pregnancy will be confirmed with a negative pregnancy test before randomisation)<br>ii. Unwilling to follow pregnancy prevention program measures* whilst receiving treatment and after the last dose of protocol treatment as indicated in the relevant SmPC<br>14. Patients with hepatic insufficiency (alanine aminotransferase and/or aspartate aminotransferase > 2.5 times the upper limit of normal), known severe renal insufficiency, uncontrolled hyperlipidaemia (for all of the following: triglycerides, cholesterol and/or LDL cholesterol) or uncontrolled hypothyroidism in the 12 week period prior to randomisation.<br>15. Patients with known hypersensitivity to peanut, soya or vitamin A derivatives or with rare hereditary fructose intolerance as determined by patient history.<br>16. Patients currently suffering from hypervitaminose A as directed by clinical symptoms or patient history<br>17. Patients previously participated in the ALPHA trial
**Rigorous contraception for WCBP, unless exempt according to standard of care practice, is required 1 month before treatment, during the treatment period and 1 month after cessation of treatment as per usual standard practice.*<br>*Concomitant treatments not permitted are provided in supplementary material 1*	

Participant timeline

Consent

Blood samples are required to confirm eligibility and atopy status (presence/absence of specific IgE) prior to randomisation, and research sites have the option of using a one stage process to obtain informed consent for the full trial, or a two stage consent process with separate consent for the eligibility blood sample taken up to 12 weeks prior to randomisation.,

Where eligibility is indicated, a full verbal explanation of the study and patient information leaflet will be provided by the attending clinical or research team. Patients will have as long as they need to consider participation and will be given the opportunity to discuss the trial with their family and other healthcare professionals before they are invited to take part.

For patients capable of providing consent but unable to sign or otherwise mark the consent from, witnessed consent will be provided by a carer, friend/family member or a local member of the clinical team independent of the research team. A copy of the consent form is provided in supplementary material 2.

Registration

All participants who consent will be registered (by an authorised member of staff at the trial research site) into the trial before any trial related procedures are performed, using the 24-hour automated registration telephone or web based system hosted by the Leeds CTRU.

For peer review only - http://bmjopen.bmj.com/site/about/guidelines.xhtml

Baseline Assessment and Randomisation

The baseline visit must be booked up to 7 days before the first PUVA appointment, within 12 weeks of the eligibility blood sample and after at least 1 month duration of the pregnancy prevention program (if applicable).

At the baseline visit, eligible patients will be invited to provide informed consent for the full trial, photography sub study and biomarker sub study (for selected centres).

Eligible patients who provide full informed consent and complete the baseline assessments will be randomised into the trial by an authorised member of the research team at the site using the 24-hour telephone or web based randomisation service hosted by the Leeds CTRU.

Allocation

Patients will be randomised in a 1:1 allocation ratio, to receive either Alitretinoin or immersion PUVA using a computer-generated minimisation programme incorporating a random element to ensure treatment groups are well balanced for the following participant characteristics: centre, diease duration (< 6 months / 6-24 months / >24 months), clinical phenotype (predominately hyperkeratotic / predominately vesicular/ fingertip dermatitis), presence of specific IgE, DLQI (<15, ≥15), ethnicity (white/fair/dark).

If the participant is randomised to receive Alitretinoin, the phototherapy department will be informed immediately that the PUVA treatment appointment will no longer be needed for this participant.

This randomisation service will also identify participants for participation in the biomarker and photography sub studies.

Interventions

In both trial arms education on HE in using emollients, avoiding irritants and relevant contact allergens will be delivered in a standardised way prior to randomisation. The information material for participants will be based on sources used in clinical practice (British Association of Dermatology (BAD), National Eczema Society, Eczema Society patient information leaflets).

Topical corticosteroids may be used as required, as a reflection of standard clinical practice, however it is recommended that they should belong to the “potent” group.

Alitretinoin

Participants randomised to Alitretinoin will be provided with a prescription dispensed as per standard care practice. It is anticipated that the patient will take the first dose of Alitretinoin within 7 days of randomisation.

Prescriptions of Alitretinoin can be written to allow up to 5 weeks supply of treatment, except people of child bearing potential following a pregnancy prevention program whereby Alitretinoin prescriptions should be limited to a 4 week supply.

For peer review only - http://bmjopen.bmj.com/site/about/guidelines.xhtml

For peer review only

According to standard clinical practice and NICE guidelines (TA177), Alitretinoin will be self-administered at a starting dose of 30mg, taken once daily with a meal for 12 weeks. After 12 weeks, participants will be assessed for their treatment response and depending on the outcome may continue Alitretinoin for up to 12 more weeks.

Dose adjustment down to 10mg or temporary cessation may occur according to standard practice in participants who suffer from related side effects such as headaches.

Immersion PUVA

Meladinine® (methoxsalen) will be used in combination with ultraviolet A (PUVA). Meladinine® 0.75% solution for local application in immersion PUVA is diluted to 3mg/L (prepared by mixing 0.8ml of 0.75% Meladinine® solution in 2L tap water). The participants hands are soaked for 15 min followed by a maximum 30 minute delay before UVA exposure. The UV-A radiation dose is individually tailored to the participant depending on phenotype (as per BAD guidelines [26]) and the erythematous response of the skin following treatment.

Calibration certificates for the UV-A machines are collected from centres prior to opening to ensure the same UV dose is administered across different centres.

Immersion PUVA will be administered twice weekly for 12 weeks in out-patient phototherapy departments and will be administered and supervised by specialised nurses/dermatologists according to local policy.

After 12 weeks of immersion PUVA treatment, participants will be assessed for their response to treatment and may continue immersion PUVA for up to 12 more weeks.

Treatment pathway: criteria of response

Responders, defined as a PGA score of clear/almost clear at 12 weeks post planned start of treatment, will discontinue randomised treatment.

Partial responders, defined as a PGA score of mild/moderate at 12 weeks post planned start of treatment, will continue with randomised treatment for up to a further 12 weeks. During this second 12 week treatment period patients will be monitored at 4 weekly intervals and randomised treatment can be stopped at any time if the participant responds, or symptoms worsen and in the opinion of the treating clinician there is no clinical benefit to continuation.

Non-responders, defined as a PGA score of severe at 12 weeks post planned start of treatment, will discontinue randomised treatment.

In line with standard clinical practice, cessation or alteration of regimens at any time will be at the discretion of attending clinicians or the participants themselves. All randomised treatment will be discontinued at the maximum 24 weeks treatment period and participants will continue to receive 'standard clinical practice' and follow-up monitoring until the end of the follow-up period.

For peer review only - http://bmjopen.bmj.com/site/about/guidelines.xhtml

For peer review only

Data collection

Clinical data and patient reported data will be collected at baseline, every 4 weeks to week 24, and 8 weekly thereafter to week 52. At each assessment a review of medication diaries will be conducted to obtain data on HE topical corticosteroid usage and any HE treatment received (in conjunction with clinical records review). Reportable adverse reactions will be collected and will exclude the following, providing they are non-serious, because they are known and common reactions; headaches and dry skin on regions of the body other than hands for Alitretinoin, and mild to moderate erythema or itching of skin in PUVA treated skin locations for the PUVA arm). For participants recruited from October 2019, the follow-up period ends at 24 weeks post planned start of treatment. A full schedule of assessments is provided in Supplementary Material 3.

Data will be monitored for quality and completeness by the CTRU. Missing data will be chased until received, confirmed as not available or the trial is at analysis. Data received will be linked anonymised and entered onto a secure database at CTRU in accordance with the 2018 Data Protection Act. Photographs taken will be transferred immediately by secure email to CTRU and immediately deleted from the camera once confirmation of receipt is received by the research team. The CTRU and Sponsor reserve the right to intermittently conduct source data verification exercises on a sample of participants, which will be carried out by staff from the CTRU or Sponsor.

Details of biological sample management is available in supplementary material 4.

Blinding

The trial is open-label as participants and investigators cannot be blinded to treatment allocation due to the nature of the PUVA intervention. However, the assessment of the HE severity scores will be undertaken by a clinical assessor (research/dermatology nurse or a clinician) who is blinded to the randomised treatment and where possible, will be the same person at each assessment of a participant. Participants will be reminded not to reveal which treatment they have received to the blinded assessors in order to preserve blinding.

Photographs will be taken for 20% randomly identified, consenting participants of white ethnicity. Photographs will be taken from all consenting participants of from minority ethnic groups at each centre. Photographs will be taken at baseline and 12 weeks post planned start of treatment. A blinded central review of the photographs will be conducted by a central review panel.

Outcomes

Primary outcome measure

The primary outcome is measured as the natural logarithm of the Hand Eczema Severity Index (HECSI) [27] at 12 weeks post planned start of treatment. The HECSI is a validated scoring system used to clinical assess the severity and extent of HE [27].

For peer review only - http://bmjopen.bmj.com/site/about/guidelines.xhtml

Secondary outcome measures

- The modified Total Lesion Symptom Score (mTLSS) [25]
- The Physician's Global Assessment (PGA) [25]
- Time to relapse defined as the time between achieving clear/almost clear overall on the PGA to scoring 75% of their baseline HECSI
- The Dermatology Life Quality Index (DLQI) [28]
- The Patient Benefit Index for Hand Eczema (PBI-HE) [29, 30]
- The Person Centered Dermatology Self-Care Index (PeDeSi) [31, 32]
- The EQ-5D-3L [33]
- A Health Resource Utilisation and Private Costs questionnaire
- Molecular inflammatory mediators (obtained from the tape stripping sample on participants selected for the biomarker sub study)
- Clinical observational descriptive assessments of the nails (singe centre only)
- Photographs assessed by a central review panel in line with the photographic guide [34].
- DNA extraction and assessment of genetic variants (including filaggrin loss-of-function genetic analysis), obtained from blood sample

Sample size

A minimum of 500 and maximum of 780 participants are required to detect a relative difference of 1.3 (clinical opinion) in HECSI score between treatment arms at 12 weeks post planned start of treatment (80% power; 2-sided 5% significance level) assuming a coefficient of variation (CV) between 1.175 and 1.7 and allowing for 20% attrition. A sample size review will be carried out after 364 participants (precision of -0.132 and +0.168 assuming CV=1.2) have reached 12 weeks post planned start of treatment, to revise the CV and the final sample size.

A sample of 100 consenting participants will be selected to take part in a biomarker sub study. Participants will be selected based on clinical phenotype and random treatment allocation to ensure 25 participants are selected from each combination of clinical phenotype (excluding fingertip dermatitis) and treatment group.

Statistical methods

A full statistical analysis plan (SAP) predefining all analyses and patient populations will be in place prior to any comparative analyses according to guidelines [35]. The analysis results will be reported according to CONSORT [36]. The amount and reason for missingness will be assessed by treatment group, and imputation of missing data may be considered.

Patient Populations

The primary analysis will be on an intention-to-treat (ITT) basis where participants will be analysed according to randomised treatment group. A per-protocol (PP) population will also be defined, which will include all eligible randomised participants who comply with their randomised treatment allocation excluding major protocol violators.

For peer review only - http://bmjopen.bmj.com/site/about/guidelines.xhtml

Interim analysis

After 364 participants have reached 12 weeks post planned start of treatment, the pooled estimate of the CV for the primary endpoint will be calculated and the sample size re-estimated. This will be conducted by an independent statistician. If the re-estimated sample size indicates that the study requires fewer than 500 participants, the final sample size will be 500 participants.

Primary endpoint analysis

Primary analysis

A multivariable multilevel repeated measures linear regression model will be fitted to the primary endpoint, log_e(HECSI), adjusting for minimisation factors duration of disease, clinical phenotype, DLQI, presence of specific IgE to inhalant or other relevant allergens and ethnicity, and the covariates: smoking history, BMI, foot involvement [37, 38], filaggrin loss of function mutation, baseline log_e(HECSI), and treatment group. Centre, participant and participant-time interaction will be fitted as random effects. The relative difference in the HECSI score at 12 weeks post planned start of treatment, corresponding 95% confidence intervals and p-values will be reported.

Secondary endpoint analyses

Multivariable multilevel repeated measures linear regression models of log (HECSI score), mTLSS, DLQI, PeDeSi and PBI-HE, and a multilevel repeated measures ordinal logistic regression model of the PGA over time will be fitted adjusting for the minimisation factors, covariates as for the primary endpoint analysis, corresponding baseline measurement, and treatment group, as fixed effects. Centre, participant and participant-time interaction will be fitted as random effects. The parameter estimates, corresponding 95% confidence intervals and p-values will be reported; contrasts for the treatment effect at 24 and 52 weeks post planned start of treatment will also be reported.
A Cox Proportional Hazards (PH) model (after confirming the PH assumption is valid) will be fitted to time to relapse adjusting for minimisation factors and covariates as for the primary endpoint analysis.

AEs and SAEs classified as related to treatment, hand eczema or resulting from administration of any research procedures will be reported descriptively.

Exploratory endpoints

The correlation between HECSI, mTLSS, DLQI and PGA will be calculated to assess convergent validity of scoring systems used to monitor response to treatment.

Subgroup analyses will be conducted to compare treatment effects within pre-defined sub-groups (duration of disease, clinical phenotype, disease severity, presence of atopy, filaggrin loss of function mutation, smoking history, BMI and foot involvement) and biomarkers (such as IL-36, TARC, CCL20, TSLP, and IL-18). Multivariable regression models will be fitted to the response, log_e(HECSI), treatment group and an interaction term between treatment group and the subgroup/biomarker will be

For peer review only - http://bmjopen.bmj.com/site/about/guidelines.xhtml

included in the models to explore if there are potential differential treatment effects.

Types of, dose and, response to second line therapies will be presented descriptively using HECSI and PGA.

Definitions of the end of remission such as the time point that participants are no longer clear/almost clear will be explored in conjunction with definitions of relapse. Frequency of corticosteroid use and nail assessment data will be reported descriptively.

Agreement between the blinded assessor and the central review of photographs will be assessed using cross tabulations and kappa statistics. These will be presented by time point and by ethnicity. Where discrepancies exist, the mTLSS will be explored for further information relating to unobservable symptoms such as pain and itch.

Health economics analysis

A within trial and a long term cost-effectiveness model-based analysis will be undertaken. Both will consider a UK NHS and Personal Social Services perspective and a societal perspective, and use Quality Adjusted Life Year (QALYs) as the outcome measure. Healthcare utilisation collected as part of the follow-up will be combined with appropriate unit cost information. These will be added to the treatment costs. Societal costs will be calculated by adding healthcare costs to the costs of lost production, based on days off work combined with wage rates and other reported private costs. Utility weights for the QALYs calculation will be obtained from the EQ-5D3L. The within trial analysis will evaluate cost effectiveness at 52 weeks, while the long term analysis will be 10 years and costs and outcomes will be discounted at 3.5% per annum as recommended by NICE [37]. The effectiveness data from the trial will be synthesised with existing evidence where it exists. Parameter uncertainty in the within trial analysis will be assessed via a non-parametric bootstrap, while uncertainty in the long-term analysis will use probabilistic sensitivity analysis. The results of both analyses will be presented as expected incremental cost effectiveness ratios; cost effectiveness acceptability curves and expected net benefit, using the NICE threshold of £20,000 per QALY [37]. Secondary analyses will consider alternative time horizons and alternative utility values including the mapping of DLQI values to EQ-5D using algorithms already available [38, 39, 40].

Patient and Public Involvement

The trial grant application was supported by the Leeds Dermatology Patients Panel (LDPP), patient members of the UKDCTN, and the Leeds Musculoskeletal Biomedical Research Unit (LMBRU) patient and public advocacy group. Based on the feedback received, and in recognition to what is important to patients, we put a special emphasis on long term outcomes as well as on educational aspects in the trial design.

There are PPI representatives on the Trial Management Group and Trial Steering Committees who have provided input into the patient information sheet and other trial documentation. Both PPI

For peer review only - http://bmjopen.bmj.com/site/about/guidelines.xhtml

representatives also provide input into the design and conduct of the trial to ensure the patient perspective is fully integrated in key decisions about the trial, delivery and interpretation/dissemination of findings.

Ethics and dissemination

The trial was reviewed and approved by the Yorkshire and Humber Research Ethics Committee (REC reference: 14/YH/1259), the Health Research Authority (HRA) and Medicine and Healthcare products Regulatory Agency (MHRA).

Sites will be informed of any and all major changes to the trial protocol. Any significant amendments will be reported to the REC, HRA and MHRA.

Trial results will be disseminated at relevant conferences and published in peer-reviewed journals. Authorship will be decided according to ICMJE guidelines as to qualifying contributions.

Methods: Monitoring

Trial governance

An independent Data Monitoring and Ethics Committee (DMEC) consisting 1 statistician and 2 clinicians will meet at least annually to review the safety and ethics of the study and the results of the interim analysis. The independent Trial Steering Committee (TSC) consisting 1 statistician, 2 clinicians and 1 patient representative will meet 6 monthly and provide overall supervision of the trial, including trial progress, adherence to protocol, participant safety and consideration of new information. The TSC will also review the recommendations made by the DMEC following the sample size re-estimation and other reviews of data by treatment group.

Confidentiality

All information collected during the course of the trial will be kept strictly confidential. Information will be held securely on paper and electronically at the CTRU. The CTRU will comply with all aspects of the Data Protection Act 2018. Participants will remain free to withdraw at any time from the trial without giving reasons and without prejudicing his/her further treatment/care. If a participant withdraws consent from further trial treatment and/or further collection of data, their data will remain on file and will be included in the final trial analysis.

Trial Sponsor

The trial contact on behalf of the Sponsor is Clare Skinner, Faculty Head of Research Support, University of Leeds, Leeds LS2 9JT.

DISCUSSION

This trial will deliver information on the most effective treatment in patients with different hand eczema situations regarding – among others - morphology, disease duration, filaggrin mutations, atopy status and ethnicity.

For peer review only - http://bmjopen.bmj.com/site/about/guidelines.xhtml

Despite the fact that HE often presents as a severe disease which severely impacts on patients' working and social life there has been limited advancements regarding new treatments or identification of most effective treatments for a given patient. While this trial does not involve comparison of novel treatment strategies, it considers most known clinical factors which influence response to therapy. A main difficulty in the treatment of HE is the fact that disease subtypes are not clearly defined and/or recognisable in clinical reality. In addition outcome measures for assessment of severity are diverse and used inconsistently across different departments, countries and clinical trials. While ALPHA will not deliver an advanced precision medicine algorithm it will provide information on which treatment is best suited for patients with severe CHE, explore clinical subgroups and will also allow comparison of outcome measures.

In summary information collected within this trial will allow to close a significant knowledge gap regarding hand eczema treatment approaches and will certainly feed into treatment guidelines on this disease.

Trial Status

At the time of submission 441 participants have been randomised.

Data availability

The datasets during and/or analysed during the current study will be available from the corresponding author on reasonable request.

AUTHORS' CONTRIBUTIONS

ALPHA study design: IS, SB, CF, JN, CR, CS, JL, LS, CG, MG, FC, PH, AB, MW, ST. Writing of manuscript: IS, RG, CF, SB, JN, MW, AVP. All authors reviewed draft versions and approve of the final manuscript.

FUNDING STATEMENT

This study was funded by the National Institute for Health Research (NIHR) Health Technology Assessment (HTA) Programme (Project: 12/186/01). The views and opinions expressed therein are those of the authors and do not necessarily reflect those of the HTA, NIHR, NHS or the Department of Health.

COMPETING INTERESTS

None: MW, CG, SB, IS, AVP, LS, AB, PH, FC, CF, JN, CR, AB, CS, JL, MG,

ST is a Health economist expert on the NIHR PGfAR, the H2020 mental health in the workplace (2019), the H2020 Innovation in health technology appraisal (2018) and the Belgian FNRS. commission of doctoral FRIA scholarship

ACKNOWLEDGEMENTS

Steve Ersser was involved in the design of the study and trial protocol.

For peer review only - http://bmjopen.bmj.com/site/about/guidelines.xhtml

LICENCE STATEMENT

I, the Submitting Author has the right to grant and does grant on behalf of all authors of the Work (as defined in the below author licence), an exclusive licence and/or a non-exclusive licence for contributions from authors who are: i) UK Crown employees; ii) where BMJ has agreed a CC-BY licence shall apply, and/or iii) in accordance with the terms applicable for US Federal Government officers or employees acting as part of their official duties; on a worldwide, perpetual, irrevocable, royalty-free basis to BMJ Publishing Group Ltd ("BMJ") its licensees and where the relevant Journal is co-owned by BMJ to the co-owners of the Journal, to publish the Work in BMJ Open and any other BMJ products and to exploit all rights, as set out in our licence.

The Submitting Author accepts and understands that any supply made under these terms is made by BMJ to the Submitting Author unless you are acting as an employee on behalf of your employer or a postgraduate student of an affiliated institution which is paying any applicable article publishing charge ("APC") for Open Access articles. Where the Submitting Author wishes to make the Work available on an Open Access basis (and intends to pay the relevant APC), the terms of reuse of such Open Access shall be governed by a Creative Commons licence – details of these licences and which Creative Commons licence will apply to this Work are set out in our licence referred to above.

For peer review only

REFERENCES

1. Meding, B. and B. Jarvholm, *Hand eczema in Swedish adults - Changes in prevalence between 1983 and 1996.* Journal of Investigative Dermatology, 2002. **118**(4): p. 719-723.
2. Diepgen, T.L., et al., *Management of chronic hand eczema.* Contact Dermatitis, 2007. **57**(4): p. 203-210.
3. Cvetkovski, R.S., et al., *Relation between diagnoses on severity, sick leave and loss of job among patients with occupational hand eczema.* British Journal of Dermatology, 2005. **152**(1): p. 93-98.
4. Fowler, J.E., et al., *Impact of chronic hand dermatitis on quality of life, work productivity, activity impairment, and medical costs.* Journal of the American Academy of Dermatology, 2006. **54**(3): p. 448-457.
5. Diepgen, T.L., et al., *Cost of illness from occupational hand eczema in Germany.* Contact Dermatitis, 2013. **69**(2): p. 99-106.
6. Meding, B., K. Wrangsjo, and B. Jarvholm, *Fifteen-year follow-up of hand eczema: persistence and consequences.* British Journal of Dermatology, 2005. **152**(5): p. 975-980.
7. English, J., et al., *Consensus statement on the management of chronic hand eczema.* Clinical and Experimental Dermatology, 2009. **34**(7): p. 761-769.
8. Diepgen, T.L., et al., *Hand eczema classification: a cross-sectional, multicentre study of the aetiology and morphology of hand eczema.* British Journal of Dermatology, 2009. **160**(2): p. 353-358.
9. Politiek, K., et al., *Hyperkeratotic hand eczema: Eczema or not?* 2020. **83**(3): p. 196-205.
10. Park, J.Y., et al., *The histopathological differentiation between palmar psoriasis and hand eczema: a retrospective review of 96 cases.* 2017. **77**(1): p. 130-135.
11. Kolesnik, M., et al., *Eczema in Psoriatico: An Important Differential Diagnosis Between Chronic Allergic Contact Dermatitis and Psoriasis in Palmoplantar Localization.* 2018. **98**(1).
12. Benson, N.R., et al., *An analysis of select pathogenic messages in lesional and non-lesional psoriatic skin using non-invasive tape harvesting.* Journal of Investigative Dermatology, 2006. **126**(10): p. 2234-2241.
13. Wittersheim, M., et al., *Differential expression and in vivo secretion of the antimicrobial peptides psoriasin (S100A7), RNase 7, human beta-defensin-2 and-3 in healthy human skin.* Experimental Dermatology, 2013. **22**(5): p. 364-366.
14. Molin, S., et al., *Filaggrin mutations may confer susceptibility to chronic hand eczema characterized by combined allergic and irritant contact dermatitis.* British Journal of Dermatology, 2009. **161**(4): p. 801-807.
15. Thyssen, J.P., *The association between filaggrin mutations, hand eczema and contact dermatitis: a clear picture is emerging.* British Journal of Dermatology, 2012. **167**(6): p. 1197-1198.
16. Thyssen, J.P., *Atopic dermatitis, filaggrin mutations and irritant contact dermatitis.* British Journal of Dermatology, 2013. **168**(2): p. 233-234.

For peer review only - http://bmjopen.bmj.com/site/about/guidelines.xhtml

17. Thyssen, J.P., et al., *Filaggrin null mutations increase the risk and persistence of hand eczema in subjects with atopic dermatitis: results from a general population study.* British Journal of Dermatology, 2010. **163**(1): p. 115-120.
18. Thyssen, J.P., et al., *Filaggrin mutations are strongly associated with contact sensitization in individuals with dermatitis.* Contact Dermatitis, 2013. **68**(5): p. 273-276.
19. Giwercman, C., et al., *Classification of atopic hand eczema and the filaggrin mutations.* Contact Dermatitis, 2008. **59**(5): p. 257-260.
20. Paulden, M., et al., *Alitretinoin for the treatment of severe chronic hand eczema.* Health Technology Assessment, 2010. **14**: p. 39-46.
21. Smith, I.L., et al., *Treatment of severe, chronic hand eczema: results from a UK-wide survey.* Clin Exp Dermatol, 2016.
22. Schempp, C.M., et al., *Treatment of chronic palmoplantar eczema with local bath-PUVA therapy.* Journal of the American Academy of Dermatology, 1997. **36**(5): p. 733-737.
23. Coenraads, P.J., *Hand Eczema.* New England Journal of Medicine, 2012. **367**(19): p. 1829-1837.
24. Diepgen, T.L., et al., *Guideline on the Management of Hand Eczema ICD-10 Code: L20. L23. L24. L25. L30.* JDDG: Journal der Deutschen Dermatologischen Gesellschaft, 2009. **7**(s3).
25. Ruzicka, T., et al., *Efficacy and safety of oral alitretinoin (9-cis retinoic acid) in patients with severe chronic hand eczema refractory to topical corticosteroids: results of a randomized, double-blind, placebo-controlled, multicentre trial.* British Journal of Dermatology, 2008. **158**(4): p. 808-817.
26. Ling, T.C., et al., *British Association of Dermatologists and British Photodermatology Group guidelines for the safe and effective use of psoralen-ultraviolet A therapy 2015.* British Journal of Dermatology, 2016. **174**(1): p. 24-55.
27. Held, E., et al., *The hand eczema severity index (HECSI): a scoring system for clinical assessment of hand eczema. A study of inter- and intraobserver reliability.* British Journal of Dermatology, 2005. **152**(2): p. 302-307.
28. Basra, M.K.A., et al., *The Dermatology Life Quality Index 1994-2007: a comprehensive review of validation data and clinical results.* British Journal of Dermatology, 2008. **159**(5): p. 997-1035.
29. Augustin, M., et al., *The patient benefit index: a novel approach in patient-defined outcomes measurement for skin diseases.* Archives of Dermatological Research, 2009. **301**(8): p. 561-571.
30. Blome, C., et al., *Measurement of patient-relevant benefits in the treatment of chronic hand eczema - a novel approach.* Contact Dermatitis, 2009. **61**(1): p. 39-45.
31. Cowdell, F., Ersser, S., Gradwell, C., Langford, A., *The development, validity, reliability and field testing of the Person- Centred Dermatology Self-Care Index.* Archives of Dermatological Research, 2012. **148**: p. 1251-1256.

For peer review only

For peer review only - http://bmjopen.bmj.com/site/about/guidelines.xhtml

32. Cowdell, F., et al., *The Person-Centered Dermatology Self-Care Index A Tool to Measure Education and Support Needs of Patients With Long-term Skin Conditions.* Archives of Dermatology, 2012. **148**(11): p. 1251-1255.
33. EuroQol, G., *EuroQol--a new facility for the measurement of health-related quality of life.* Health Policy, 1990. **16**(3): p. 199-208.
34. Coenraads, P.J., et al., *Construction and validation of a photographic guide for assessing severity of chronic hand dermatitis.* British Journal of Dermatology, 2005. **152**(2): p. 296-301.
35. Gamble, C., et al., *Guidelines for the content of statistical analysis plans in clinical trials.* 2017. **318**(23): p. 2337-2343.
36. Moher, D., et al., *The CONSORT statement: revised recommendations for improving the quality of reports of parallel-group randomized trials.* 2001. **285**(15): p. 1987-1991.
37. NICE. (2013). *Guide to the methods of technology appraisal 2013*. Retrieved November 20, 2018 from https://www.nice.org.uk/process/pmg9/resources/guide-to-the-methods-of-technology-appraisal-2013-pdf-2007975843781
38. Vilsbøll, A.W., Kragh, N., Hahn-Pedersen, J. *et al.* Mapping Dermatology Life Quality Index (DLQI) scores to EQ-5D utility scores using data of patients with atopic dermatitis from the National Health and Wellness Study. *Qual Life Res* **29,** 2529–2539 (2020)
39. Niall J. Davison., et al., *Generating EQ-5D-3L Utility Scores from the Dermatology Life Quality Index: A Mapping Study in Patients with Psoriasis*. Value in Health 2018 **21**(8) 2018, p1010-1018
40. Ali FM, Kay R, Finlay AY, et al. Mapping of the DLQI scores to EQ-5D utility values using ordinal logistic regression. *Qual Life Res*. 2017;26(11):3025-3034. doi:10.1007/s11136-017-1607-4

For peer review only

For peer review only - http://bmjopen.bmj.com/site/about/guidelines.xhtml

SUPPLEMENTARY MATERIAL 1

Table 2 ALPHA Trial Assessment Schedule

Weeks		0	4	8	12	16	20	24	28	32	36	44	52
Trial period	Screening	Baseline	Interventional			Interventional/ Follow-up			Follow-up				
Informed Consent (Full/Shortened/Optional photography)	X	X											
Registration	X												
Eligibility blood sample & IgE tests	X												
Start contraception prevention program (if applicable)	X	X											
Eligibility assessment		X											
Medical history, Clinical assessment, Gene Variant analysis blood sample		X											
PeDeSI by treating clinician/unblinded nurse		X			X								X
PGA by treating clinician		X	X	X	X	X	X	X	X	X	X	X	X
PGA, HECSI by blinded assessor		X	X	X	X	X	X	X	X	X	X	X	X
mTLSS by blinded assessor		X			X			X			X		X
Nail assessment by blinded assessor (Bradford recruited participants only)		X	X	X	X	X	X	X	X	X	X	X	X
DLQI		X	X	X	X	X	X	X	X	X	X	X	X
PBI-HE, EQ-5D-3L		X			X			X			X		X
Health resource utilisation questionnaire					X			X			X		X
Provide standard education for HE		X											
Randomisation		X											
Tape stripping sample collection (for randomly selected participants only)		X											
Photograph hands		X			X								
Randomised treatment compliance (medication diary review)			X	X	X	X	X	X					
Topical corticosteroid usage (medication diary review)			X	X	X	X	X	X	X	X	X	X	X
Details of treatment under ‘standard clinical practice’						X	X	X	X	X	X	X	X
Reportable Adverse reactions and Related SAEs/SARs/SUSARs			X	X	X	X	X	X	X	X	X	X	X

For peer review only

SUPPLEMENTARY MATERIAL 2 - CONCOMITANT TREATMENTS

Concomitant treatments which are NOT permitted during the interventional phase of the trial are detailed below;

For all participants:

- Topical calcineurin antagonists
- New systemic corticosteroids for reasons other than hand eczema

For participants randomised to PUVA:

- Medication that may act as significant photosensitisers (e.g. tetracycline antibiotics) according to phototherapy guidelines.

For participants randomised to Alitretinoin:

- Systemic tetracycline antibiotics
- Other vitamin A derivatives.
- Other drugs with potential for drug-drug interaction (e.g. CYP3A4 inhibitor ketoconazole).

For peer review only

SUPPLEMENTARY MATERIAL 3 – HANDLING OF BIOLOGICAL SAMPLES

Consent

Informed, written consent for the blood sample/s must be obtained prior to registration and prior to the participant undergoing procedures that are specifically for the purposes of the trial and are not standard routine care at the participating sites.

Gene variant analysis blood sample

A blood sample is taken at baseline (although also possible at any later visit) in order to obtain DNA for subsequent analysis for the filaggrin mutation and other skin barrier molecule polymorphisms. These samples will be anonymised and sent to Professor Ann Morgan's Molecular Rheumatology laboratory (Leeds Institute Cardiovascular and Metabolic Medicine, The LIGHT Laboratories, Clarendon Way, Leeds) for DNA extraction.

For blood samples that have been damaged or classed as unusable during transit to the laboratory, the CTRU will contact research sites to request a replacement sample is obtained, if possible, from the participant at the next trial visit.

A proportion of extracted DNA will be sent to Professor Stephan Weidinger's laboratory (Department of Dermatology, University Hospital Schleswig-Holstein, Kiel, Germany) for assessment of skin barrier molecule polymorphisms (including filaggrin loss-of-function mutation). The remainder of the extracted DNA will be placed in long term storage (under suitable conditions) in the laboratory of Anne Morgan for potential future research. For example, there is evidence linking PUVA treatment and mutations in the vitamin D receptor and this sample set will provide an invaluable resource to delineate any causal link.

Biomarker sub study

A subset of 100 participants recruited from selected centres will be asked to provide written informed consent to this optional sub study to consent to the following procedures, which will be collected prior to the start of the randomised treatment:

- **Tape stripping technique** involves an adhesive to bind and remove the top epidermal layers. This approach has been used as a non-invasive technique for direct sampling of skin [17, 18]. Tape stripping is generally rapid, and patient-friendly. For detailed instructions please refer to the biomarker sub study work instruction. Tape strips will be submersed in buffer solution and stored at site at -80°C prior to sending to Dr Miriam Wittmann's laboratory (Section of Musculoskeletal Disease, Leeds Institute of Molecular Medicine, Wellcome Trust Brenner Building, St. James's University Hospital, Leeds).

- **Skin washing:** Where tape stripping is not possible due to acute inflammation, mediator content can be measured in skin washing fluids [47]. For detailed instructions please refer to the biomarker sub study work instruction. Buffer scrubs (washing fluid) will be stored at site at -80°C prior to sending to Dr Miriam Wittmann's laboratory (Section of Musculoskeletal Disease, Leeds Institute of Molecular Medicine, Wellcome Trust Brenner Building, St. James's University Hospital, Leeds, UK)

For peer review only

Delete this line, then print first page of Information Sheet and Consent Form on Trust/Hospital headed paper

Comparison of Alitretinoin with PUVA as the first line treatment in patients with severe chronic hand eczema

PARTICIPANT INFORMATION SHEET AND INFORMED CONSENT DOCUMENT

A large-print version of this sheet is available on request.

You have been invited to take part in a research study called **ALPHA**. Before you decide if you want to take part, we would like to explain why the research is being done, how we will use any information about you, and what the study will involve.

Please read this information carefully, and discuss it with others if you like. Ask us if anything is unclear, or if you would like more information.

Once you have read this information, your doctor will talk to you about the study again and you can ask any questions you like.

- Part 1 explains the purpose of this study and what will happen to you if you take part.
- Part 2 gives you more detailed information about the conduct of the study.

Please take time to decide whether or not you wish to take part.

How to contact us

If you have any questions about this study, please talk to your doctor at

<<Enter PI, nurse name >>

<< Contact details for site>>

Thank you for reading this information sheet.

For peer review only

Part 1

What is the purpose of the study?

The purpose of the ALPHA research study is to find out the most effective treatment for chronic hand eczema. The two most common treatments include (1) exposure of hands to ultra violet (UV) light after they have been soaked in a solution called psoralen (a process referred to as PUVA) and (2) the tablet treatment Alitretinoin. Although both of these treatments are equally used by doctors in standard clinical practice, there is no clear evidence indicating which of these two treatments is most effective in treating which type of hand eczema. This study will directly compare these two treatments to examine both the short term and longer term effectiveness of each treatment in terms of both how good the hands heal with the treatment and how long the skin can remain clear once healed.

Why have I been chosen?

You have been approached because you are suffering from chronic hand eczema, which has not improved with strong steroid treatment for at least the last 4 weeks and has been defined as 'severe' by your doctor.

The study is taking place in several other hospitals around the UK and we are hoping that 780 participants like you who have chronic hand eczema will take part.

Do I have to take part?

No, your participation in the ALPHA trial is voluntary and you may withdraw your consent to take part at any time, without giving us a reason.

If you decide to take part you will be given this information document to keep. You will be asked to sign a consent form, but you are still free to withdraw at any time and without giving a reason. If you decide not to take part, your doctor will be happy to talk through alternative treatment options for your eczema. Your treatment and care will not be affected in any way.

What will happen to me if I take part?

The best way of finding out which is the most effective treatment is in a randomised study. 'Randomised' means that a treatment (PUVA or Alitretinoin) will be allocated to you randomly. Neither your doctor nor you will choose which treatment you receive. In this way, a fair comparison can be made.

First we need to make sure that it is safe for you to take part and that you are suitable for this study. To do this you will have some screening tests.

- **Screening visit:**

You will need to have a blood test taken (if you have not had one taken within the last 3 months) to determine whether you are suitable to take Alitretinoin or not. This is because Alitretinoin can, in some participants, increase levels of cholesterol and natural fats (lipids) within the blood, and should not be taken by participants who have significant liver problems. As we do not know at this stage which participant will receive Alitretinoin, we will collect a blood sample from all participants. This test is to make sure that it is safe for you to take Alitretinoin if you are randomised to this treatment in the study.

We will also collect a blood sample to screen for atopy which is the tendency to develop the classic allergic diseases (atopic dermatitis, allergic rhinitis (hay fever), and asthma). This is routine for all participants who have severe hand eczema. Therefore the blood sample will not be taken if the results are already available in your hospital notes.

For peer review only - http://bmjopen.bmj.com/site/about/guidelines.xhtml

For peer review only

Your study doctor may ask you questions about your medical history and medications you are currently taking, and examine your hands to make sure you are eligible to participate.

Women of child bearing age will be asked at this visit to agree to use a reliable form of effective contraception (if you are not already doing so) and follow a strict pregnancy prevention program. This is standard care for all participants who are considered for Alitretinoin treatment. As you will not know at this stage what treatment you will be randomised to at the baseline visit, all participants will be expected to follow the strict pregnancy prevention program and use a reliable form of effective contraception for at least one month before the treatment has started. If you are then randomised to receive Alitretinoin, you will be required to continue to follow the strict pregnancy program throughout the trial and one month after the treatment has stopped.

If you are randomised to PUVA, it is not common clinical practice to follow the strict pregnancy program. However, the study doctor will advise that you should not become pregnant during the trial and recommend that you use a reliable form of contraception.

If the blood test shows that it is not suitable or safe for you to take part in this study, your doctor will discuss other treatment options with you. Any information we have collected about you will still be used but you will not continue on the study.

- **Baseline visit:**

If your screening test confirms that you are able to take part in the study, your hands will be examined by both the study doctor and an independent assessor (doctor or research nurse), who does not know your randomised treatment. You will be asked to provide another blood sample so that we can look at the genetic makeup of proteins (such as filaggrin) known to be important in eczema.

We will ask you to fill in some questionnaires about your hands and your health in general.

For women of child bearing age a pregnancy test will be done to make sure you are not pregnant.

You will then receive standard education in using moisturisers, emollients, avoiding irritants and relevant contact allergens and steroid cream use before being randomised to a treatment.

You will also be provided with a medication diary to complete, so that you can record when you have received your randomised treatment and how often you use steroid creams to control your hand eczema. In order to help you with this aspect of the study you can sign up to a text reminder service, if you wish, where you will receive a text once a week to remind you to complete your medication diary. This service is optional. It will be important that you bring your diary to each of your visits.

- **Treatment visits (interventional phase):**

If you are randomised to receive Alitretinoin, you will need to take the tablets once a day with your main meal as instructed by your study doctor for 12 weeks.

If you are randomised to receive PUVA you will attend twice weekly for PUVA treatment for 12 weeks. Please note that the bath solution that you soak your hands in for the trial PUVA treatment will be obtained from a different supplier but will be used at the same dose as the standard bath solution used in NHS practice.

Depending on how your hand eczema is responding to either treatment, you may be asked to continue treatment for up to a further 12 weeks.

During this 12 week period, you will be asked to attend clinic every 4 weeks where you will be asked to complete some questionnaires about your hands and your health, and your hands will be assessed.

For peer review only - http://bmjopen.bmj.com/site/about/guidelines.xhtml

- **Follow up visits**

You will be asked to attend once every 4 weeks for 24 weeks. At these visits, you will again be asked to complete some questionnaires about your hands and your health, and your hands will be assessed.

It is important that you do not tell the independent assessor which treatment you are receiving.

Optional photography

A selection of participants will be chosen at random and asked if they would like to consent to having photographs taken of their hands at the start of the study and after 12 weeks. These photographs will be reviewed to ensure that assessment of chronic hand eczema is consistent across all hospitals in the study. If you would prefer not to be photographed then you can opt out of photography, but still continue with other parts of the trial. The photographs will only be of your hands (not your face) so you can not be identified from the photographs.

How long does treatment go on?

You will receive your randomised treatment for a period of 12 weeks. At this point, if your doctor feels that your condition has cleared sufficiently, or is not responding sufficiently, then your randomised treatment will be stopped. However, if your condition has not cleared, but has shown signs of improvement then your doctor may advise that you continue your randomised treatment in the same way for up to a further 12 weeks. Your doctor will assess your condition every 4 weeks until week 24 to see if your hand eczema has got better. If this happens, you will stop receiving your randomised treatment. You will only receive your randomised treatment for a maximum of 24 weeks.

What if the treatment doesn't help?

If, after 12 or 24 weeks, your randomised treatment is not helping your condition, then your doctor will stop this treatment and treat you according to local standard practice. For example if you don't respond to Alitretinoin, then you may be switched to PUVA treatment or other standard treatments such as immunomodulators or other Vitamin A derivatives. This will be decided by your study doctor.

You will be able to use moisturisers, emollients, soap substitutes and/or bath oils on a daily basis throughout the study, and topical steroids when you feel that you need to use them to control your hand eczema.

How is my condition monitored?

Your doctor will continue to assess your skin throughout the study even if your eczema has cleared; this is so that the doctor will be able to treat your skin according to standard care practice if your eczema starts to get worse.

Unwanted effects of treatment

All the study treatments are used routinely in standard care; however there are some known side effects.

Alitretinoin:

Alitretinoin can, in some participants, increase lipid levels within the blood (30mg dose affects 1 in 3; 10mg dose affects 1:6), so your doctor will check for these with regular blood tests. Alitretinoin has been shown to cause headaches in some cases (30mg dose affects 1 in 5 participants), although these are usually eased or stopped by reducing the dose of Alitretinoin (10mg dose affects 1 in 10 participants).

Other potential side effects reported for Alitretinoin include dryness of the eyes and skin (between 1:10 and 1:100 participants), muscle and joint pain (between 1:10 and 1:100 participants) and mood swings

For peer review only - http://bmjopen.bmj.com/site/about/guidelines.xhtml

(only very few; the exact number is unknown). If you experience unusual depressive mood changes please discontinue the tablet and talk to your study doctor.

Alitretinoin can harm an unborn baby; therefore you must not take part in this study if you are pregnant. You must not become pregnant during the study period or for 1 month after your last study dose. If you are at risk of becoming pregnant you need to use a reliable form of effective contraception before you start treatment and during the study. If you become pregnant during the study, you must tell your study doctor at once so that your study treatment can be stopped immediately. You will be referred to an obstetrician/gynaecologist experienced in reproductive toxicity for further evaluation, counselling and advice on the potential risks to your unborn child and the options available to you. Your pregnancy will be followed closely by the study team up until final outcome.

Once you have completed the study or if you withdraw from the study and you become pregnant during the 1 month period after your last dose of study drug, you should still tell your study doctor as soon as possible.

PUVA:

There is a small risk that your skin may burn, with redness and soreness (less than 1 in 100) and very rarely blistering (less than 1 in 200, particularly in participants of fair complexion). Some people may develop a rash (polymorphic light eruption) or may experience pain and itching related to the PUVA therapy. Should that happen, your treatment will be discontinued until symptoms improve. However the risk of burning is minimised by adapting the UVA dose to your skin type and your skin response to treatment by the phototherapy staff. UV light therapy can increase the risk of skin cancer. This risk may gradually increase with long term UV application (e.g. over 200 treatments). However, a low number of exposures (48) will be provided as the randomised treatment in this trial.

PUVA treatment is not thought to cause any harm to an unborn baby. However, this information is based on limited available data. Therefore, it is recommended not to become pregnant during treatment and, if at risk of pregnancy, you should use a reliable contraception before the treatment has started, during treatment and for 3 months after the treatment has stopped, unless advised otherwise by the dermatologist or staff within the phototherapy department.

If you become pregnant during the study, you must tell your study doctor at once who will stop your study treatment immediately. Your pregnancy will be followed closely by your doctor and the study team until final outcome.

What are the possible disadvantages and risks of taking part?

Taking part in this research study involves time and commitment such as regular hospital visits for treatment and follow up visits. Although the number of treatment visits are no more than if you were receiving these treatments outside of a research study setting, the follow up visits are in addition. However, your travel expenses will be paid for the follow up visits.

The treatments that you may receive as part of the ALPHA trial are used as routine standard by the NHS and therefore there are no additional risks beyond those that you would be exposed to as part of your standard care.

What are the possible benefits of taking part?

It is hoped by taking part in this study you will respond to a treatment and have an increased quality of life. This is in line with what you would have experienced if treated according to normal NHS practice, as both treatments are used as standard NHS treatments. However, the ALPHA trial will help to understand which of these treatments, if any, is more effective in the short term, and what the long term benefits of each treatment may be.

For peer review only - http://bmjopen.bmj.com/site/about/guidelines.xhtml

What if I would like to take part but I have trouble with or am unable to write?

If you would like to take part but cannot or find it difficult to write, you can have a witness (e.g. a friend, a family member, or member of your healthcare team) to complete the written part of the consent for you and the questionnaires during the study. The witness will only act to help you carry out your wishes – you are free to change your mind at any time.

What if something goes wrong?

It is very unlikely that you will come to any harm as a result of taking part in this study, as both treatments are already used widely across the NHS. If you have a concern about any aspect of this study, you should ask to speak with your nurse or doctor. If you remain unhappy and wish to complain formally, you can do this through the NHS Complaints Procedure. Details about how to complain can be obtained from staff on the ward or by contacting your local Patient Advice and Liaison Service (PALS) http://www.pals.nhs.uk/ or Patient Advice & Support Service (PASS) (Scotland) or your local Trust complaints department as detailed below;

<< Insert contact details for local complaints department>>

What happens when the research study stops?

At the end of the study your doctor will discuss available ongoing treatment options with you, if required.

Will my taking part be kept confidential?

Yes. If you decide to participate in the ALPHA trial, the information collected about you will be handled strictly in accordance with the consent that you have given and also the 1998 Data Protection Act. Please refer to Part 2 for further details.

Contact Details

If you have any further questions about your illness or clinical studies, please discuss them with your doctor. If you would like further information about clinical research, the UK Clinical Research Collaboration (a partnership of organisations working together on clinical research in the UK) has published a booklet entitled 'Understanding Clinical Trials'. Contact UKCRC: Tel: 0207 670 5452; website www.ukcrc.org

This completes Part 1 of the Information Sheet. If the Information in Part 1 has interested you and you are considering participation, please continue to read the additional information in Part 2 before making any further decisions.

Part 2

What if relevant new information becomes available?

Sometimes during the course of a study, new information becomes available. If this happens your doctor will tell you about it and discuss with you whether you want to continue in the study. If you decide not to continue, your doctor will continue your care if this is necessary. If you decide to continue you may be asked to sign an updated consent form. Occasionally on receiving new information, your doctor may consider it to be in your best interest to withdraw you from further study treatment.

What will happen if I don't want to carry on with the study?

If you withdraw consent from further study treatment, information will still be collected about you and will be included in the final study analysis, unless you request otherwise. If you withdraw consent for further data collection your data collected to that point will remain on file and will be included in the final study

For peer review only - http://bmjopen.bmj.com/site/about/guidelines.xhtml

analysis. The ALPHA Study Team may be required to collect some limited information about any side effects you may have as a result of taking part in the trial. This will only be collected if required by the Regulatory Authorities. In line with Good Clinical Practice guidelines, at the end of the study, your data will be securely archived for a period of 15 years. Arrangements for confidential destruction will then be made.

Who has organised, reviewed and funded the research and who will be supervising it?

The ALPHA trial is being organised by the University of Leeds through the Clinical Trials Research Unit (CTRU) in collaboration with Leeds Institute of Rheumatic and Musculoskeletal Medicine, Chapel Allerton Hospital, Leeds. The study has been reviewed by the Leeds West Research Ethics Committee and the Research and Development Department situated at your hospital. The study is funded by the National Institute for Health Research (NIHR) Health Technology Assessment (HTA) programme

What if there is a problem?

Harm:

Every care will be taken in the course of this clinical trial. However, in the unlikely event that you are injured as a result of the managing organisation (University of Leeds), compensation may be available and you may have to pay your related legal costs. The hospital where you receive your treatment has a duty of care to you whether or not you agree to participate in the trial and the University of Leeds accepts no liability for negligence on the part of your hospital's employees. If you wish to complain about any aspect of the way you have been treated please contact your research doctor in the first instance.

Any claims will be subject to UK law and must be brought in the UK.

If you have private medical insurance, you should tell your insurer that you are taking part in research. They will let you know if it affects your policy.

Will my taking part in this study be kept confidential?

If you decide to participate in ALPHA, the information collected about you will be handled in accordance with the consent that you have given, the 1998 Data Protection Act and General Data Protection Regulation (GDPR, please see additional information at the end of this information sheet). The information needed for study purposes will be collected on paper forms and sent (usually using standard Royal Mail post but in some cases by fax or email) from the hospital to the Clinical Trials Research Unit (CTRU). You will be allocated a study number, which will be used along with your date of birth and initials to identify you on each paper form. The consent form will also record your NHS number; Your full name will be included on your consent form and a copy of this and your mobile telephone contact details (with your permission) will be sent to the CTRU by fax, post or encrypted email. This is to enable the CTRU to send text reminders to your mobile phone about regularly completing your medication diary. Every effort will be made to ensure that any further information about you that leaves the hospital will have your name and address removed so that you cannot be recognised from it; this information will usually be removed by a member of the study team at your hospital, but may also be removed by the CTRU upon receipt.

Your data will be entered onto a secure database held at the CTRU in accordance with the 1998 Data Protection Act and GDPR.

Your healthcare records may be looked at by authorised individuals from the research team, the University of Leeds (the study Sponsor, i.e. the organisation which takes responsibility for the initiation, management and/or financing of a clinical trial) or the regulatory authorities to check that the study is being carried out correctly.

For peer review only - http://bmjopen.bmj.com/site/about/guidelines.xhtml

The information collected about you may be shared with other research teams to answer new research questions in the future. Wherever possible, information will be anonymised (i.e. your full name will not be disclosed).

Your data may be passed to other organisations (possibly in other countries where the data protection standards and laws are different to the UK) to monitor the safety of the treatment(s) that you are receiving; this data will have your name removed.

For selected participants, photographs of the hands will be sent via a secure data transfer system administered by the CTRU. Wherever possible, this data will be anonymised and your name removed.

Involvement of the General Practitioner/Family Doctor (GP):

Your GP, and the other doctors involved in your healthcare, will be kept informed of your participation in this study.

Will any genetic tests be done?

Yes. If you agree to take part in ALPHA, a blood sample will be taken at your baseline visit. This sample will be used to test whether you have particular genetic markers which influence your type of hand eczema or your response to therapy. In particular, it has been shown that genetic variations in a protein called filaggrin makes carriers more likely to suffer from chronic hand eczema. Your blood sample (labelled with only your study ID number, date of birth and initials) will be sent to researchers at the Leeds Institute of Rheumatic and Musculoskeletal Medicine, Chapel Allerton Hospital, Leeds and examined as part of the ALPHA trial in accordance with this consent. Part of the genetic material obtained from the blood sample will be sent to a University hospital laboratory in Kiel, Germany, which specialises in eczema genetics, for analysis of genetic markers linked to hand eczema. Neither the laboratory in Leeds or Kiel will know your identity, so you will not receive the results of the analysis.

The remaining part of the genetic material from your blood sample and data may be stored, and may provide a resource for future studies in the field of chronic hand eczema. If any information from this study is used to develop new research, data protection regulations will be observed and strict confidentiality maintained; your data will have your personal details removed, but will be coded so it may be linked back to your details. You will not be identified in the results of future studies; however ethical approval will be obtained for any future studies involving your data or samples .

What will happen to the results of the research study?

When the study is completed the results will be published in a medical journal, but no individual participants will be identified. The study results will be available on the CTRU ALPHA website. If you would like to obtain a copy of the published results, please ask your doctor.

If you are interested in following the progress of the study, please follow us on twitter @LICTR_Alpha.

Thank you for taking time to read this.

For peer review only

For peer review only - http://bmjopen.bmj.com/site/about/guidelines.xhtml

Delete this line, then print on Trust/Hospital headed paper

Participant ID:	Initials:
Date of Birth:	NHS/Hospital Number:
EudraCT Number:	Principal Investigator:

ALPHA

PARTICIPANT CONSENT FORM

Please initial each box

1. I confirm that I have read and understand the information sheet for the ALPHA study and have had the opportunity to ask questions. ☐

2. I understand that my participation in this study is voluntary and that I am free to withdraw at any time without my medical care or legal rights being affected. I understand that even if I withdraw from the above study, the data and samples collected from me will be used in analysing the results of the study and in some cases further information about any unwanted effects of my treatment may need to be collected by the study team. ☐

3. I understand that my healthcare records may be looked at by authorised individuals from the study team, regulatory bodies or Sponsor in order to check that the study is being carried out correctly. I give permission for these individuals to have access to my information. ☐

4. I agree to allow any information or results arising from this study to be used for healthcare and/or further medical research upon the understanding that my identity will remain anonymous wherever possible. ☐

5. I understand that the research nurse will keep secure records at the hospital which will allow me to be followed up in hospital and at home (including name, date of birth, NHS number, hospital number, address and telephone number). ☐

For peer review only - http://bmjopen.bmj.com/site/about/guidelines.xhtml

Please initial each box

6. I understand that my blood sample will be sent to the Molecular Rheumatology Laboratory at St James's University Hospital in Leeds so they can obtain genetic material. I understand that a sample of my genetic material will be sent to a specialised University research laboratory in Kiel, Germany (Department of Dermatology, University Hospital Schleswig-Holstein) for genetic analysis. I agree to the genetic material sample being stored in both locations and used for additional research investigations that form part of this study. I understand that my initials, date of birth and trial identification number will be sent with my sample (but that my identity will remain anonymous wherever possible). ☐

7. I agree to a copy of this Consent Form containing my name, date of birth and NHS number being sent to the CTRU. ☐

8. I consent to the storage including electronic, of personal information for the purposes of this study. I understand that any information that could identify me will be kept confidential and that no personal information that could identify me will be included in the study report or other publication. ☐

9. I agree that my GP, or any other doctor treating me, will be notified of my participation in this study. ☐

For peer review only

For peer review only - http://bmjopen.bmj.com/site/about/guidelines.xhtml

The following points are OPTIONAL. Even if you agree to take part in this study, you do not have to agree to this section.

	Please initial each box Yes	No
10. I give permission for surplus genetic material obtained from my blood sample may be used for further medical research upon the understanding that my identity will remain anonymous wherever possible. Only my initials, date of birth and trial identification number will be stored with my sample.	☐	☐
11. I give permission for the responses that I give on the PBI-HE questionnaire to be sent to Augustin AG, Germany, to support PBI-HE questionnaire research upon the understanding that my identity will remain anonymous wherever possible.	☐	☐
12. I agree to my mobile telephone contact details being sent to the CTRU for the purposes of sending me text messages as a reminder to complete my medication diary.<br><br>Enter mobile tel number: ………………………………………………	☐	☐
13. I agree to allow the research nurse to take photographs of my hands if selected at random.	☐	☐

Please initial the box

14. I agree to take part in the study. ☐

For peer review only - http://bmjopen.bmj.com/site/about/guidelines.xhtml

Participant:
Signature…………………………………………………………………………………

Name (block capitals)…………………………………………………………………

Date………………………………………………………………………………………

Investigator:
I have explained the study to the above named participant and he/she has indicated his/her willingness to participate.

Signature…………………………………………………………………………………

Name (block capitals)…………………………………………………………………

Date………………………………………………………………………………………

(If used)Translator:
Signature…………………………………………………………………………………

Name (block capitals)…………………………………………………………………

Date………………………………………………………………………………………

Witness:
I have completed this consent form on behalf of the person named above who has freely given their consent to participate.

Signature…………………………………………………………………………………

Name (block capitals)…………………………………………………………………

Date………………………………………………………………………………………

For peer review only

(1 copy for participant; 1 for the CTRU; 1 held in participant notes, original stored in Investigator Site File)

For peer review only - http://bmjopen.bmj.com/site/about/guidelines.xhtml

This information is for people who are taking part in the study:

Comparison of **AL**itretinoin with **P**UVA as the first line treatment in patients with severe chronic **HA**nd eczema

We wanted to contact you to make sure you understand how we use the information we collect about you in the ALPHA study. This is because a new data protection law (called the General Data Protection Regulation, or GDPR for short) came into force in the UK on 25th May 2018. The GDPR means that you must have clear information about how information about you is collected and used.

We want to give you some more information about how the data we collect from you is used, in addition to the patient information sheet you have been given.

- University of Leeds is the Sponsor for the ALPHA study based in the United Kingdom. University of Leeds have asked the Clinical Trials Research Unit (CTRU) at the University of Leeds to run the ALPHA study on their behalf.
- CTRU will be using information from you and your medical records in order to undertake this study and will act as data controller for this study. This means that we are responsible for looking after your information and using it properly. University of Leeds will keep identifiable information about you for 15 years after the study has finished.
- Your rights to access, change or move your information are limited, as we need to manage your information in specific ways in order for the research to be reliable and accurate. If you withdraw from the study, we will keep the information about you that we have already obtained. To safeguard your rights, we will use the minimum personally-identifiable information possible.
- As a University we use personally-identifiable information to conduct research to improve health, care and services. As a publicly-funded organisation, we have to ensure that it is on the public interest when we use personally-identifiable information from people who have agreed to take part in research. This means that when you agree to take part in a research study, we will use your data in the ways needed to conduct and analyse the research study.
- Health and care research should serve the public interest, which means that we have to demonstrate that our research serves the interests of society as a whole. We do this by following the UK Policy Framework for Health and Social Care Research.
- If you wish to raise a complaint on how we have handled your personal data, you can contact our Data Protection Officer who will investigate the matter. If you are not satisfied with our response or believe we are processing your personal data in a way that is not lawful you can complain to the Information Commissioners Office (ICO).
- You can find out more about how we use your information by contacting the University of Leeds Data Protection Officer at
    - Email: DPO@leeds.ac.uk
    - Post: University of Leeds, 11.72 EC Stoner Building, Leeds LS2 9JT
    - Telephone: +44 (0)113 243 1751

For peer review only - http://bmjopen.bmj.com/site/about/guidelines.xhtml

- Your hospital will keep your contact details confidential and will not pass this information to CTRU (unless you have agreed to receive text reminders – see below). Your hospital will use this information as needed, to contact you about the research study, and make sure that relevant information about the study is recorded for your care, and to oversee the quality of the study. Certain individuals from the University of Leeds, CTRU and regulatory organisations may look at your medical and research records to check the accuracy of the research study. Your hospital will keep identifiable information about you from this study for 15 years after the study has finished.
- If you have agreed to receive text reminders CTRU will receive the telephone number you have provided and keep this information for 15 years after the study has finished. No other contact details will be shared.
- CTRU will receive your name and NHS number on the consent form you have signed. The only people at CTRU who will have access to information that identifies you will be people who audit the data collection process. The people who analyse the information will not be able to identify you and will not be able to find out your name. CTRU will keep identifiable information about you from this study for 15 years after the study has finished.
- When you agree to take part in a research study, the information about your health and care may be provided to researchers running other research studies in this organisation and in other organisations. These organisations may be universities, NHS organisations or companies involved in health and care research in this country or abroad. Your information will only be used by organisations and researchers to conduct research in accordance with the UK policy Framework for Health and Social Care Research.
- This information will not identify you and will not be combined with other information in a way that could identify you. The information will only be used for the purpose of health and care research, and cannot be used to contact you or to affect your care. It will not be used to make decisions about future services available to you such as insurance.

We would like to take this opportunity to thank you again for taking the time to read this information.

On behalf of the ALPHA study team

Dr Miriam Wittman
ALPHA Chief Investigator

For peer review only

For peer review only - http://bmjopen.bmj.com/site/about/guidelines.xhtml

STANDARD PROTOCOL ITEMS: RECOMMENDATIONS FOR INTERVENTIONAL TRIALS

SPIRIT 2013 Checklist: Recommended items to address in a clinical trial protocol and related documents*

Section/item	ItemNo	Description	Page
**Administrative information**			
Title	1	Descriptive title identifying the study design, population, interventions, and, if applicable, trial acronym	1, 2
Trial registration	2a	Trial identifier and registry name. If not yet registered, name of intended registry	2
	2b	All items from the World Health Organization Trial Registration Data Set	N/A
Protocol version	3	Date and version identifier	1
Funding	4	Sources and types of financial, material, and other support	16
Roles and responsibilities	5a	Names, affiliations, and roles of protocol contributors	1
	5b	Name and contact information for the trial sponsor	15
	5c	Role of study sponsor and funders, if any, in study design; collection, management, analysis, and interpretation of data; writing of the report; and the decision to submit the report for publication, including whether they will have ultimate authority over any of these activities	16
	5d	Composition, roles, and responsibilities of the coordinating centre, steering committee, endpoint adjudication committee, data management team, and other individuals or groups overseeing the trial, if applicable (see Item 21a for data monitoring committee)	15

For peer review only

**Introduction**			
Background and rationale	6a	Description of research question and justification for undertaking the trial, including summary of relevant studies (published and unpublished) examining benefits and harms for each intervention	4, 5
	6b	Explanation for choice of comparators	4, 5
Objectives	7	Specific objectives or hypotheses	5, 6
Trial design	8	Description of trial design including type of trial (eg, parallel group, crossover, factorial, single group), allocation ratio, and framework (eg, superiority, equivalence, noninferiority, exploratory)	6
**Methods: Participants, interventions, and outcomes**			
Study setting	9	Description of study settings (eg, community clinic, academic hospital) and list of countries where data will be collected. Reference to where list of study sites can be obtained	6
Eligibility criteria	10	Inclusion and exclusion criteria for participants. If applicable, eligibility criteria for study centres and individuals who will perform the interventions (eg, surgeons, psychotherapists)	6, 7
Interventions	11a	Interventions for each group with sufficient detail to allow replication, including how and when they will be administered	9, 10
	11b	Criteria for discontinuing or modifying allocated interventions for a given trial participant (eg, drug dose change in response to harms, participant request, or improving/worsening disease)	10
	11c	Strategies to improve adherence to intervention protocols, and any procedures for monitoring adherence (eg, drug tablet return, laboratory tests)	11
	11d	Relevant concomitant care and interventions that are permitted or prohibited during the trial	7, 8 Supplementary material 1

For peer review only - http://bmjopen.bmj.com/site/about/guidelines.xhtml

Outcomes	12	Primary, secondary, and other outcomes, including the specific measurement variable (eg, systolic blood pressure), analysis metric (eg, change from baseline, final value, time to event), method of aggregation (eg, median, proportion), and time point for each outcome. Explanation of the clinical relevance of chosen efficacy and harm outcomes is strongly recommended	11, 12
Participant timeline	13	Time schedule of enrolment, interventions (including any run-ins and washouts), assessments, and visits for participants. A schematic diagram is highly recommended (see Figure)	8, 9 Supplementary material 3
Sample size	14	Estimated number of participants needed to achieve study objectives and how it was determined, including clinical and statistical assumptions supporting any sample size calculations	12
Recruitment	15	Strategies for achieving adequate participant enrolment to reach target sample size	6
**Methods: Assignment of interventions (for controlled trials)**			
Allocation:			
Sequence generation	16a	Method of generating the allocation sequence (eg, computer-generated random numbers), and list of any factors for stratification. To reduce predictability of a random sequence, details of any planned restriction (eg, blocking) should be provided in a separate document that is unavailable to those who enrol participants or assign interventions	9
Allocation concealment mechanism	16b	Mechanism of implementing the allocation sequence (eg, central telephone; sequentially numbered, opaque, sealed envelopes), describing any steps to conceal the sequence until interventions are assigned	9
Implementation	16c	Who will generate the allocation sequence, who will enrol participants, and who will assign participants to interventions	9

Blinding (masking)	17a	Who will be blinded after assignment to interventions (eg, trial participants, care providers, outcome assessors, data analysts), and how	11
	17b	If blinded, circumstances under which unblinding is permissible, and procedure for revealing a participant's allocated intervention during the trial	N/A
**Methods: Data collection, management, and analysis**			
Data collection methods	18a	Plans for assessment and collection of outcome, baseline, and other trial data, including any related processes to promote data quality (eg, duplicate measurements, training of assessors) and a description of study instruments (eg, questionnaires, laboratory tests) along with their reliability and validity, if known. Reference to where data collection forms can be found, if not in the protocol	11
	18b	Plans to promote participant retention and complete follow-up, including list of any outcome data to be collected for participants who discontinue or deviate from intervention protocols	11
Data management	19	Plans for data entry, coding, security, and storage, including any related processes to promote data quality (eg, double data entry; range checks for data values). Reference to where details of data management procedures can be found, if not in the protocol	11
Statistical methods	20a	Statistical methods for analysing primary and secondary outcomes. Reference to where other details of the statistical analysis plan can be found, if not in the protocol	12, 13, 14
	20b	Methods for any additional analyses (eg, subgroup and adjusted analyses)	13
	20c	Definition of analysis population relating to protocol non-adherence (eg, as randomised analysis), and any statistical methods to handle missing data (eg, multiple imputation)	12

For peer review only - http://bmjopen.bmj.com/site/about/guidelines.xhtml

**Methods: Monitoring**			
Data monitoring	21a	Composition of data monitoring committee (DMC); summary of its role and reporting structure; statement of whether it is independent from the sponsor and competing interests; and reference to where further details about its charter can be found, if not in the protocol. Alternatively, an explanation of why a DMC is not needed	15
	21b	Description of any interim analyses and stopping guidelines, including who will have access to these interim results and make the final decision to terminate the trial	13
Harms	22	Plans for collecting, assessing, reporting, and managing solicited and spontaneously reported adverse events and other unintended effects of trial interventions or trial conduct	13
Auditing	23	Frequency and procedures for auditing trial conduct, if any, and whether the process will be independent from investigators and the sponsor	N/A
**Ethics and dissemination**			
Research ethics approval	24	Plans for seeking research ethics committee/institutional review board (REC/IRB) approval	14
Protocol amendments	25	Plans for communicating important protocol modifications (eg, changes to eligibility criteria, outcomes, analyses) to relevant parties (eg, investigators, REC/IRBs, trial participants, trial registries, journals, regulators)	14
Consent or assent	26a	Who will obtain informed consent or assent from potential trial participants or authorised surrogates, and how (see Item 32)	8
	26b	Additional consent provisions for collection and use of participant data and biological specimens in ancillary studies, if applicable	9

For peer review only - http://bmjopen.bmj.com/site/about/guidelines.xhtml

Confidentiality	27	How personal information about potential and enrolled participants will be collected, shared, and maintained in order to protect confidentiality before, during, and after the trial	15
Declaration of interests	28	Financial and other competing interests for principal investigators for the overall trial and each study site	16
Access to data	29	Statement of who will have access to the final trial dataset, and disclosure of contractual agreements that limit such access for investigators	16
Ancillary and post-trial care	30	Provisions, if any, for ancillary and post-trial care, and for compensation to those who suffer harm from trial participation	N/A
Dissemination policy	31a	Plans for investigators and sponsor to communicate trial results to participants, healthcare professionals, the public, and other relevant groups (eg, via publication, reporting in results databases, or other data sharing arrangements), including any publication restrictions	2
	31b	Authorship eligibility guidelines and any intended use of professional writers	15
	31c	Plans, if any, for granting public access to the full protocol, participant-level dataset, and statistical code	16
**Appendices**			
Informed consent materials	32	Model consent form and other related documentation given to participants and authorised surrogates	Supplementary material 2
Biological specimens	33	Plans for collection, laboratory evaluation, and storage of biological specimens for genetic or molecular analysis in the current trial and for future use in ancillary studies, if applicable	Supplementary material 4

*It is strongly recommended that this checklist be read in conjunction with the SPIRIT 2013 Explanation & Elaboration for important clarification on the items. Amendments to the protocol should be tracked and dated. The SPIRIT checklist is copyrighted by the SPIRIT Group under the Creative Commons "Attribution-NonCommercial-NoDerivs 3.0 Unported" license.

For peer review only - http://bmjopen.bmj.com/site/about/guidelines.xhtml
