# Supplementary material for: Comparison of ALitretinoin with PUVA as the first-line treatment in patients with severe chronic HAnd eczema (ALPHA): study protocol for a randomised controlled trial
Source: BMJ Open. 2022 Feb 23;12(2):e060029. doi: 10.1136/bmjopen-2021-060029 (PMC8867308; doi:10.1136/bmjopen-2021-060029)
Supplement: Reviewer comments [file bmjopen-2021-060029.reviewer_comments.pdf]

PEER REVIEW HISTORY

BMJ Open publishes all reviews undertaken for accepted manuscripts. Reviewers are asked to complete a checklist review form (**http://bmjopen.bmj.com/site/about/resources/checklist.pdf**) and are provided with free text boxes to elaborate on their assessment. These free text comments are reproduced below.

ARTICLE DETAILS

**TITLE (PROVISIONAL)**	Comparison of ALitretinoin with PUVA as the first line treatment in patients with severe chronic HAnd eczema (ALPHA): study protocol for a randomised controlled trial
**AUTHORS**	Smith, Isabelle; Gilberts, Rachael; Brown, Sarah; Fernandez, Catherine; Nixon, Jane; Reynolds, Catherine; Smith, C; Lear, John; Sunderland, Lesley; Green, Cathy; Goodfield, Mark; Cowdell, Fiona; Hampton, Philip; Barker, Amy; Vargas-Palacios, Armando; Tubeuf, Sandy; Wittmann, Miriam

This article was not externally reviewed at BMJ Open. Protocols that have been independently assessed prior to submission to BMJ Open are usually fast-tracked to publication on the grounds that further substantial changes will not be possible. This independent assessment will usually be external, independent review for both a substantial grant award from a non-commercial or government funder and ethics approval.
